# Hydrogen Peroxide Regulates Butterfly Wing Eyespot Color Patterns

**DOI:** 10.3390/insects17101032

**Published:** 2026-10-08

**Authors:** Yugo Nakazato, Joji M. Otaki

**Affiliations:** The BCPH Unit of Molecular Physiology, Department of Chemistry, Biology and Marine Science, Faculty of Science, University of the Ryukyus, Nishihara 903-0213, Okinawa, Japan

**Keywords:** butterfly wing, color pattern, cuticle, eyespot, hydrogen peroxide, mechanobiology, reactive oxygen species

## Abstract

Butterfly wing color patterns are determined during the early pupal stage. It has been proposed that the interactions between wing epidermal cells and the facing inner cuticle surface may initiate signal transduction pathways that determine cell fate regarding presumptive scale colors. Here, we hypothesized that hydrogen peroxide plays a key role in color pattern determination. To examine this hypothesis, we pharmacologically treated fresh pupae of the Blue Pansy butterfly *Junonia orithya* using two application methods to experimentally manipulate the levels of hydrogen peroxide in pupal wing tissues. When systemically injected into pupae, hydrogen peroxide reduced eyespot size, and two scavengers of hydrogen peroxide, catalase and dimethylthiourea (DMTU), increased eyespot size. When topically applied to pupal wing tissues, hydrogen peroxide induced severe color pattern modifications, catalase increased eyespot size, and a cuticle inhibitor, fluorescent brightener 28 (FB28), reduced eyespot size. Mechanical load tests revealed that the level of hydrogen peroxide influences the cuticle hardness of the pupal body. These results suggest that hydrogen peroxide regulates eyespot color patterns in butterfly wings and that this regulation may be mediated by modulating the chemical and mechanical properties of the facing cuticle during the early pupal stage.

## Simple Summary

We studied butterfly wing color patterns, which are determined during the early pupal stage. Pupal wing tissues are composed of an epidermal cell layer facing the inner surface of the cuticle. Wing epidermal cells differentiate into scale-building cells, and scale colors are likely determined through cell–cuticle interactions. To test the hypothesis that hydrogen peroxide plays a key role in color pattern determination, we pharmacologically manipulated the level of hydrogen peroxide in pupal wing tissues. High levels of hydrogen peroxide reduced eyespot size, whereas low levels of hydrogen peroxide increased eyespot size. The level of hydrogen peroxide affected the hardness of the pupal cuticle. These results suggest the importance of hydrogen peroxide in eyespot color patterning in butterfly wings. Hydrogen peroxide may function by modulating the chemical and mechanical properties of the facing cuticle during the early pupal stage.

## 1. Introduction

In 1924, Spemann and Mangold published an influential study on how undifferentiated cells determine their developmental fates to differentiate into specialized cells [1]. Spemann and Mangold discovered that a group of cells called the developmental organizer is responsible for instructing surrounding undifferentiated cells to commit to particular developmental fates [1,2]. This process is called induction. Since then, for more than 100 years, experimental embryologists and developmental biologists have been investigating the mechanism of cell-fate determination based on the assumption that organizing cells secrete a diffusible signaling molecule called a morphogen to instruct undifferentiated cells for positional information. To do so, morphogens are supposed to form a stable concentration gradient over distances. Several morphogens, such as TGFβ family proteins and Wnt family proteins, have been identified in various biological systems [3,4,5,6,7,8,9,10,11,12,13]; however, how these molecular morphogens form a concentration gradient and how cells receive and interpret positional information remain unclear.

In the butterfly wing system, undifferentiated epidermal (epithelial) cells in the early pupal wing tissue differentiate into scale-building cells (or simply scale cells) through two consecutive cell divisions, which produce pigmented and/or structural scales [14,15]. Because the results of cell differentiation are expressed as a scale color per cell, the butterfly wing system is excellent for studying cell-fate determination. Many nymphalid butterflies have large eyespot patterns on their adult wings. The center of a prospective eyespot in the early pupal stage functions as a developmental organizer that instructs undifferentiated epidermal cells to produce a specific scale color [15,16,17,18,19,20].

From the perspective of scale cell differentiation, transcription factors such as Distal-less (Dll) and Antennapedia (Antp) are likely responsible for determining the prospective eyespot focus [18,21,22,23]. Molecular morphogens, such as Wingless (Wg), Decapentaplegic (Dpp), and Hedgehog (Hh), also appear to be important for butterfly wing color pattern development [24,25,26]. Although the importance of these signaling proteins in development cannot be overemphasized, it has been argued that the diffusion of such molecules alone cannot explain the real butterfly eyespot diversity in terms of shape and size [15]. According to the conventional gradient model, morphogenic signals in butterfly pupal wing tissues must spread quickly and establish stable gradients over hundreds of cells within a limited time span after pupation [27,28,29,30,31,32]. Moreover, the putative gradients must be adjusted in response to the entire wing size so that the positional information is correctly provided, irrespective of the wing size [33]. Because these and other points cannot be easily explained by the conventional gradient model, we reasoned that other signaling systems may have been overlooked by developmental biologists.

We have been studying the butterfly wing developmental system on the hypothesis that small universal chemicals, including simple ions and low-molecular-weight compounds, function as developmental signals in wing color pattern determination, as they can spread with high velocity to cover a large area within a relatively short time. Accordingly, we have previously shown that calcium ions play an important role in wing scale development [34]. We have detected calcium waves and oscillations in pupal wing tissues using real-time in vivo imaging [34,35]. Reactive oxygen species (ROS), including hydrogen peroxide (H_2_O_2_), are additional candidates for signaling chemicals. ROS are known as oxidative stressors at relatively high concentrations owing to their strong oxidative ability, which could result in fatal damage to biological macromolecules and organelles [36,37,38]. On the other hand, certain ROSs are known to function as signaling molecules at relatively low concentrations [39,40,41,42,43,44,45,46,47,48]. For example, hydrogen peroxide waves run in plant leaves [49], and the membrane receptors for ROS, including hydrogen peroxide, have been discovered in plants [50]. These findings suggest that hydrogen peroxide can function as a small signaling chemical in living organisms.

A recent study revealed that NADPH oxidase (NOX) is a vital driver of butterfly wing color pattern formation and wing morphology [51]. Considering that NOX produces hydrogen peroxide, its inhibition leads to a decrease in the amount of hydrogen peroxide, which may harden or soften the cuticle because hydrogen peroxide is involved in cuticle sclerotization. Importantly, the cuticle layer is situated on the top of epithelial cells, and it has been shown that the mechanical interactions between epithelial cells and cuticles are also crucial for wing color pattern determination [52,53]. In accordance with these findings, applying a polysaccharide chitin-binding inhibitor, FB28 (fluorescent brightener 28), to the early pupa induces dramatic color pattern modifications [53]. Chitin-binding disruption also results in a reduction in cuticle hardness. The apical side of the pupal wing epithelial cells forms a continuous epithelial sheet, and the cuticle layer corresponding to the organizer region of the pupal wing exhibits a thick cuticular morphology compared with the surrounding regions, called the pupal cuticle focal spot (or simply, cuticle spot), suggesting the importance of cuticle in the organizer’s function [54,55].

To test the hypothesis that hydrogen peroxide plays a key role in color pattern formation in connection with morphogenic signaling and cuticle mechanochemistry, we conducted pharmacological experiments using the Blue Pansy butterfly *Junonia orithya*. First, we used the injection method to change the hydrogen peroxide concentrations through systemic application. Second, we performed the sandwich method to change the hydrogen peroxide concentrations in the wings through topical application. Third, we performed a mechanical load test to examine the relationship between hydrogen peroxide concentration and cuticle hardness. The usefulness of these methodologies has already been demonstrated in our previous studies [51,53]. We also used an additional species, the Peacock Pansy butterfly *Junonia almana*, to strengthen the results obtained in *J. orithya*.

## 2. Materials and Methods

### 2.1. Butterflies

The Blue Pansy butterfly *J. orithya* was used as the main target species in this study. This butterfly is popular in Okinawa-jima Island, Okinawa Prefecture, Japan, where there is no obligatory permission on its collection and use for biological experiments. Female butterflies were collected from the Nishihara Campus of the University of the Ryukyus, Okinawa, Japan. The collected female butterflies were kept in a grass tank (300 mm × 300 mm × 300 mm) together with the natural host plant *Phyla nodiflora* for oviposition. The larvae were reared with the natural host plant *Plantago asiatica* in a plastic container at ambient temperature (approximately 26 °C) under 16 h (light) and 8 h (dark) lighting conditions.

The Peacock Pansy butterfly *J. almana* was also used as an additional target species (Appendix A) to confirm the results of *J. orithya*. The butterfly collection sites, egg collection method, and larval rearing conditions of *J. almana* were the same as those of *J. orithya*, except that we used the host plant *Ruellia simplex* for the oviposition and larval rearing of *J. almana*.

Multiple females were kept in a single glass tank for egg collection; however, most often, only one female laid many eggs. We considered the individuals from these eggs as a sibling group, and the experimental results were compared between the no-treatment (NT) group and the treatment group within the sibling group. For each rearing and experimental treatment, we recorded the number of treated individuals and the number of successful eclosions to obtain the eclosion rate (ER), which may also be regarded as the survival rate (Supplementary Materials Tables S1 and S2). ERs were used to confirm the normal rearing process and to evaluate the toxicity of the experimental treatment.

### 2.2. Nomenclature

**Figure 1:**
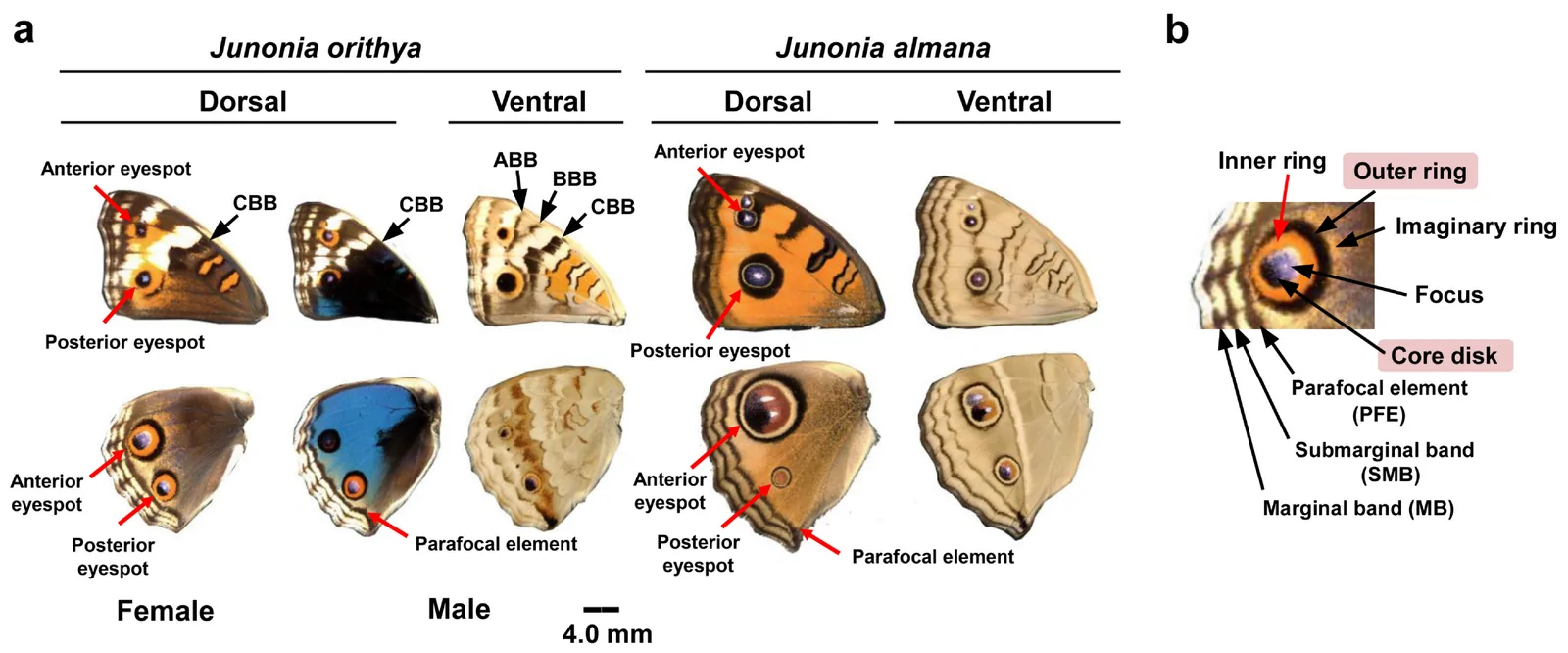
Butterfly wing color patterns of the two species used in this study. (**a**) Color patterns of *J. orithya* and *J. almana*. There are two eyespots on the forewings and hindwings, called the anterior and posterior eyespots. The distal band from the eyespot is the parafocal element, which belongs to the same symmetry system as the eyespot. There are two eyespots on each wing of *J. almana*. However, the anterior eyespots in *J. almana* are fusion eyespots with double foci. The black bands on the ventral forewing of *J. orithya* are temporarily named the anterior black band (ABB), the boundary black band (BBB), and the central black band (CBB), for simplicity. (**b**) Sub-elements and peripheral elements of the eyespot.

The nomenclature of the important color pattern elements on the wings of *J. orithya* and *J. almana* is shown in Figure 1a. The former species is sexually dimorphic in terms of dorsal color patterns, and we analyzed them separately for the two sexes. There are two eyespots per wing surface in *J. orithya* and *J. almana*. These eyespots are referred to as the anterior and posterior eyespots. The center of the eyespot on an adult wing corresponds to the pupal developmental organizer. The anterior eyespot on the dorsal hindwing is occasionally very small or nonexistent in males of *J. orithya*. Therefore, we analyzed both the anterior and posterior eyespots in females but often only the posterior eyespot in males. An eyespot is mainly composed of a core disk and an outer ring, with or without a blue or white focus at the center (Figure 1b).

### 2.3. Experimental Treatments

For systemic applications, 2.0 μL of liquid was injected into the abdomen of a fresh pupa (henceforth called the injection method) within 2 h to 6 h postpupation in the case of hydrogen peroxide or within 1 h postpupation in the cases of catalase (CAT), dimethylthiourea (DMTU), VAS2870, and fluorescent brightener 28 (FB28) via an Ito microsyringe MS-05 (Fuji, Shizuoka, Japan). For topical applications, immediately after pupation (within 30 min postpupation), the left forewing was physically lifted, a liquid droplet (0.6 μL) was placed on the surface of the hindwing, and the forewing was carefully placed back to the original position. In this way, a liquid droplet was sandwiched between the forewing and hindwing (henceforth called the sandwich method). The right wing was not treated and was used for comparison.

**Figure A1:**
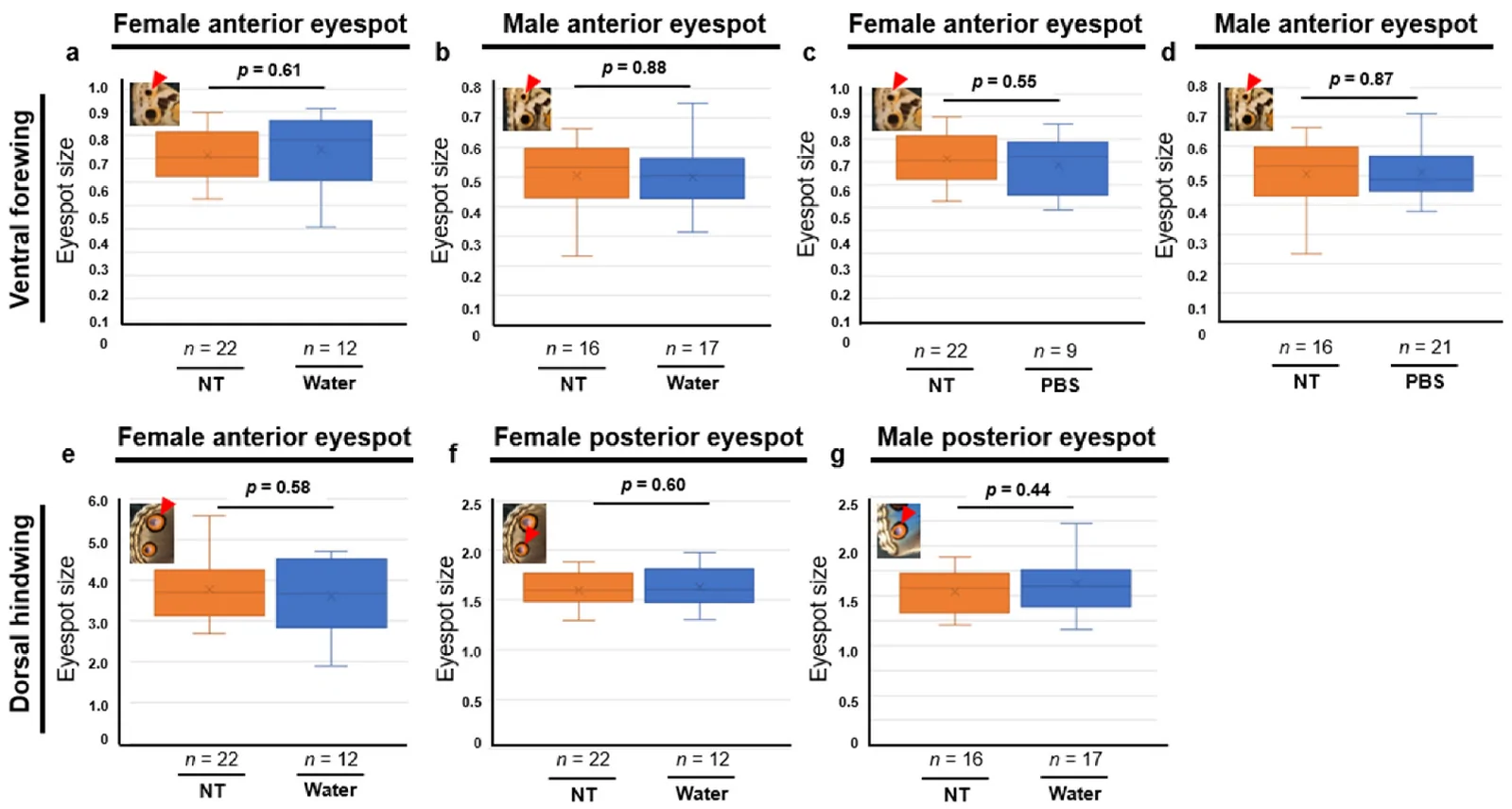
No effect of solvents (deionized water and PBS) on eyespot size. The arrowhead in the inset image indicates the eyespot of interest in each panel. (**a**–**d**) Ventral forewing. (**a**) Water on the female anterior eyespot. (**b**) Water on the male anterior eyespot. (**c**) PBS on the female anterior eyespot. (**d**) PBS on the male anterior eyespot. (**e**–**g**) Dorsal hindwing. (**e**) Water on the female anterior eyespot. (**f**) Water on the female posterior eyespot. (**g**) Water on the male posterior eyespot.

In this study, we performed procedural negative controls using blank solvents (water and PBS) for the injection method (Appendix A, Figure A1), although this procedure has been well established since 1998 [56]. Controls for DMSO have been performed in previous studies [51,57,58]. We did not perform procedural negative controls with blank solvents for the sandwich method in this study, but they were performed recently in our laboratory [51].

Fresh pupae were subjected to a mechanical load test using a Lutron Electronics Enterprise Fruit Hardness Tester FR-5105 (Taipei, Taiwan, Republic of China) in accordance with a previous study [53]. To do so, a pupa was placed on the flat hard surface of the laboratory bench ventral side up. A gauge probe was placed vertically at a specific position on the surface of the fresh pupa and was slowly pushed down manually until the treated pupa ruptured. The peak hold mode was used to obtain the maximal force values at rupture, which were considered pupal hardness. Hardness was expressed in gram force (gf).

### 2.4. Chemical Compounds

Hydrogen peroxide (FUJIFILM Wako Chemicals, Osaka, Japan), catalase (FUJIFILM Wako Chemicals), and DMTU (Angene Chemical, Nanjing, China) were used in this study. Hydrogen peroxide and DMTU were diluted in deionized water, and catalase was diluted in phosphate-buffered saline (PBS). Catalase [59,60,61,62] and DMTU [63,64,65,66,67] are well-known scavengers of hydrogen peroxide. In addition, VAS2870 (Cayman Chemical, Ann Arbor, MI, USA) was diluted in DMSO (dimethyl sulfoxide) (Wako Pure Chemical Industries, Osaka, Japan) and used for injections. VAS2870 is one of the NOX inhibitors [68,69,70,71,72]. For comparison, we used FB28 (Sigma-Aldrich, St. Louis, MO, USA). FB28 is known to bind to chitin (a major component of the cuticle) and other polysaccharides [73,74]. It has been used to detect chitin in the fruit fly [75] and butterfly [53,76]. FB28 effectively induces characteristic color pattern changes called temperature-shock type (TS-type) modifications [53]. The final concentrations used in applications were as follows: H_2_O_2_ (12%, 15%, and 20% for systemic application; 0.3%, 3%, and 6% for topical application; 12% for mechanical load test), DMTU (216.08, 432.16, 10.804, 108.04, and 1080.40 mM for systemic application; 1080.40 mM for topical application; 218.08 mM for mechanical load test), catalase (80 and 800 ng/mL for systemic and topical applications; 80 ng/mL for mechanical load test), FB28 (7.5% for systemic and topical applications and mechanical load test), and VAS2870 (27.75 mM for mechanical load test).

### 2.5. Experimental Evaluations

We acquired wing images of adult butterflies using a Keyence Digital Microscope VHX-7000 (Osaka, Japan). All wing surfaces of the butterflies that were included in this study are shown in Supplementary Materials Figure S1 (*J. orithya*) and Figure S2 (*J. almana*). To observe the wing veins, we used deep contrast images acquired by the VHX-7000, which highlighted the height information, making the visual identification of wing veins easier. For qualitative analysis, we looked for color pattern elements that changed in position and shape in accordance with the nymphalid ground plan. For quantitative analysis, the eyespot area was defined and measured using the brightness function of the VHX-7000. To standardize the eyespot area values in accordance with the whole wing size, the distance between the two wing veins where a given eyespot was present was measured using ImageJ 1.54g (National Institute of Health, Bethesda, MD, USA). To secure a standardized eyespot size, the eyespot area value was divided by the wing vein distance squared. This method is based on the assumption that the wing size is tightly correlated with the compartment size, which is represented by the wing vein distance squared. This method has been employed in previous studies [51,57,58]. Statistical evaluation (unpaired two-sided Student’s *t*-test or Welch’s *t*-test after *F*-test) of eyespot size was performed using Microsoft Excel (Microsoft Office 365) and JSTAT 16.1 (Sato, M., Keiyu Hospital, Yokohama, Japan) on the assumption that there would be no difference between the treated and untreated groups (null hypothesis). For multiple comparisons, single comparison results were shown, but Bonferroni-corrected *p*-values were mentioned together with the original ones. We calculated Cohen’s *d* for effective size. Because there are many variations in the wing color patterns of *J. orithya*, pharmacological results were compared within a sibling group.

## 3. Results

### 3.1. Systemic Applications of Hydrogen Peroxide and Catalase: Ventral Forewing Eyespot

To examine the effects of hydrogen peroxide on wing color patterns, we first performed systemic applications (i.e., injections) of hydrogen peroxide into the pupae. After eclosion, we visually noticed the size reduction in the anterior eyespot on the ventral forewing, which is subjected to quantitative analysis below.

**Figure 2:**
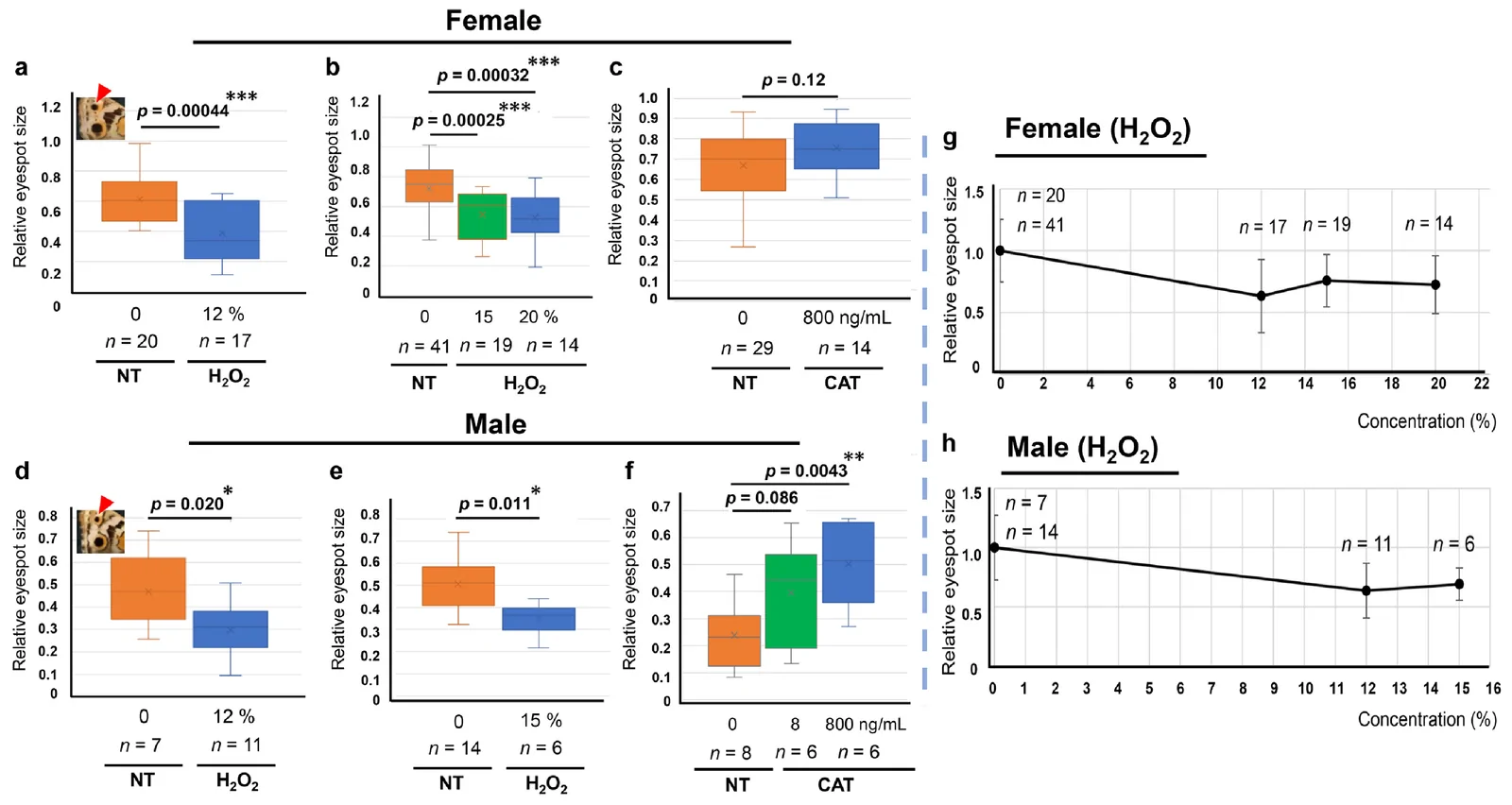
Effects of hydrogen peroxide (H_2_O_2_) and catalase (CAT) on the ventral forewing eyespot size. *: *p* < 0.05, **: *p* < 0.01, ***: *p* < 0.001. NT: no treatment. Arrowhead in inset image indicates the eyespot of interest in each panel. (**a**–**c**) Quantitative evaluation of the relative anterior eyespot size on the female ventral forewing. (**a**) Hydrogen peroxide (12%). (**b**) Hydrogen peroxide (15% and 20%). For Bonferroni correction for multiple comparisons, these *p*-values are to be doubled. (**c**) Catalase (800 ng/mL). (**d**–**f**) Quantitative evaluation of the relative anterior eyespot size on the male ventral forewing. (**d**) Hydrogen peroxide (12%). (**e**) Hydrogen peroxide (15% and 20%). (**f**) Catalase (8 ng/mL and 800 ng/mL). For Bonferroni corrections for multiple comparisons, these *p*-values are to be doubled. (**g**) Dose–response of relative eyespot size on hydrogen peroxide in females. Relative eyespot size showed no dose dependence on hydrogen peroxide concentration past the threshold of 12% in females. (**h**) Dose–response of relative eyespot size on hydrogen peroxide in males. Relative eyespot size showed no dose dependence on hydrogen peroxide past the threshold of 12% in males.

In females, compared with the no-treatment group (*n* = 20), the hydrogen peroxide (12%) group (*n* = 17) showed a significantly smaller eyespot size (*p* = 0.00044; *d* = 1.3) in a sibling group (Figure 2a). In a different sibling group, the hydrogen peroxide (15%) group (*n* = 19) showed a significantly smaller eyespot size (*p* = 0.00025; *d* = 1.1) than the no-treatment group (*n* = 41) (Figure 2b). In the same sibling group, the hydrogen peroxide group (20%) (*n* = 14) showed a significantly smaller eyespot size (*p* = 0.00032; *d* = 1.2) (Figure 2b). We then tested the effect of catalase as a hydrogen peroxide scavenger via systemic application in females. Compared with the no-treatment group (*n* = 41), the catalase group (*n* = 14) did not show a significant difference in eyespot size in females (*p* = 0.12; *d* = 0.53) (Figure 2c).

In males, compared with the no-treatment group (*n* = 7), the hydrogen peroxide (12%) group (*n* = 11) showed a significantly smaller eyespot (*p* = 0.020; *d* = 1.3) in a sibling group (Figure 2d), as in females. In a different sibling group, the hydrogen peroxide (15%) group (*n* = 6) showed significantly smaller eyespot size (*p* = 0.011; *d* = 1.5) than the no-treatment group (*n* = 14) (Figure 2e). We then tested the effects of catalase in males. In a sibling group, compared with the no-treatment group (*n* = 8), the catalase (800 ng/mL) group (*n* = 6) showed significantly larger eyespot size (*p* = 0.0043; *d* = 2.0), but the catalase (8 ng/mL) group (*n* = 6) did not show a significant difference (*p* = 0.086; *d* = 1.1) (Figure 2f).

Based on the above data, we constructed dose–response curves for females and males treated with hydrogen peroxide. The dose dependence of the eyespot in response to hydrogen peroxide was not clear at high concentrations above 12% (Figure 2g,h). It can be understood that hydrogen peroxide reduced eyespot size in the dorsal forewing anterior eyespot in both females and males. A scavenger of hydrogen peroxide, catalase, increased eyespot size only in males.

**Figure A2:**
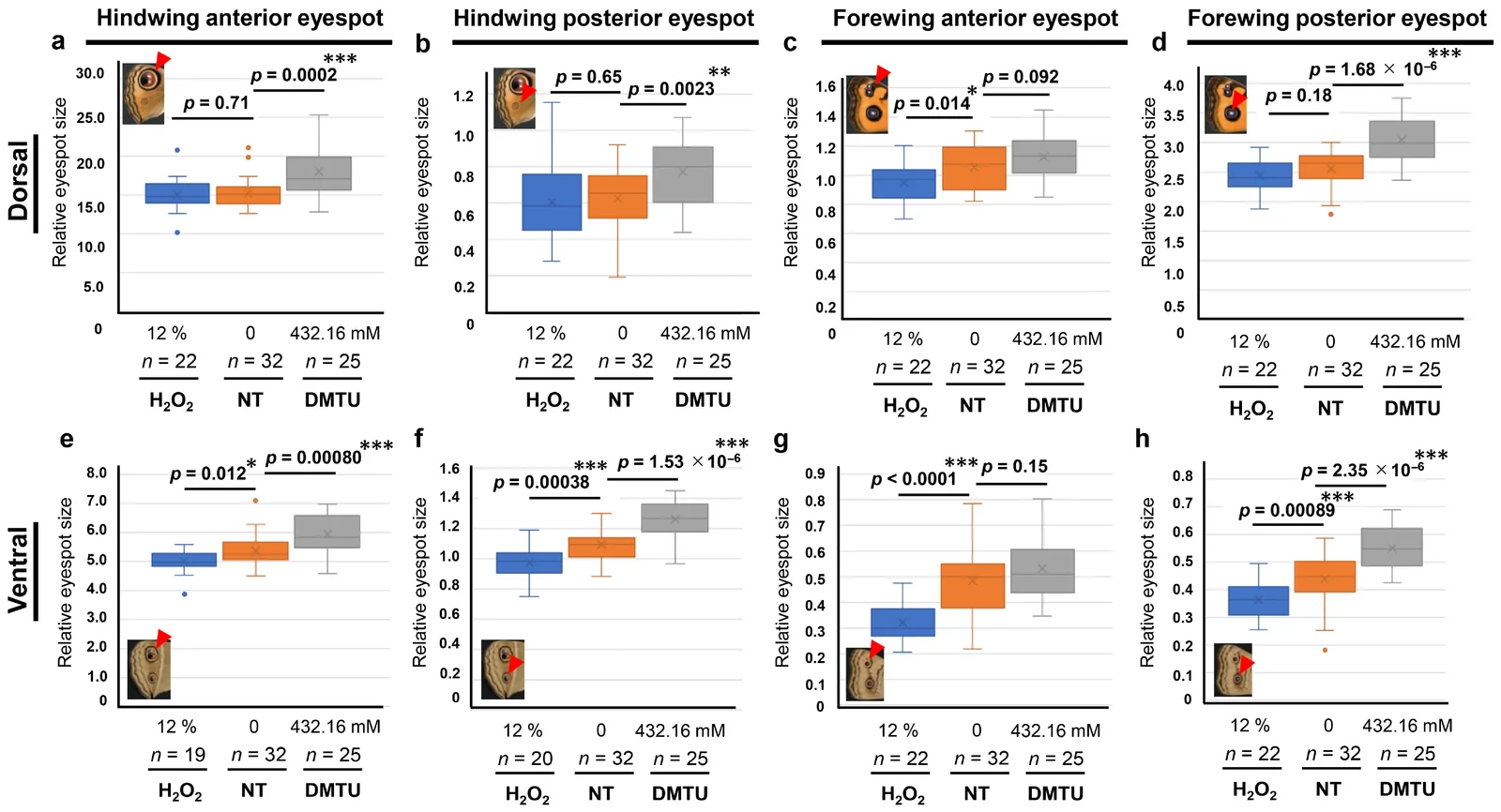
Effects of hydrogen peroxide and DMTU on eyespot size in *J. almana.* *: *p* < 0.05, **: *p* < 0.01, ***: *p* < 0.001. For Bonferroni corrections for multiple comparisons, these *p*-values are to be doubled. Arrowhead in inset image indicates the eyespot of interest in each panel. (**a**–**d**) Dorsal wings. (**a**) Hindwing anterior eyespot. (**b**) Hindwing posterior eyespot. (**c**) Forewing anterior eyespot. (**d**) Forewing posterior eyespot. (**e**–**h**) Ventral wings. (**e**) Hindwing anterior eyespot. (**f**) Hindwing posterior eyespot. (**g**) Forewing anterior eyespot. (**h**) Forewing posterior eyespot.

We confirmed that the solvents for hydrogen peroxide (water) and catalase (PBS) did not induce any changes in eyespot size (Appendix A, Figure A1). Furthermore, we tested the effects of hydrogen peroxide on eyespot size in *J. almana* and obtained results regarding the size-reducing effect of hydrogen peroxide (Appendix A, Figure A2), as in *J. orithya*.

### 3.2. Systemic Applications of Hydrogen Peroxide: Dorsal Hindwing Eyespot

Among the individuals treated with hydrogen peroxide as described above, we visually noticed a large outer ring on the dorsal hindwings. Accordingly, we analyzed the outer ring of the dorsal eyespots of the hindwings.

**Figure 3:**
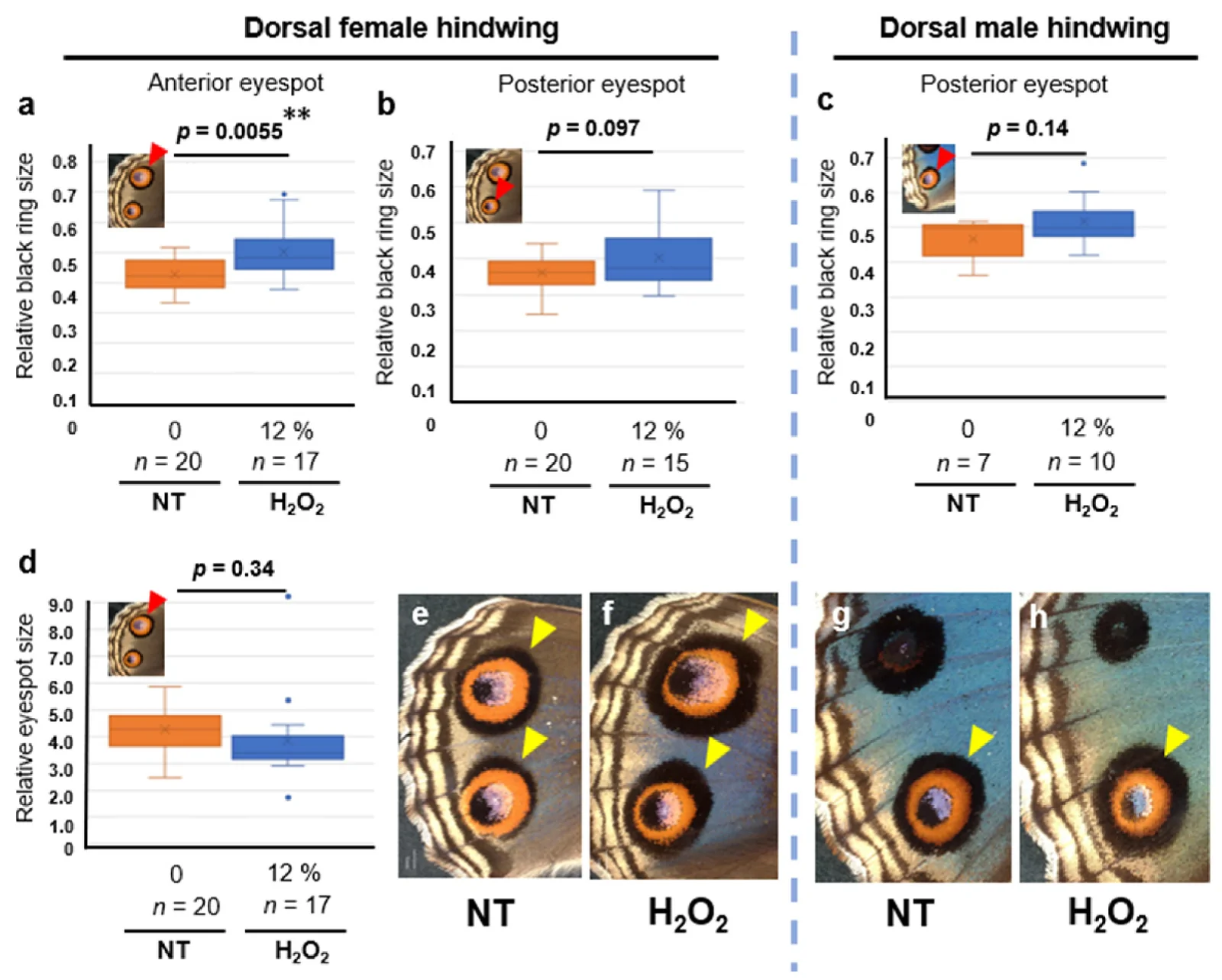
Effects of systemic application of hydrogen peroxide (H_2_O_2_) on the outer ring area. **: *p* < 0.01. NT: no treatment. The arrowhead in the inset image indicates the eyespot of interest in each panel. (**a**–**c**) Quantitative evaluation of the area of the eyespot outer black ring. (**a**) Anterior eyespot outer black ring on the female dorsal hindwing. (**b**) Posterior eyespot outer black ring on the female dorsal hindwing. (**c**) Posterior eyespot outer black ring on the male dorsal hindwing. (**d**) Relative anterior eyespot size on the female dorsal hindwing. (**e**) Female dorsal hindwing (no treatment). (**f**) Female dorsal hindwing (hydrogen peroxide). (**g**) Male dorsal hindwing (no treatment). (**h**) Male dorsal hindwing (hydrogen peroxide).

In females, the outer ring of the anterior eyespot was significantly larger in the hydrogen peroxide group (*n* = 17) than in the no-treatment group (*n* = 20) (*p* = 0.0055; *d* = 1.1) (Figure 3a), although the outer ring of the posterior eyespot in the treatment group did not show a significant change (*n* = 15) in comparison to the no-treatment group (*n* = 20) (*p* = 0.097; *d* = 0.66) (Figure 3b). In males, there was no significant difference between the treatment group (*n* = 10) and the no-treatment group (*n* = 7) (*p* = 0.14; *d* = 0.81) (Figure 3c). Interestingly, eyespot area sizes themselves were not significantly different between the treatment group (*n* = 17) and the no-treatment group (*n* = 20) in the female anterior eyespot (*p* = 0.34; *d* = 0.34) (Figure 3d). Although the quantitative results were statistically significant only on the anterior eyespot of females, the effect of hydrogen peroxide on the outer ring may be valid for all three eyespots to some extent, both in females (Figure 3e,f) and in males (Figure 3g,h) based on visual images.

**Figure A3:**
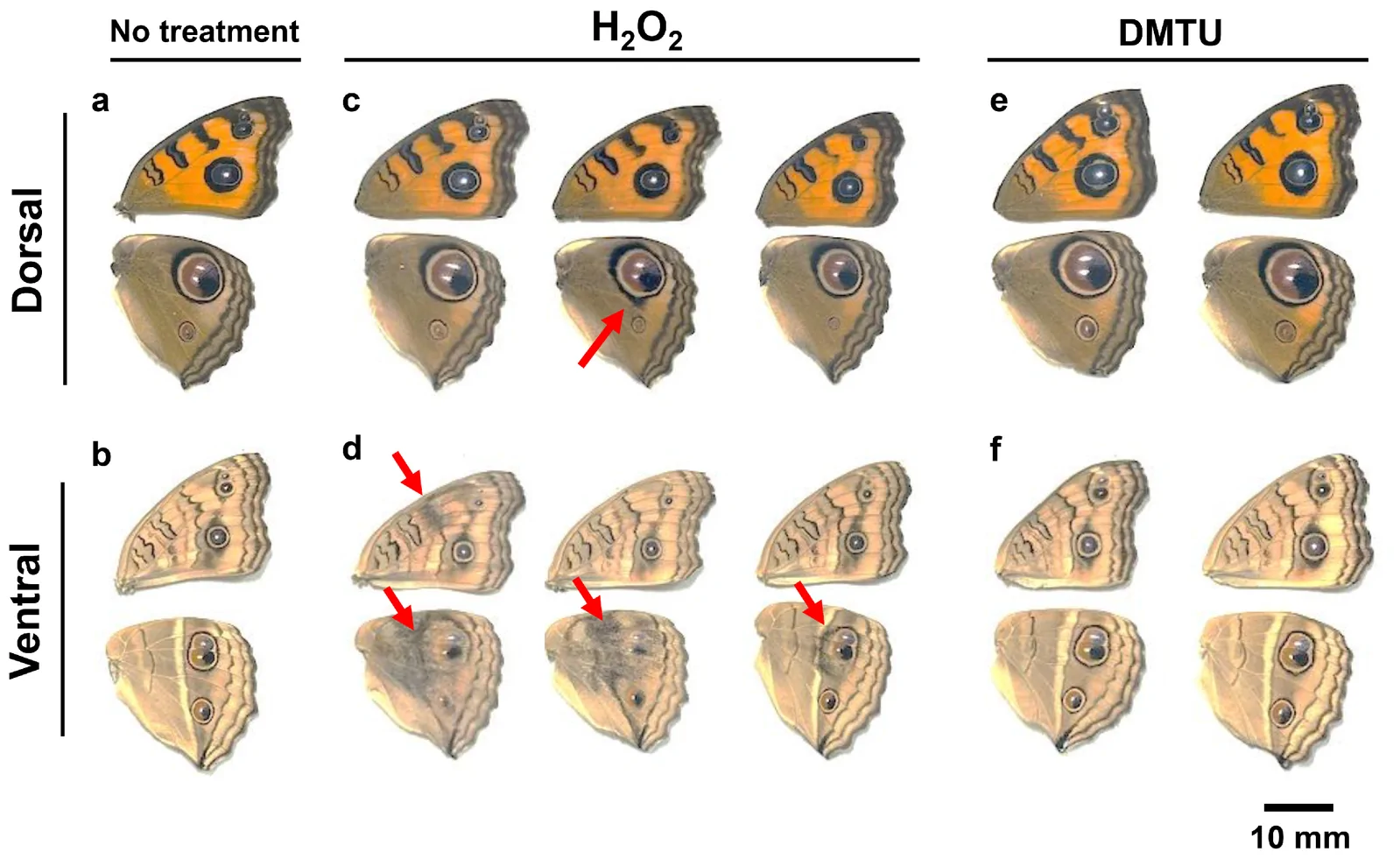
Color pattern modifications induced by hydrogen peroxide and DMTU in *J. almana*. The arrows indicate the modifications. (**a**,**b**) Dorsal and ventral wings of an untreated individual. (**c**,**d**) Dorsal and ventral wings of three individuals treated with hydrogen peroxide. (**e**,**f**) Dorsal and ventral wings of two individuals treated with DMTU.

Overall, hydrogen peroxide appeared to induce outer ring expansion in the hindwing dorsal eyespots. Because the eyespot size did not change in response to hydrogen peroxide, this response in the outer ring may be similar to, but mechanistically different from, the size-reducing effect of hydrogen peroxide detected previously. Importantly, we confirmed the clear effect of hydrogen peroxide on the outer ring, although qualitatively, in *J. almana* (Appendix A, Figure A3). In *J. almana*, outer ring expansion was observed in the dorsal hindwing eyespot, as in *J. orithya*, but more severe expansion was also observed in the ventral hindwing eyespot (Appendix A, Figure A3).

### 3.3. Systemic Applications of DMTU: Dorsal Hindwing Eyespot

**Figure 4:**
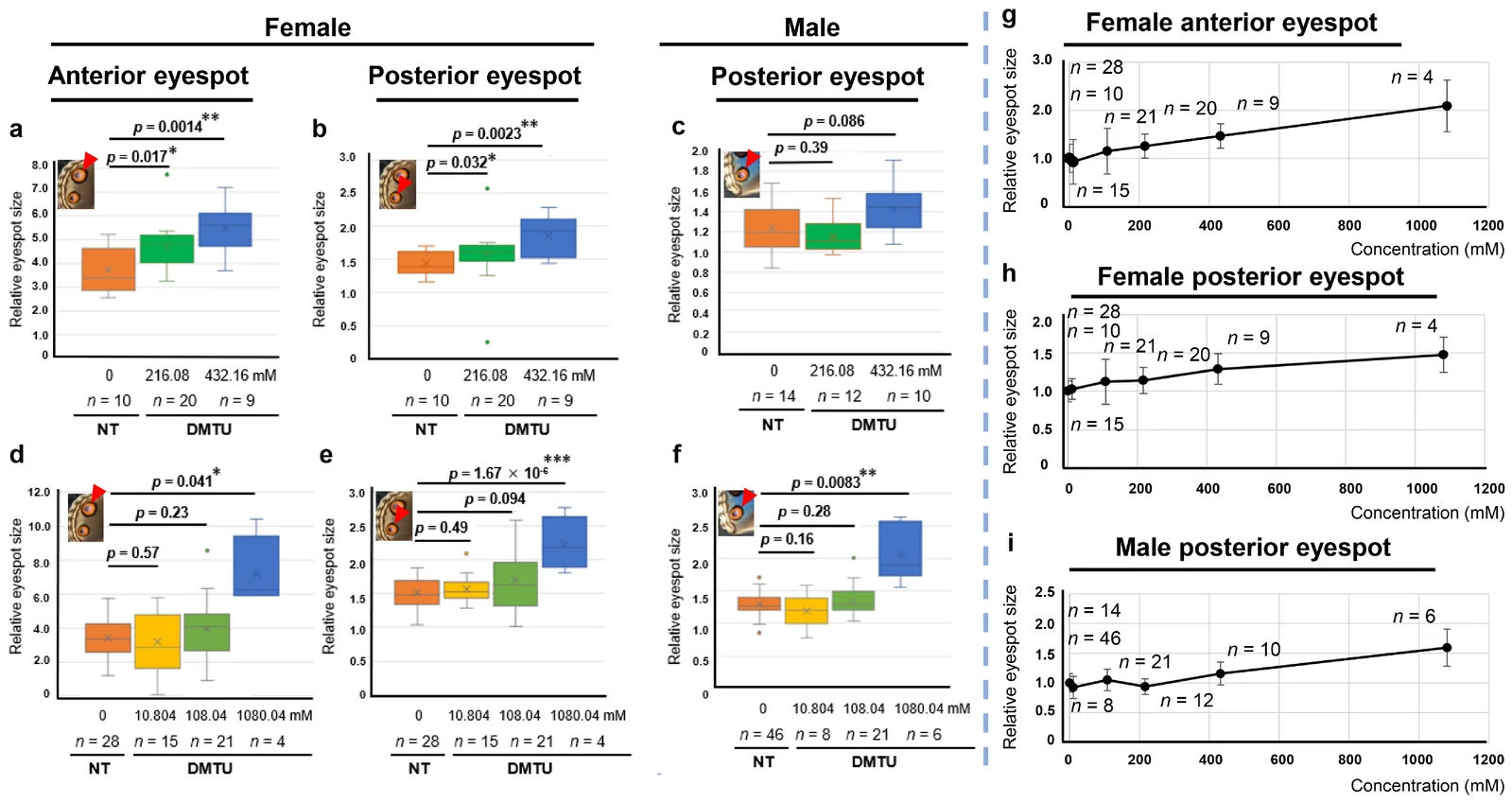
Effects of DMTU on the dorsal hindwing eyespot size. *: *p* < 0.05, **: *p* < 0.01, ***: *p* < 0.001. NT: no treatment. Arrowhead in inset image indicates the eyespot of interest in each panel. For Bonferroni corrections for multiple comparisons, these *p*-values are to be doubled or tripled. (**a**–**c**) Quantitative evaluation of the relative eyespot size on the dorsal hindwing with moderate concentrations of DMTU (216.08 mM and 432.16 mM). (**a**) Female anterior eyespot. (**b**) Female posterior eyespot. (**c**) Male posterior eyespot. (**d**–**f**) Quantitative evaluation of the relative eyespot size on the dorsal hindwing with low and high concentrations of DMTU (10.804 mM, 108.04 mM, and 1080.04 mM). (**d**) Female anterior eyespot. (**e**) Female posterior eyespot. (**f**) Male posterior eyespot. (**g**) Dose–response of relative eyespot size on DMTU in the female anterior eyespot. (**h**) Dose–response of the female posterior eyespot on DMTU. (**i**) Dose–response of the male posterior eyespot on DMTU.

Given that hydrogen peroxide and catalase appeared to show their effects in opposite directions, we tested an additional scavenger of hydrogen peroxide, dimethylthiourea (DMTU), at two different concentrations (216.08 mM and 432.16 mM). The DMTU group (*n* = 20 for 216.08 mM and *n* = 9 for 432.16 mM) had significantly larger eyespots in both 216.08 mM (*p* = 0.017; *d* = 1.0) and 432.16 mM (*p* = 0.0014; *d* = 1.9) than the no-treatment group (*n* = 10) in the female anterior eyespot (Figure 4a). Similarly, in the female posterior eyespot, the DMTU group (*n* = 20 for 216.08 mM and *n* = 9 for 432.16 mM) had significantly larger eyespots at both 216.08 mM (*p* = 0.032; *d* = 0.90) and 432.16 mM (*p* = 0.0023; *d* = 1.8) than the no-treatment group (*n* = 10) (Figure 4b). In contrast, in the male posterior eyespot, the DMTU group (*n* = 12 for 216.08 mM and *n* = 10 for 432.16 mM) did not show a significant difference in size at either 216.08 mM (*p* = 0.39; *d* = 0.36) or 432.16 mM (*p* = 0.086; *d* = 0.78) compared with the no-treatment group (*n* = 14) (Figure 4c).

We further tested two lower concentrations (10.804 mM and 108.04 mM) as well as a higher concentration (1080.40 mM) of DMTU to examine the dose–response of eyespots to hydrogen peroxide. As expected, no significant difference in eyespot size was obtained in the two groups of lower concentrations, 10.804 mM (*n* = 15) (*p* = 0.57; *d* = 0.19) and 108.04 mM (*n* = 21) (*p* = 0.23; *d* = 0.39), in comparison with the no-treatment group (*n* = 28), in the female anterior eyespot (Figure 4d). In contrast, at the high concentration, 1080.40 mM, the DMTU group (*n* = 4) significantly increased eyespot size compared with the no-treatment group (*n* = 28) (*p* = 0.041; *d* = 3.1) (Figure 4d). Similarly, in the female posterior eyespot, no significant difference was observed in the lower concentrations, 10.804 mM (*n* = 15) (*p* = 0.49; *d* = 0.39) and 108.04 mM (*n* = 21) (*p* = 0.094; *d* = 0.56), compared with the no-treatment group (*n* = 28) (Figure 4e). In contrast, the DMTU group significantly increased eyespot size at the high concentration, 1080.40 mM (*n* = 4) (*p* = 1.67 × 10^−6^; *d* = 3.1) (Figure 4e). Similar results were obtained in the male posterior eyespots. Compared with the no-treatment group (*n* = 46), no significant difference was observed in the low concentrations, 10.804 mM (*n* = 8) (*p* = 0.16; *d* = 0.55) and 108.04 mM (*n* = 21) (*p* = 0.28; *d* = 0.33) (Figure 4f). In contrast, the DMTU group significantly increased eyespot size at the high concentration, 1080.4 mM (*n* = 6) (*p* = 0.0083; *d* = 3.7) (Figure 4f).

Based on the data above, we constructed dose–response curves for three eyespots (two female eyespots and one male eyespot). In all cases, eyespot size response appeared to be dose-dependent (Figure 4g–i). However, the male posterior eyespot seemed to be less sensitive to DMTU treatment than female eyespots (Figure 4g–i), suggesting sex-specific differences in eyespot development that reflect the sexual dimorphism of this species.

Overall, DMTU effectively increased eyespot size in a concentration-dependent manner in both sexes of *J. orithya*. We confirmed that the solvent for DMTU (water) did not induce any changes in eyespot size (Appendix A, Figure A1). Furthermore, we confirmed the eyespot-increasing effect of DMTU on *J. almana* (Appendix A, Figure A2).

### 3.4. Systemic Applications of Hydrogen Peroxide, DMTU, and FB28: Qualitative Changes

**Figure 5:**
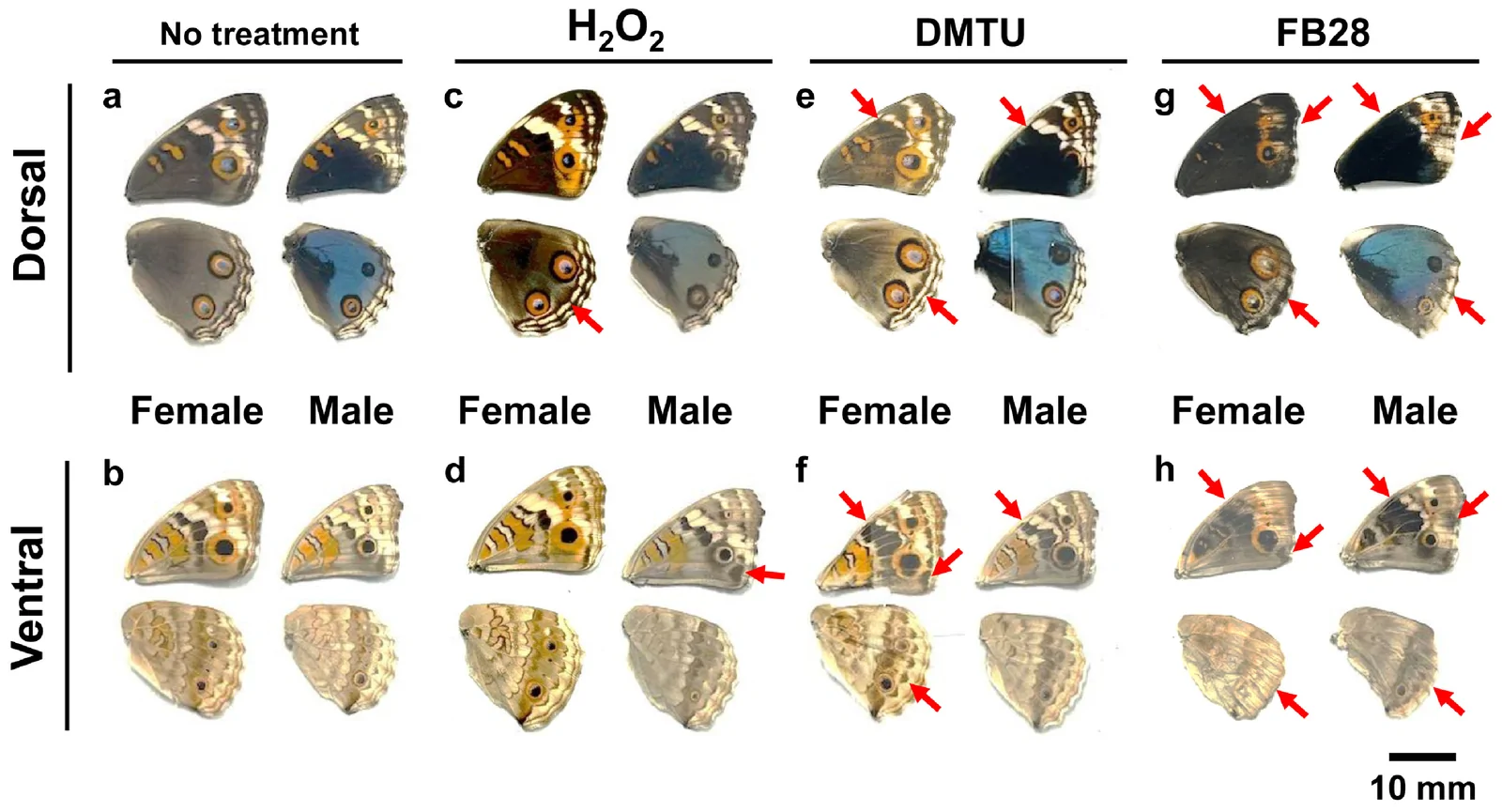
Wing color patterns after systemic application of hydrogen peroxide (H_2_O_2_), DMTU, and FB28. Arrows indicate modified elements (CBB and PFE). (**a**) Dorsal side of no-treatment individuals. (**b**) Ventral side of no-treatment individuals. (**c**) Dorsal side of hydrogen peroxide-injected individuals. (**d**) Ventral side of hydrogen peroxide-injected individuals. (**e**) Dorsal side of DMTU-injected individuals. (**f**) Ventral side of DMTU-injected individuals. (**g**) Dorsal side of FB28-injected individuals. (**h**) Ventral side of FB28-injected individuals.

Here, we examined the qualitative color pattern changes in elements other than eyespots in response to hydrogen peroxide and DMTU. In comparison to the no-treatment group (*n* = 27) (Figure 5a,b), the hydrogen peroxide group (in all concentrations) showed almost no modifications (*n* = 24), with the exception of one individual showing thick PFE on the female dorsal hindwing (Figure 5c). Similarly, four individuals showed thickened PFE on the ventral forewings (Figure 5d). In contrast, compared to the no-treatment group (*n* = 74), the DMTU group (*n* = 10) had two individuals that showed an outward extension of the central black band (CBB) and thickened PFE on the dorsal wing (Figure 5e). Additionally, three individuals showed an outward extension of the central black band (CBB) and thickened PFE on the ventral forewing (Figure 5f). It is interesting that PFE modifications were produced in both the hydrogen peroxide and DMTU treatments, despite their counteracting mechanisms.

For comparison, we repeated systemic applications of FB28. The FB28 group (*n* = 2) showed well-known TS-type modifications, as expected from a previous study [53], with a severe outward extension of the central black band (CBB) and thickened PFE on both the dorsal and ventral wings (Figure 5g,h). These modifications were similar to those shown in the DMTU group in terms of CBB extension. However, DMTU-induced modifications appeared less severe than FB28-induced modifications. The latter exhibited severe modifications in the basal portion of the forewing, which were not detected in DMTU-treated individuals.

In the case of *J. almana*, modifications of the black bands and PFE were not observed after the systemic application of DMTU (Appendix A, Figure A3). However, modifications of the black bands and outer ring of the ventral side were observed after the application of hydrogen peroxide (Appendix A, Figure A3).

### 3.5. Systemic Applications of Hydrogen Peroxide: Wing Vein Development

**Figure 6:**
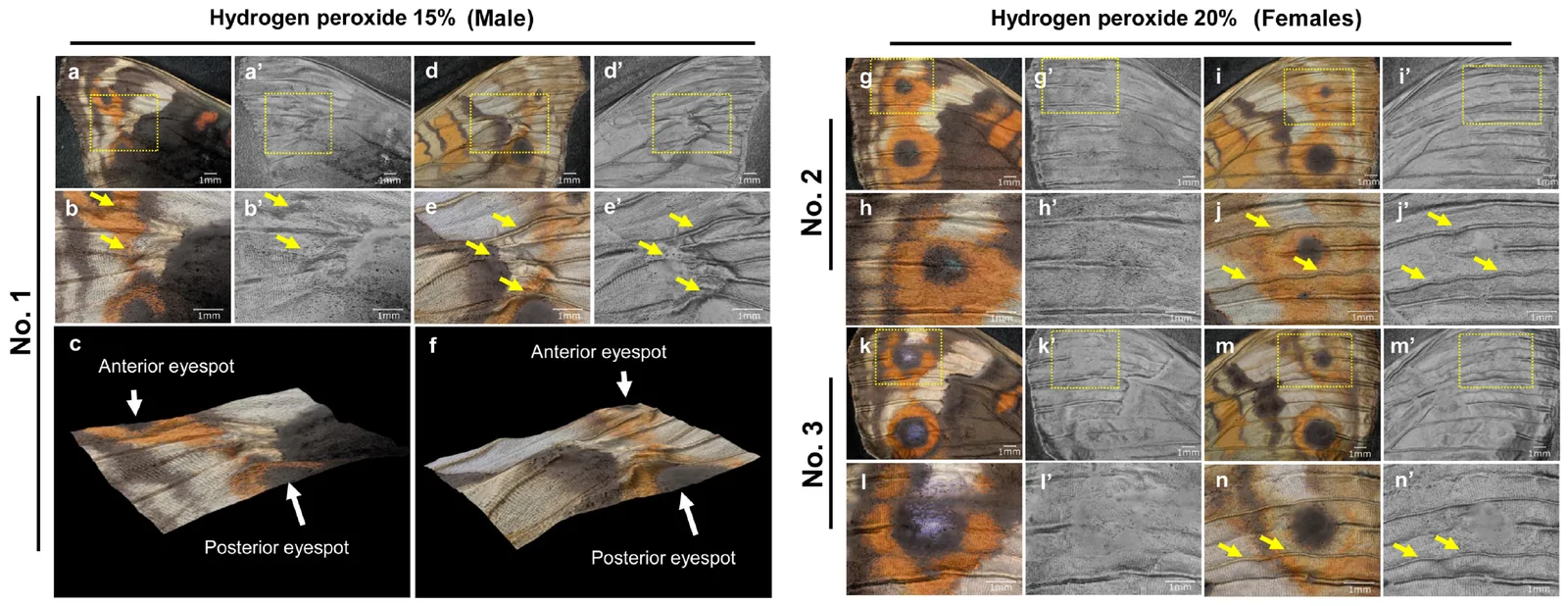
Deep-contrast wing images of wing veins after systemic application of hydrogen peroxide. A pair of color and monochrome images in the same visual region are presented. The yellow rectangular region is magnified in the subsequent panels. Yellow arrows indicate wavy or deformed veins. Three representative individuals (No. 1, No. 2, and No. 3) are shown. (**a**–**f**) Forewing images from a male individual treated with hydrogen peroxide (15%). (**a**) Dorsal forewing. (**b**) High magnification of (**a**). (**c**) Three-dimensional image of (**b**). (**d**) Ventral forewing. (**e**) High magnification of (**d**). (**f**) Three-dimensional image of (**e**). (**g**–**n**) Forewing images from female individuals treated with hydrogen peroxide (20%). (**g**,**k**) Dorsal forewing. (**h**,**l**) High magnification of (**g**,**k**). (**i**,**m**) Ventral forewing. (**j**,**n**) High magnification of (**i**,**m**).

In examining the individuals treated with hydrogen peroxide, we noted that the wing veins were irregularly tortuous in some individuals (Figure 6). In males, both the dorsal and ventral forewing surfaces simultaneously had irregular veins (Figure 6a–f). Three-dimensional images also demonstrated the deformation of the dorsal (Figure 6c) and ventral (Figure 6f) forewing surfaces. In contrast, in females, the dorsal forewings showed only moderate irregularity of the veins (Figure 6g,h,k,l). However, their ventral forewings had extensive irregularities in the veins, especially around the anterior eyespot (Figure 6i,j,m,n).

When a 15% hydrogen peroxide solution was injected, irregular veins were observed in nine of fourteen females and one of two males (62.5% in both sexes). When a 20% hydrogen peroxide solution was injected, irregular veins were observed in nine of nineteen females and three of six males (48.0% in both sexes). No tortuous wing veins were observed when a 12% hydrogen peroxide solution was injected (*n* = 28). No such wing veins were observed in the no-treatment group (*n* = 55). These results demonstrated that hydrogen peroxide affected wing vein development when injected at high concentrations.

### 3.6. Topical Applications of Hydrogen Peroxide

To further understand the effects of hydrogen peroxide on wing color pattern development, we conducted topical applications of hydrogen peroxide on the surfaces between the dorsal hindwing and ventral forewing using the sandwich method. Eyespot size quantification was difficult due to the severe deformation of the eyespots. Thus, here we present descriptive data for phenotypic variability.

**Figure 7:**
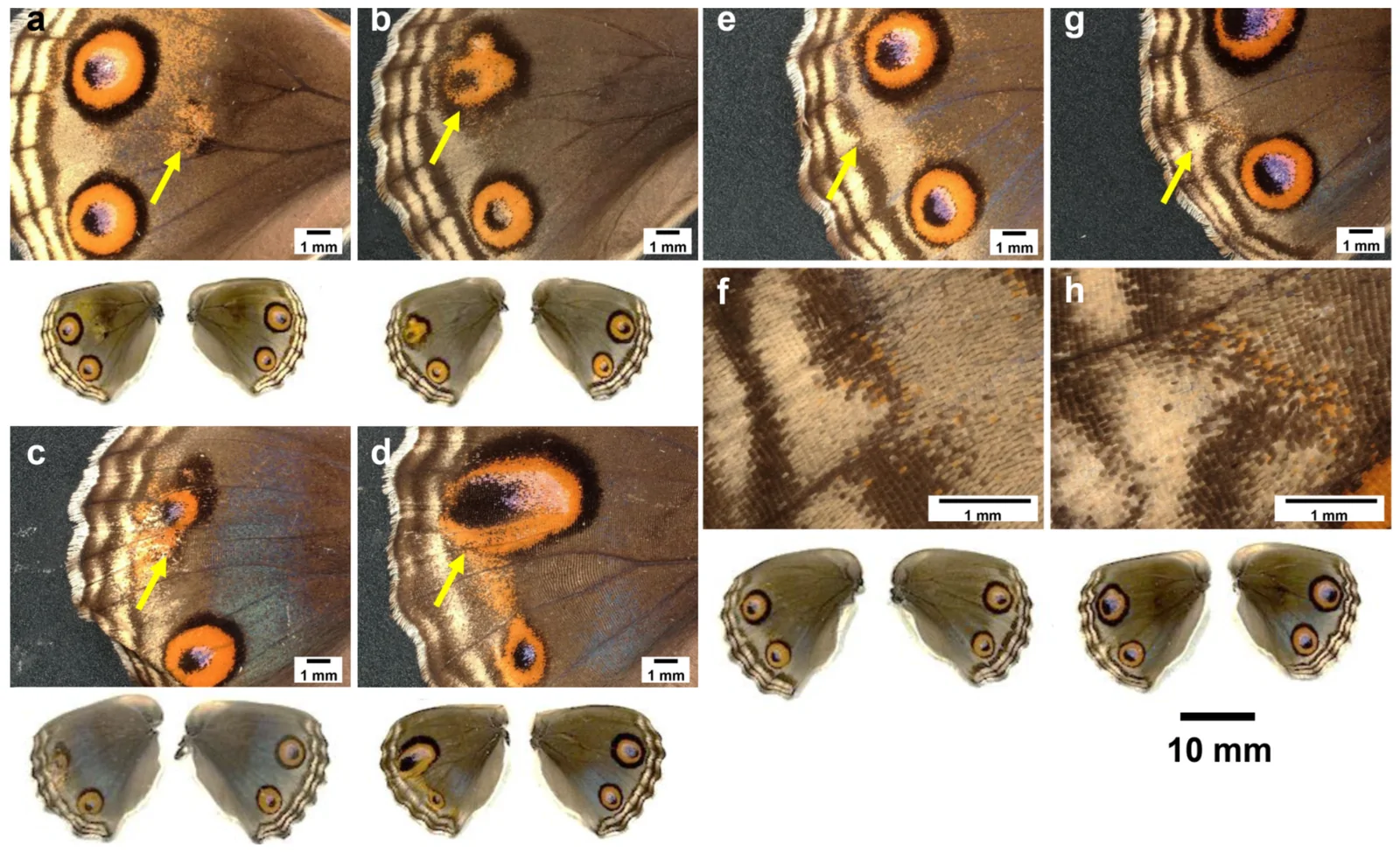
Dorsal hindwing color pattern modifications induced by topical application of hydrogen peroxide (6%) in females. Overall wing color patterns of both the left (treated) and right (untreated) wings are shown below the magnified images. The arrows indicate the modifications. (**a**) Extra orange scales. (**b**) Eyespot deformation. (**c**) Eyespot deformation and size reduction. (**d**) Eyespot size expansion. (**e**,**g**) Minor inward extension of PFE. (**f**,**h**) High-magnification image of (**e**,**g**).

**Figure A4:**
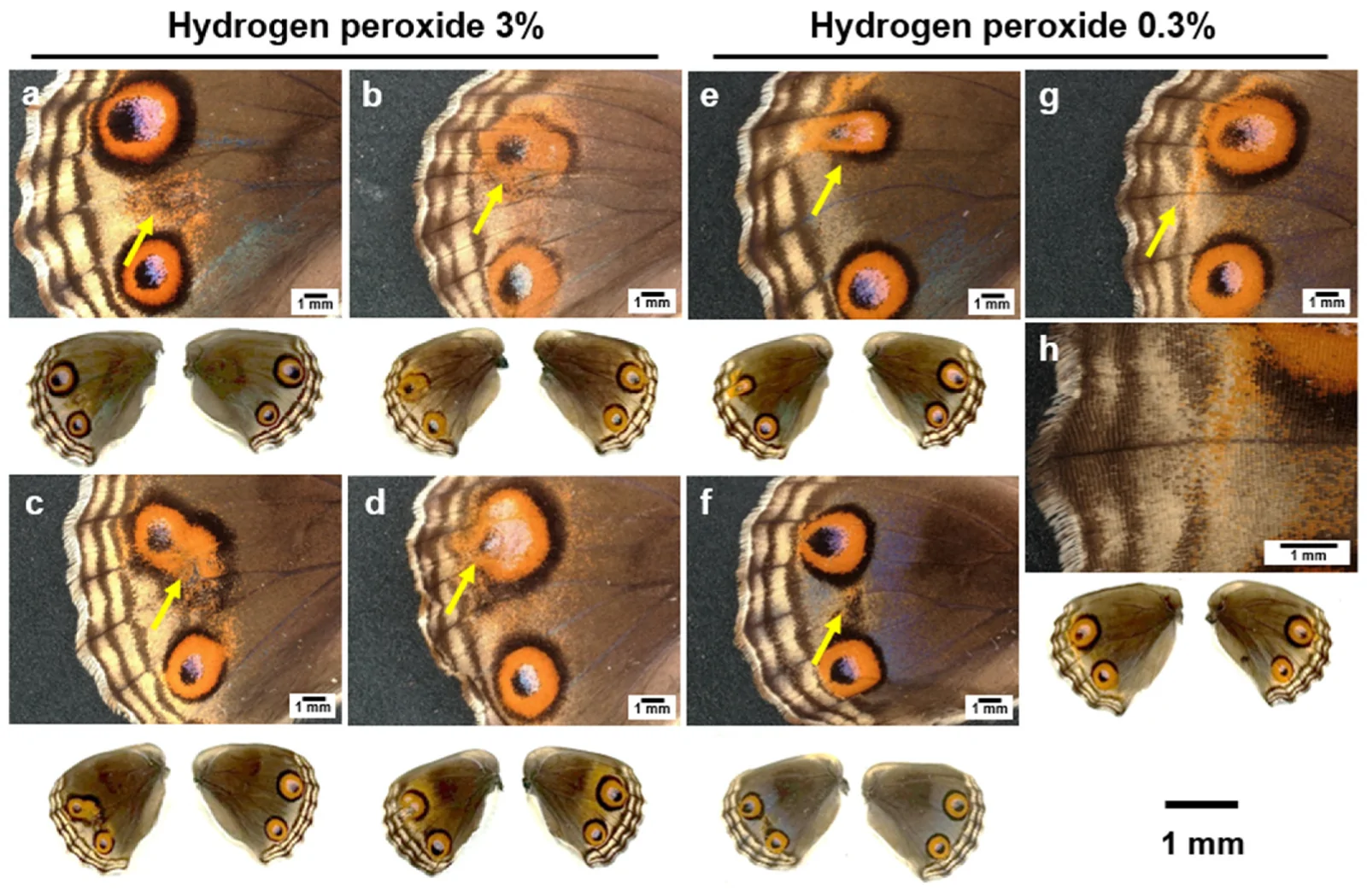
Dorsal hindwing color pattern modifications induced by topical applications of hydrogen peroxide (3% and 0.3%) in females. Overall wing color patterns of both left (treated) and right (untreated) wings are shown below the magnified images. Arrows indicate modifications. (**a**) Extra orange and black scales. (**b**) Eyespot deformation. (**c**) Eyespot size reduction with deformation. (**d**) Eyespot deformation. (**e**) Eyespot size reduction with deformation. (**f**) Extra black scales. (**g**) Disappearance of a part of PFE. (**h**) High-magnification image of the PFE area shown in (**g**).

In females, when 6% hydrogen peroxide was applied, 18 individuals eclosed successfully, and among them, we obtained various color pattern changes, including the emergence of extra orange scales (*n* = 4) (Figure 7a), deformation of eyespot circular patterns (*n* = 5) (Figure 7b), reduction in eyespot size (*n* = 8) (Figure 7c), enlargement of eyespot size (*n* = 2) (Figure 7d), and minor inward extension of PFE (*n* = 2) (Figure 7e–h). Interestingly, in four individuals that had structural blue scales on the right hindwing, the treated left wings showed fewer structural blue scales than the untreated right wings (Figure 7c,d,g). We further performed the same experiments using lower concentrations of hydrogen peroxide (3% and 0.3%) and obtained similar results (Appendix A, Figure A4).

**Figure 8:**
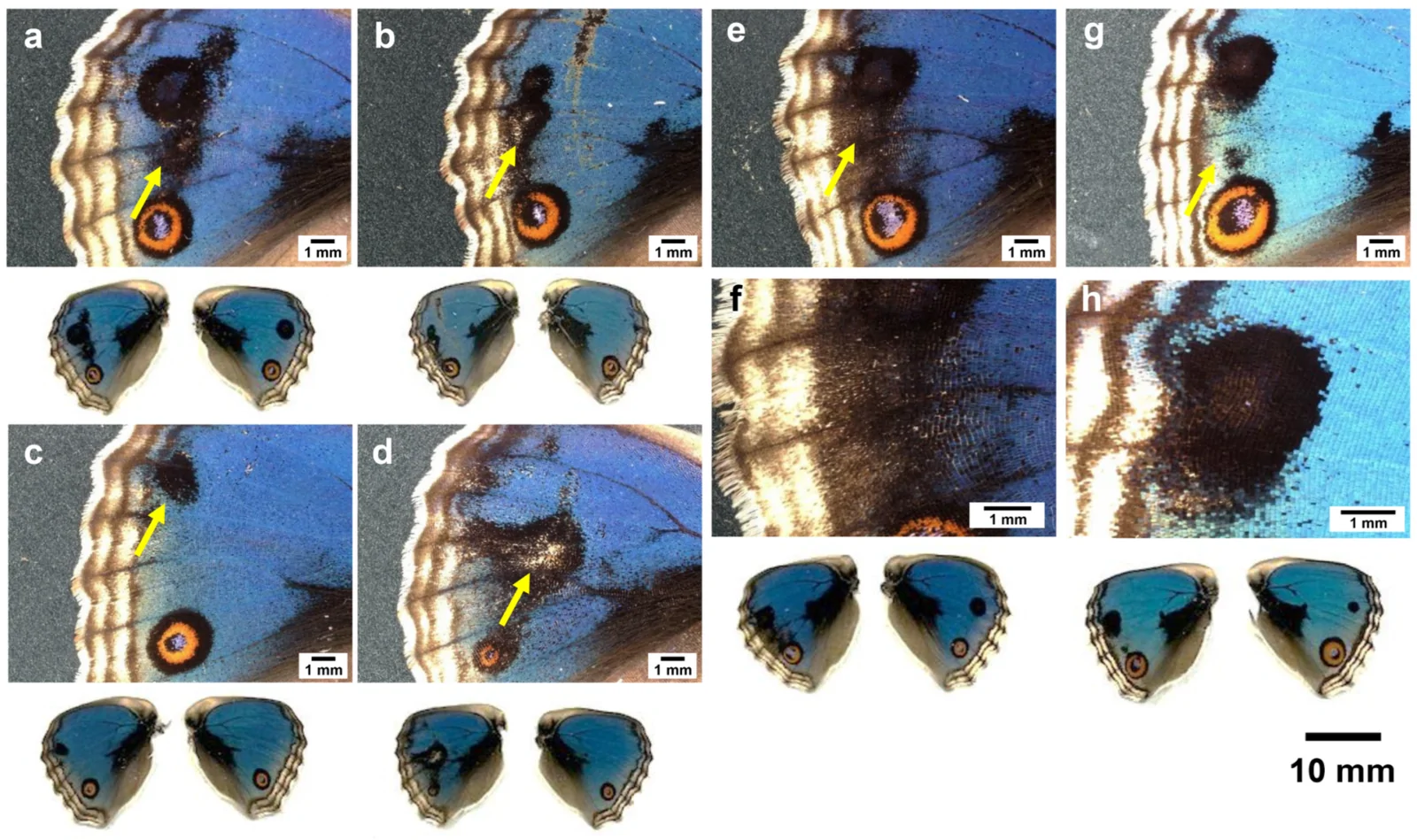
Dorsal hindwing color pattern modifications induced by topical application of hydrogen peroxide (6%) in males. Overall wing color patterns of both the left (treated) and right (untreated) wings are shown below the magnified images. The arrows indicate the modifications. (**a**–**c**) Emergence of black spots. (**d**) Emergence of black spot (with wing damage) and eyespot size reduction. (**e**,**g**) Inward extension of the PFE. (**f**,**h**) High-magnification image of (**e**,**g**).

**Figure A5:**
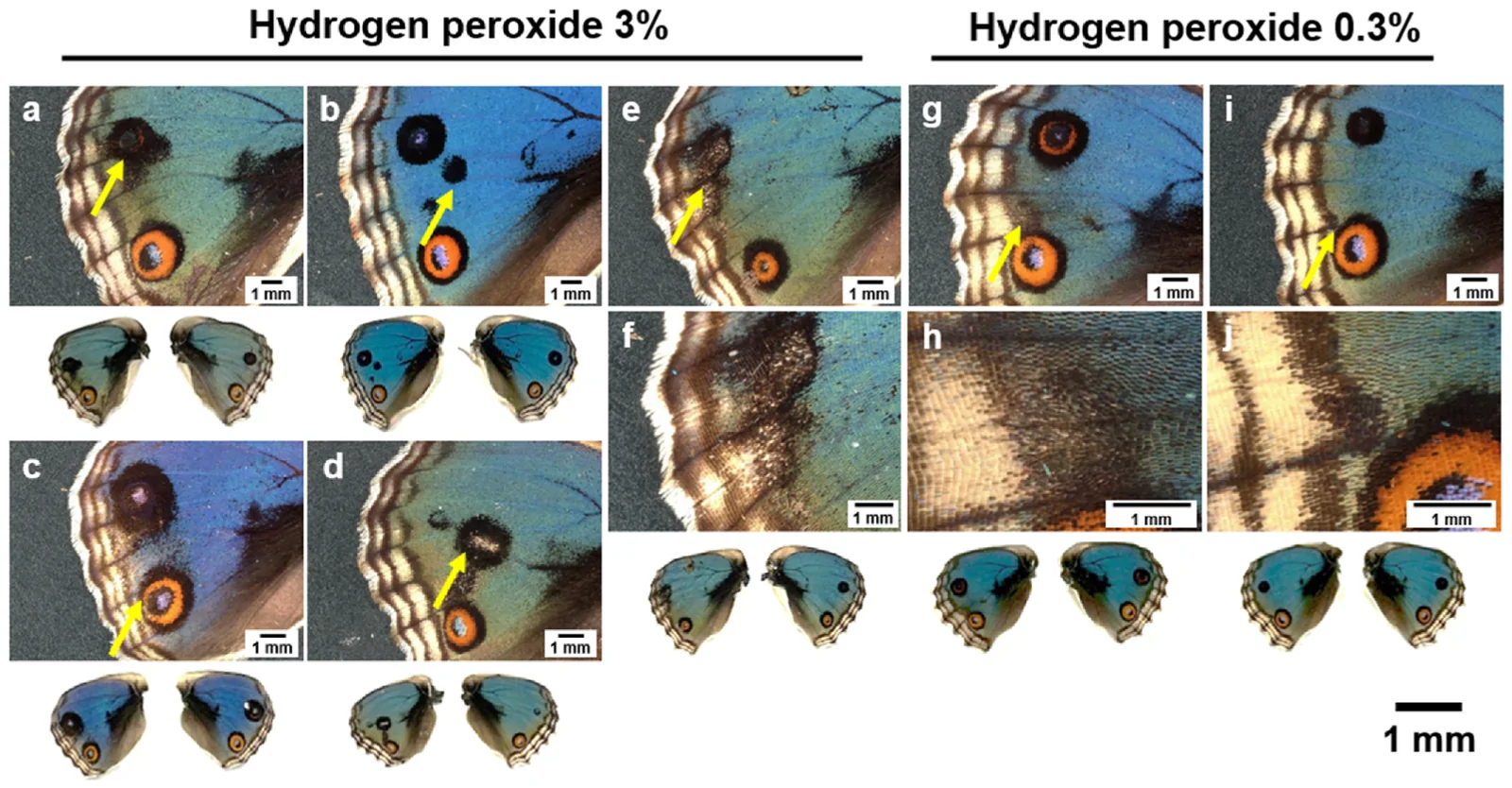
Dorsal hindwing color pattern modifications induced by topical application of hydrogen peroxide (3% and 0.3%) in males. The overall wing color patterns of both the left (treated) and right (untreated) wings are shown below the magnified images. Arrows indicate modifications. (**a**) Eyespot deformation. (**b**) Extra black spots. (**c**) Expansion of the outer black ring. (**d**) Black spot with damage. (**e**,**g**,**i**) Inward extension of PFE. (**f**,**h**,**j**) High-magnification image of the PFE area shown in (**e**,**g**,**i**).

In males, 36 individuals eclosed successfully after treatment with 6% hydrogen peroxide, and among them, we obtained various color pattern individuals with extra black scale emergence (*n* = 33) (Figure 8a), reduction in eyespot size (*n* = 2), emergence of anterior eyespot (*n* = 2) (Figure 8b,c), wing damage with black scale emergence (*n* = 2) (Figure 8d), and inward extension of PFE with black scale emergence (*n* = 2) (Figure 8e–h). In lower concentrations of hydrogen peroxide (3% and 0.3%), we obtained similar results (Appendix A, Figure A5).

In summary, we found that hydrogen peroxide induced orange scales in females and black scales in males. Eyespot modifications varied, but eyespots were severely deformed in many treated individuals. PFE showed inward extension in some individuals.

### 3.7. Topical Applications of Hydrogen Peroxide: Qualitative Changes in the Ventral Forewing

**Figure 9:**
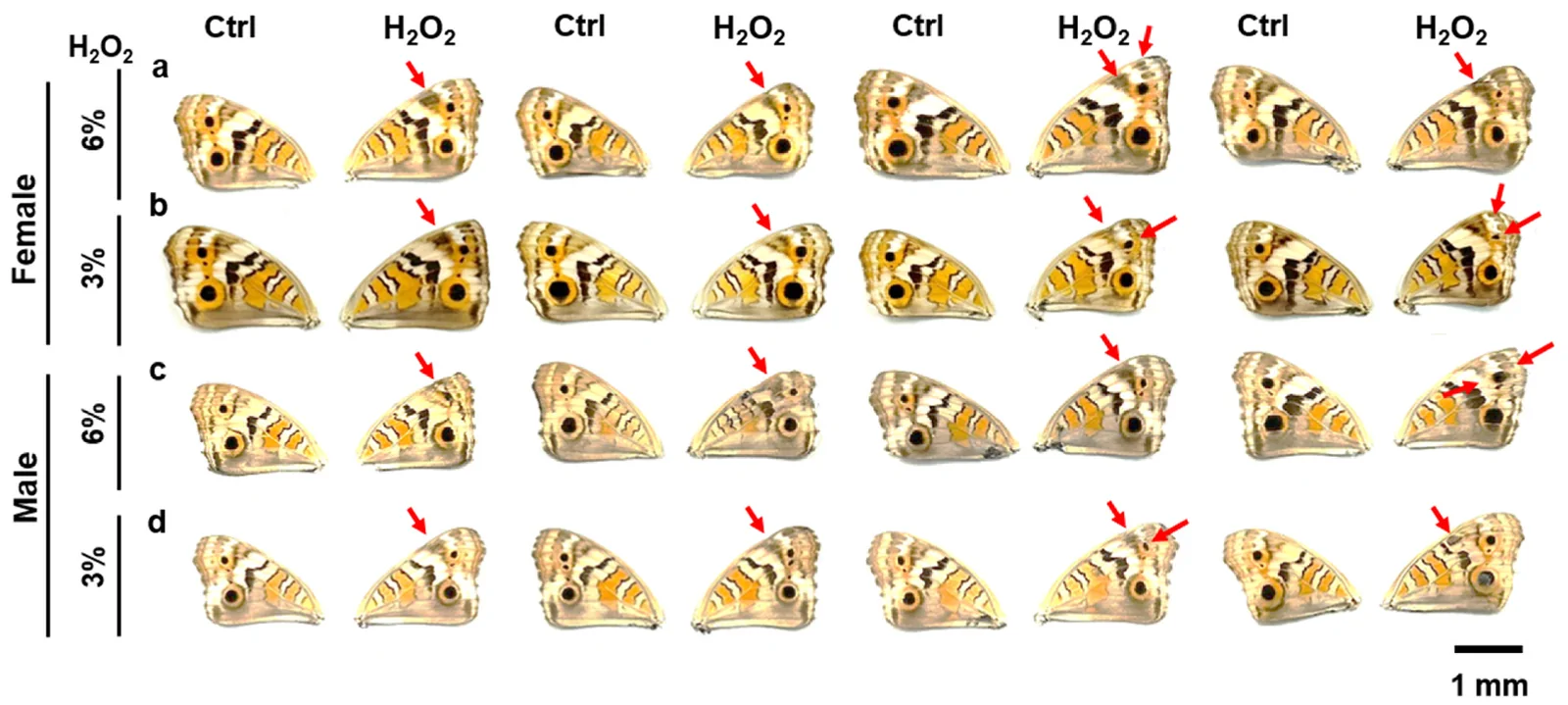
Color pattern modifications on the ventral side of the forewing after topical application of hydrogen peroxide. A pair of left (treated) and right (untreated) wings from the same individual are shown. Arrows indicate modified elements. (**a**) Hydrogen peroxide (6%) in females. (**b**) Hydrogen peroxide (3%) in females. (**c**) Hydrogen peroxide (6%) in males. (**d**) Hydrogen peroxide (3%) in males.

Here, we qualitatively examined the effects of topical applications of hydrogen peroxide on color pattern elements other than the eyespot on the ventral forewing. We focused on the anterior region of the ventral forewing, which could have made contact with hydrogen peroxide during the sandwich treatment. Compared with the untreated right wing, the treated left wing showed minor changes around the anterior eyespot region. In comparison with the untreated right wings (*n* = 142), the treated left wings (*n* = 142) showed diffusively elongated black bands (ABB and BBB) on the proximal side in both sexes and at both 6% and 3% (but not at 0.3%) concentrations of hydrogen peroxide (*n* = 52) (Figure 9), which, in some individuals, was accompanied by a reduction in the size of the anterior eyespots (*n* = 30) (Figure 9). These results were similar to those of the dorsal hindwings treated with hydrogen peroxide using the sandwich method.

### 3.8. Topical Applications of Catalase and DMTU: Eyespot Size

Here, we tested two hydrogen peroxide scavengers, catalase and DMTU, on the dorsal hindwing eyespots using the sandwich method. Because their effects on the eyespot did not accompany any eyespot deformations, we were able to quantify the eyespot size for quantitative comparison.

**Figure 10:**
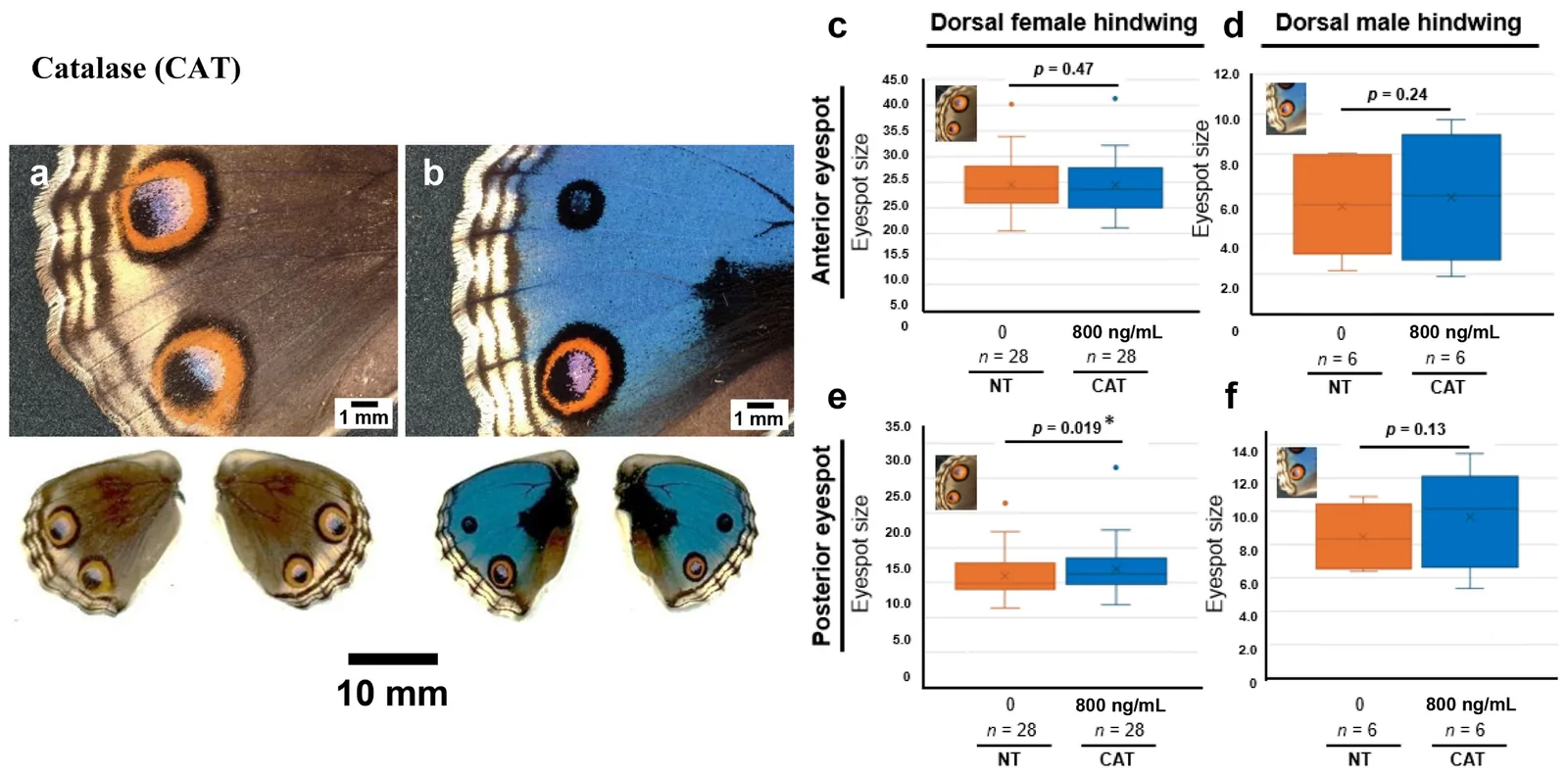
Topical applications of catalase (CAT). *: *p* < 0.05. Overall wing color patterns of both left (treated) and right (untreated) wings are shown below the magnified images. NT: no treatment. (**a**) Eyespot expansion in a female individual. (**b**) Eyespot expansion in a male individual. (**c**) Hindwing anterior eyespot size in females. Inset image indicates the wing surface of interest in each panel (**c**–**f**). (**d**) Hindwing anterior eyespot size in males. (**e**) Hindwing posterior eyespot size in females. (**f**) Hindwing posterior eyespot size in males.

In the catalase group, there was no clear color pattern modification in either sex based on visual inspection (*n* = 28) (Figure 10a,b). Quantitatively, the female dorsal hindwing anterior eyespot (*n* = 28) (*p* = 0.47; *d* = 0.033) (Figure 10c) and the male dorsal hindwing anterior eyespot (*n* = 6) (*p* = 0.24; *d* = 0.15) (Figure 10d) did not show a significant difference compared with the contralateral (untreated) wing. Only the female dorsal hindwing posterior eyespot (*n* = 28) showed a significantly larger eyespot compared with the contralateral wing (*p* = 0.019; *d* = 0.26) (Figure 10e). The male dorsal hindwing posterior eyespot (*n* = 6) did not show a significant difference (*p* = 0.13; *d* = 0.47) (Figure 10f).

**Figure 11:**
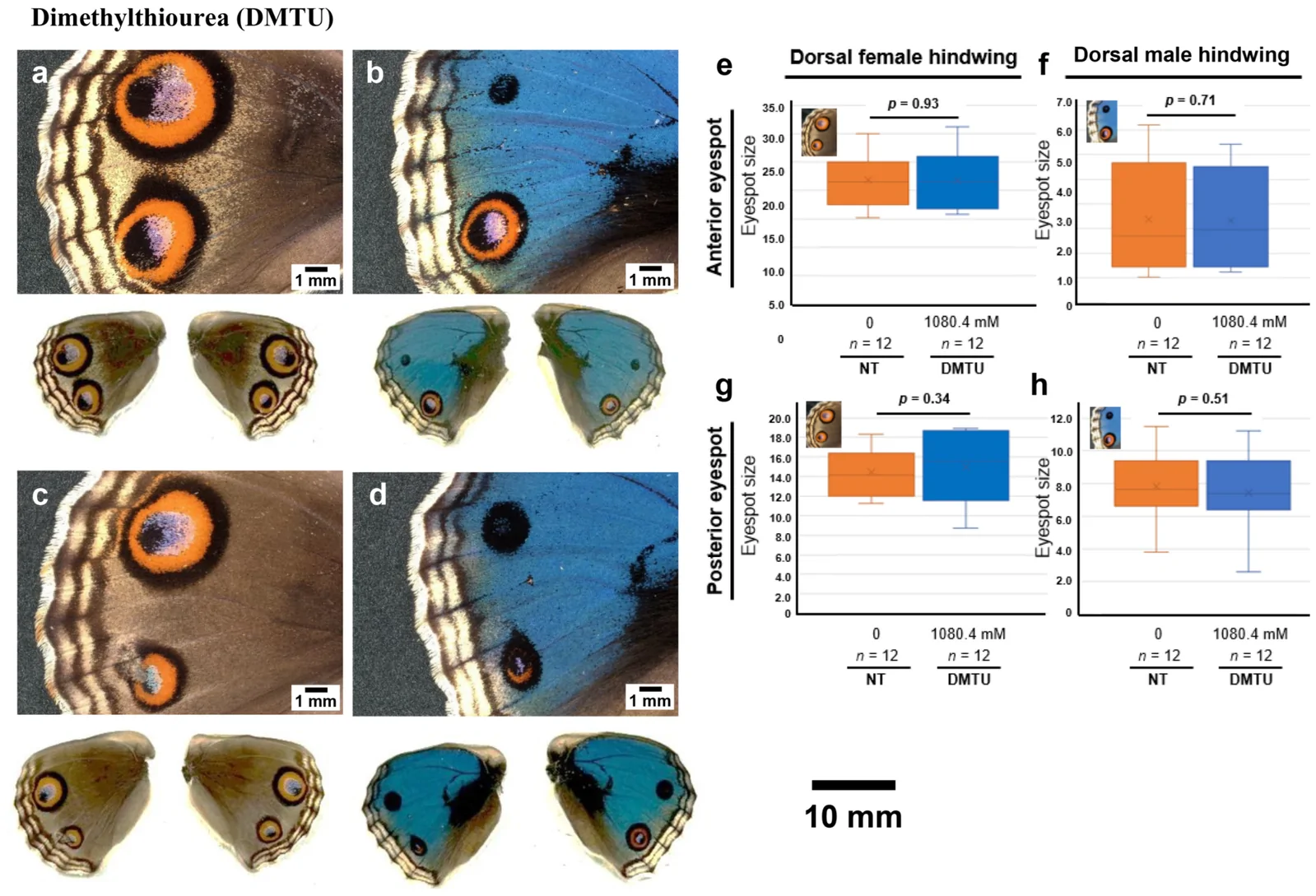
Topical application of DMTU. Overall wing color patterns of both the left (treated) and right (untreated) wings are shown below the magnified images. NT: no treatment. (**a**) Eyespot expansion in a female individual. (**b**) Eyespot expansion in a male individual. (**c**) Eyespot reduction in a female individual. (**d**) Eyespot reduction in a male individual. (**e**) Hindwing anterior eyespot size in females. The inset image indicates the wing surface of interest in each panel (**e**–**h**). (**f**) Hindwing anterior eyespot size in males. (**g**) Hindwing posterior eyespot size in females. (**h**) Hindwing posterior eyespot size in males.

The DMTU-treated wings showed either increased or reduced eyespots on visual inspection (Figure 11a–d). Probably because of this fluctuation among individuals, there was no significant statistical difference in eyespot size in the DMTU treatment, as follows: the female dorsal hindwing anterior eyespot (*p* = 0.93; *d* = 0.0074) (Figure 11e), the male dorsal hindwing anterior eyespot (*p* = 0.71; *d* = 0.16) (Figure 11f), the female dorsal hindwing posterior eyespot (*p* = 0.34; *d* = 0.031) (Figure 11g), and the male dorsal hindwing posterior eyespot (*p* = 0.51; *d* = 0.17) (Figure 11h).

### 3.9. Topical Applications of FB28

**Figure 12:**
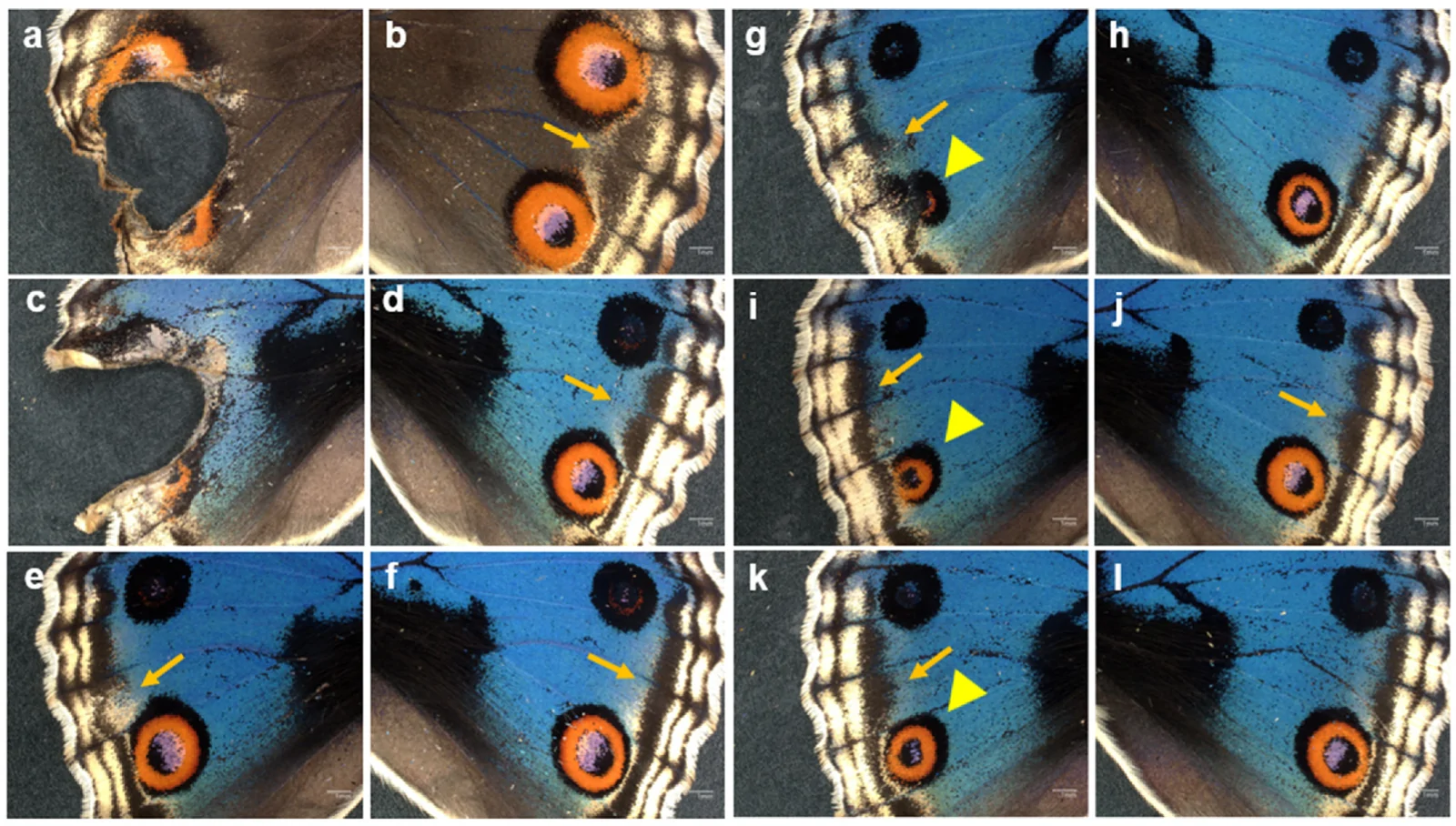
Dorsal hindwings treated with topical applications of FB28. A pair of left (treated) and right (untreated) wings from the same individual are shown. Arrows indicate modified PFEs, and arrowheads indicate eyespot size reduction. (**a**,**b**) A female individual with extensive elimination of wing area. (**c**,**d**) A male individual with extensive elimination of wing area. (**e**–**l**) Male individuals with color pattern modifications.

For comparison to the topical treatments with hydrogen peroxide, DMTU, and catalase, we performed topical applications of FB28. One female and six males successfully eclosed after treatment. Among them, two individuals showed a lack of a large wing area on the left wing where FB28 was sandwiched (Figure 12a–d). A part of the eyespot was eliminated, but the eyespot shape did not seem to be affected much (Figure 12a–d). The untreated right wing showed PFE extension to the proximal side in both female and male individuals (Figure 12b,d), indicating that FB28 was absorbed and circulated to the right wing in these individuals. On the dorsal hindwing treated with the sandwich method, two individuals showed disruption of PFE (Figure 12e,g), and three individuals showed eyespot size reduction compared with the right wing (Figure 12g–l).

Overall, FB28-induced modifications via topical application were qualitatively different from those induced by hydrogen peroxide. FB28-induced modifications via topical application were also different from those induced by DMTU and catalase, despite some similarities between DMTU and FB28 in systemic applications (Figure 5).

### 3.10. Mechanical Load Test for Cuticle Hardness

**Figure 13:**
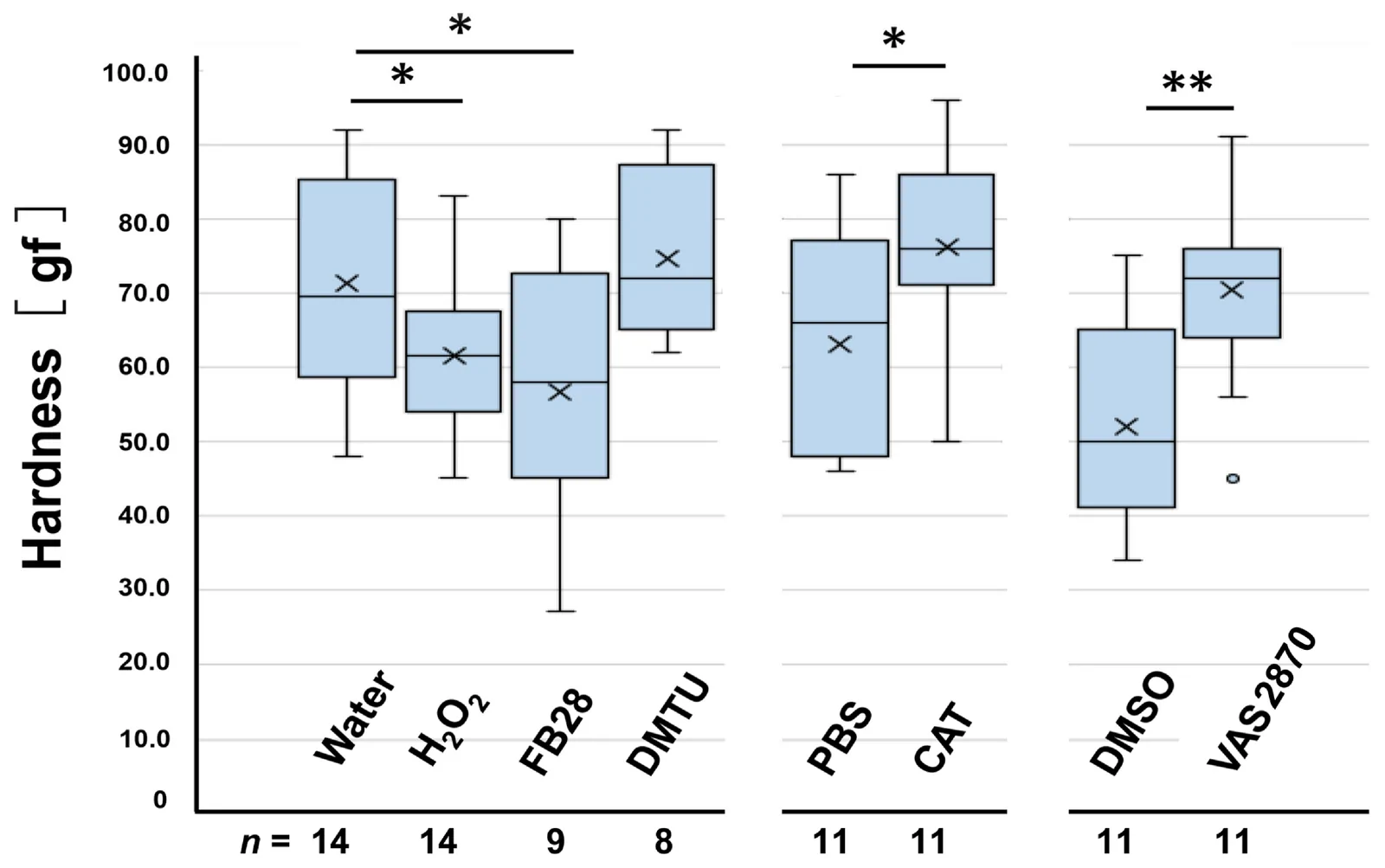
Mechanical load test to examine the relationship between cuticle hardness and chemical treatments. *: *p* < 0.05, **: *p* < 0.01. The water, PBS, and DMSO groups were used as solvent controls. Hardness is shown in gram force (gf). For Bonferroni corrections for multiple comparisons, *p*-values are to be tripled or doubled in the water-treatment group.

It is important to examine how hydrogen peroxide in wings induces color pattern modifications in butterfly wings. Based on the present results, we hypothesized that hydrogen peroxide affects cuticle hardness, similarly to FB28. Here, we conducted a mechanical load test to measure the cuticle hardness of chemically treated pupae (Figure 13).

We first measured the cuticle hardness of pupae treated with water because water was used as the solvent for hydrogen peroxide, FB28, and DMTU. Compared with the water-treatment group (*n* = 14), the H_2_O_2_-treatment group (*n* = 14) showed a significantly lower value of cuticle hardness (*p* = 0.036; *d* = 0.87). The FB28-treatment group (*n* = 9) also showed significantly lower cuticle hardness (*p* = 0.035; *d* = 1.0). The DMTU-treatment group (*n* = 8) did not show a significant difference (*p* = 0.57; *d* = 0.27).

Next, because catalase was dissolved in PBS, we measured the cuticle hardness of pupae treated with PBS. Compared with the PBS-treatment group (*n* = 11), the catalase-treatment group (*n* = 11) showed a significantly higher value of cuticle hardness (*p* = 0.031; *d* = 1.0).

In addition to these chemicals, we examined the effect of a NOX inhibitor, VAS2870, as a previous study showed the importance of NOX in color pattern formation [51]. Inhibition of NOX results in a decrease in hydrogen peroxide [51]. Because DMSO was the solvent for VAS2870, we measured the cuticle hardness of the pupae treated with DMSO. Compared with the DMSO-treatment group (*n* = 11), the VAS2870-treatment group (*n* = 11) showed a significantly higher value of cuticle hardness (*p* = 0.0041; *d* = 1.4).

## 4. Discussion

### 4.1. Systemic Applications of Hydrogen Peroxide, Catalase, DMTU, and FB28

**Figure 14:**
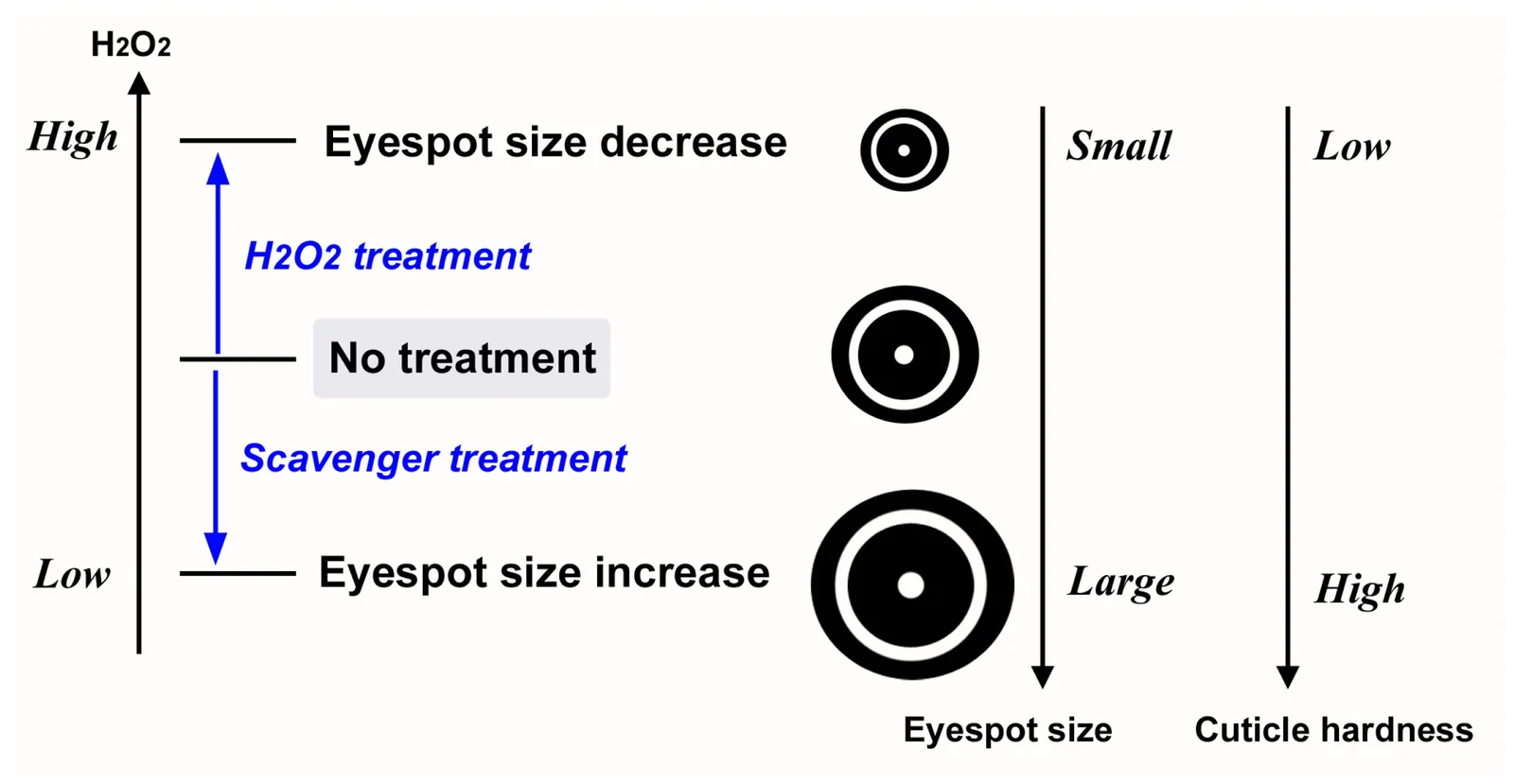
Summary of the major results of the present study. Pharmacological treatments are indicated in blue.

In this study, we tested the pharmacological effects of hydrogen peroxide and its scavengers, catalase and DMTU, by conducting systemic and topical applications. First, systemic application of hydrogen peroxide induced a reduction in eyespot size on the ventral forewing (Figure 2). Consistently, the ventral forewing eyespot was enlarged by systemic application of catalase (Figure 2). Similarly, the dorsal hindwing eyespot was enlarged by systemic application of DMTU (Figure 4). As a first approximation, hydrogen peroxide seems to regulate eyespot size (Figure 14). If eyespot size changes reflect physicochemical changes in the distribution of morphogenic signals, it seems that hydrogen peroxide suppresses the propagation of eyespot morphogenic signals at high concentrations.

In contrast, systemic applications of hydrogen peroxide enlarged the outer ring of the dorsal hindwing eyespots without changing the whole eyespot size. The effects of hydrogen peroxide may be more reflected on the outer ring than on the entire eyespot. This result is not perplexing, considering that morphogenic signals for the outer ring and the core disk may be released at different time points and may show semi-independent behaviors [32,77,78]. This might be a relatively late effect, because the expansion of the outer ring via hydrogen peroxide treatment is reminiscent of the fact that pupal focal damage at a relatively late (but not early) stage leads to the expansion of the outer ring [19]. Alternatively, endogenous hydrogen peroxide may fine-tune the outer ring at relatively low concentrations, which might have been disrupted by exogenous hydrogen peroxide. It is also important to note that the outer ring of the ventral hindwing eyespots was enlarged and disrupted by hydrogen peroxide in *J. almana* (Appendix A, Figure A3).

Throughout this pharmacological study, potential nonspecific effects of chemicals applied to pupae cannot be ruled out completely, as this is a methodological limitation inherent to the pharmacological approach. For example, at high concentrations of hydrogen peroxide, nonspecific oxidative stress and tissue damage may occur, which then could somehow induce a decrease in eyespot size. However, it is important to recognize that the eyespot size changes in response to the injection method do not seem to be a nonspecific effect, considering that the changes were observed consistently in treated individuals, among various chemicals used here, and in two different species. Importantly, the adult wings in the systemic application looked normal in morphology, which contrasts with the topical application. Furthermore, we obtained dose-dependent responses to hydrogen peroxide at low concentrations, although weak, and to DMTU over a relatively wide concentration range. Similarly, there is the possibility that the eyespot enlarging effect of DMTU may be a nonspecific effect [79]. However, we also used catalase, and the results were consistent, suggesting that the eyespot enlarging effect was executed through their scavenging activities. Therefore, we believe that the potential nonspecific effects of hydrogen peroxide, DMTU, and catalase are minimal in systemic applications in this study.

DMTU also induced color pattern modifications in non-eyespot elements, which were similar to those induced by FB28 (Figure 5). If this phenotypic similarity reflects similar molecular mechanisms, DMTU may induce color pattern modifications by decreasing the level of hydrogen peroxide that affects cuticle formation, because FB28 is a known cuticle binder. This suggests that the target of endogenous hydrogen peroxide may also be the cuticle.

As in the case of the effects on eyespots, clear modifications of non-eyespot elements by DMTU contrast with the results of hydrogen peroxide; only minor modifications of PFE were observed in an individual treated with hydrogen peroxide. Although minor, this PFE modification in the hydrogen peroxide group, similar to those in the DMTU group, is perplexing, because these two treatments should yield opposite results at least theoretically. However, the treatment with hydrogen peroxide might have simply disrupted PFE formation, as in the case of the treatment with DMTU. It is important to mention that we confirmed the effects of hydrogen peroxide for reducing eyespot and DMTU for enlarging eyespots in another species, *J. almana* (Appendix A, Figure A2).

### 4.2. Topical Applications of Hydrogen Peroxide, Catalase, DMTU, and FB28

We also performed topical applications of hydrogen peroxide. Hydrogen peroxide on the surface of the dorsal hindwing deformed, reduced, and enlarged eyespots in both females (Figure 7) and males (Figure 8). Such variation could be understood as a result of disrupting endogenous hydrogen peroxide for color pattern formation. However, high concentrations of exogenous hydrogen peroxide might have chemically damaged epidermal cells directly. Orange and black scales appeared frequently on the background area in females and males, respectively. These ectopic scales are similar to the modifications induced by physical damage at the background area in this species [80]. In fact, it may be difficult to clearly distinguish the eyespot changes via signaling modifications from the damage-induced changes. Topical applications also induced color pattern changes on the ventral forewing in both sexes (Figure 9). These variable and inconsistent results of the topical application are largely in contrast to those of the injection method, and variable modifications may largely be made due to nonspecific oxidative stress and tissue damage, especially at high concentrations.

In addition, the results of the topical applications of catalase (Figure 10) and DMTU (Figure 11) were not remarkable. The applications induced eyespot size variations on the dorsal hindwing, as in the case of hydrogen peroxide, but collectively, these changes were not statistically significant except for catalase (800 ng/mL) on the female posterior eyespot. The potential difficulty in inducing modifications by topical applications of scavengers may be understood in that they may stay on the cuticle surface of the wings and that hydrogen peroxide, working for color patterns, may be located differently in space, for example, inside or below the cuticle layer.

The topical application of FB28 also yielded very different results; eyespot reduction and thickened PFE (i.e., TS-type modifications) were clearly observed (Figure 12) as expected from the previous results [53]. However, the eyespot disruption by FB28 was very different from that induced by hydrogen peroxide, suggesting a unique function of endogenous hydrogen peroxide in vivo. Interestingly, high levels of FB28 appeared to be toxic to epidermal cells, resulting in the elimination of a large area of the hindwing (Figure 12a,c). Because FB28 is known as a cuticle inhibitor, proper cuticle formation is likely vital for epidermal cells. Proper cuticle formation is likely required for epidermal cells to survive and for the propagation of morphogenic signals, as suggested in previous studies [52,81]. However, the eyespots were not highly disrupted in shape by FB28 despite severe wing area elimination in the present study (Figure 12a,c). Epidermal death might have been induced later after the eyespot structures were determined. Chemical elimination of a large wing area is also known in the case of NOX inhibitors (VAS2870, isuzinaxib, and diphenyleneiodonium chloride (DPI)) [51]. In the case of NOX inhibitors, the eyespot structures were severely deformed together with the wing shape, which is in high contrast to the case of the FB28 treatment.

### 4.3. Scavengers Versus NOX Inhibitors

As mentioned above, the wings treated with one of the NOX inhibitors via topical application were severely deformed [51]. This is probably due to the lack of hydrogen peroxide on the wing margin [51]. The present study on scavengers of hydrogen peroxide (catalase and DMTU) did not show any effect on wing morphology. The scavengers may work only acutely and are not powerful enough to induce apoptosis in epidermal cells. In contrast, NOX inhibitors may last for a relatively long time and induce apoptosis in epidermal cells.

Our previous study showed that NOX inhibition results in sex-dependent outcomes: eyespot size reduction in females and eyespot size expansion in males [51]. In the present study, we did not observe clear sex-dependent differences; both sexes largely responded in a similar manner, although sensitivity to DMTU seemed to differ between sexes. To obtain sex-dependent results, long-term inhibition of hydrogen peroxide may be necessary. As discussed above, the inhibition of NOX may decrease hydrogen peroxide levels gradually for a relatively long time, but scavengers such as catalase and DMTU may decrease hydrogen peroxide levels immediately for a relatively short time.

### 4.4. Effect on the Wing Veins

In the present study, we observed wavy wing veins in response to systemic applications of hydrogen peroxide. A possible explanation for this result is that cuticle formation of the veins was impaired by hydrogen peroxide. In other words, for the wing veins to run straight, proper cuticle hardness is required. Because color pattern changes were observed in most individuals without wavy veins, the effects on color patterns and veins were probably independent of each other. However, the underlying mechanism would be similar: proper cuticle hardness is required for the development of color patterns and wing veins.

In contrast, wavy veins have also been found in individuals treated with anti-TRPA1 antibody in a recent study [58]. In that study, it was suggested that vein deformation might have affected color pattern modifications in the treated individuals [58] based on the fact that color patterns are severely disrupted in spontaneous wing vein mutants [82]. In addition, it has been shown that activation of TRPA1 by an agonist (JT010) induces eyespot size reduction [58]. Hydrogen peroxide is known to activate TRPA1, and hydrogen peroxide can reduce eyespot size, as shown in the present study. Thus, there is the possibility that hydrogen peroxide exerts its effect on eyespots through TRPA1 as well as through the cuticle. Activation of TRPA1 by ROS may be widely seen in many animals [83,84]. Another mechanosensory receptor, PIEZO1, has been shown to be involved in color pattern formation in butterflies [57]. Wing veins and eyespots share the expression of common genes during development [25].

### 4.5. Cuticle Hardness: Adjusting Covalent Bonds for Positional Information

The mechanical load test suggested that eyespot size was related to cuticle hardness (Figure 14). It is likely that the high hydrogen peroxide level via systemic applications of hydrogen peroxide resulted in low cuticle hardness and small eyespots (Figure 14). In contrast, the low hydrogen peroxide level via systemic applications of scavengers resulted in high cuticle hardness and large eyespots. We also tested FB28, a cuticle inhibitor that disrupts chitin–protein interactions, and demonstrated that FB28 induced low cuticle hardness and small eyespots in this study, confirming the previous results [53]. These results suggest that hydrogen peroxide directly regulates cuticle hardness (i.e., sclerotization) by regulating the chemical crosslinking of cuticular proteins or chitin–protein interactions. These results are consistent with previous studies, suggesting that cuticle hardness determines eyespot size [53] and that physicochemical properties of the materials facing the wing epidermis determine eyespot size [81].

Hydrogen peroxide degrades polysaccharides, such as chitin, at high concentrations, with or without tungstate [85,86,87]. Indeed, tungstate was identified as the first color pattern modification inducer [56,88]. Reactive oxygen species (ROS), including hydrogen peroxide at low concentrations, may be recruited to adjust the crosslinking of cuticle components in insects [89,90,91,92]. Cuticle sclerotization of the insect cuticle typically depends on insect-specific laccases or multicopper oxidases [93,94,95], and hydrogen peroxide is required for dityrosine bond formation as a distinct, highly resilient form of protein crosslinking [96,97,98]. Therefore, the effects of hydrogen peroxide on polysaccharides and cuticle proteins are crucial for understanding sclerotization and the development of insect cuticles and morphogenesis.

According to the physical distortion hypothesis, mechanical push and pull among epidermal cells in pupal wing tissues function as mechanical signals [99], and we have previously shown that intimate contacts between epidermal cells and cuticle may be important in determining eyespot patterns and that cuticle is not static but dynamic [52]. Hydrogen peroxide probably regulates the level of cuticle crosslinking to adjust the transduction of mechanical forces in the dynamic movement of the pupal cuticle layer. Although the hindwing surface does not touch the pupal cuticle layer covering the dorsal side of the forewing, the hindwing surface is also covered with a thin cuticle layer [55]. Surface rigidity may also be provided by the ventral forewing. Therefore, the mechanisms of color pattern determination would be the same between the forewings and hindwings.

We also tested VAS2870, one of the NOX inhibitors, the effects of which on eyespots were tested in a previous study [51]. As expected, VAS2870 made the pupal cuticle harder (Figure 12). This result can be explained by the lowered levels of hydrogen peroxide produced by NOX. Interestingly, DMSO lowered the cuticle hardness in comparison to water. Based on the chemical characteristics of DMSO (i.e., high penetrance to cells and high solvent effect), the inhibition or degradation of covalent bonds in the cuticle may be expected. However, DMSO may behave as a ROS scavenger, and it has been reported that systemic applications of DMSO result in eyespot enlargement (instead of reduction) in females but not in males [51]. The mechanisms of DMSO on eyespots should be investigated in the future.

### 4.6. Possible Signal Transduction Pathway

**Figure 15:**
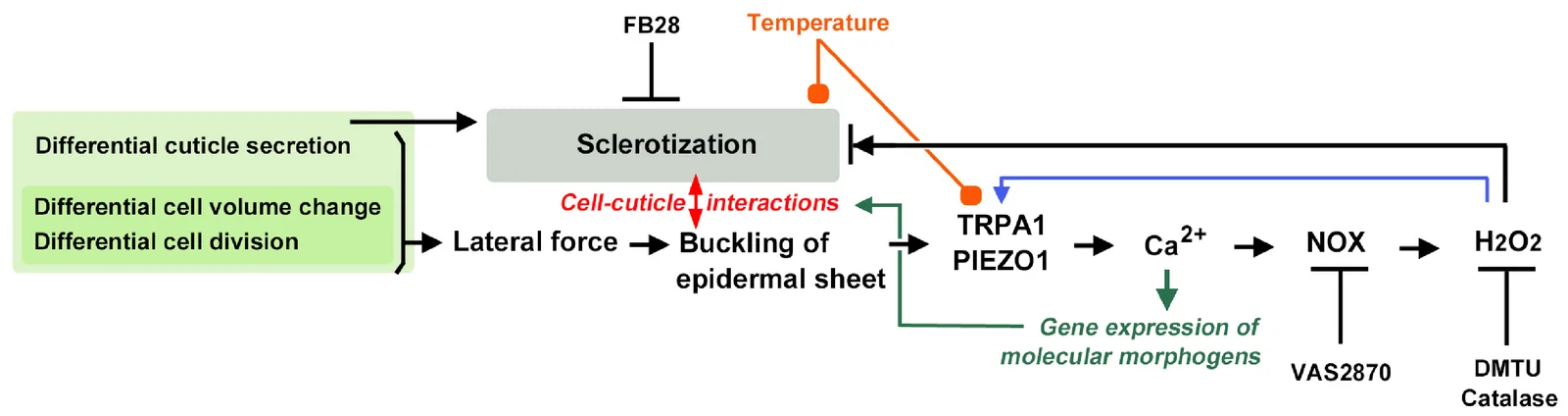
Possible signal transduction pathway in butterfly wing color pattern determination. This pathway is mechanically initiated to activate epidermal cells, and color pattern positional information is stored in the degree of sclerotization, which is decoded by epidermal cells through cell–cuticle interactions. In this way, both extracellular and intracellular events are involved. Cell–cuticle interactions may be regulated by molecular morphogens that are induced by calcium signals. Hydrogen peroxide activates or inhibits sclerotization depending on its concentration. Hydrogen peroxide also acts on TRPA1. The targets of the inhibitors (DMTU, catalase, VAS2870, and FB28) are also shown. Note that these steps in this figure have not been fully tested yet.

On the basis of the present and previous studies, we propose a possible signal transduction pathway for color pattern determination in butterfly wings (Figure 15). This model has not been fully supported by experiments yet, but it is important to overview a possible transduction pathway and to understand the possible status of hydrogen peroxide in that pathway. Lateral force is generated by differential cell volume change, differential cell division, and differential cuticle secretion. An increase in cell volume and cell division in organizing cells has been suggested [99,100,101,102], and high levels of cuticle secretion in organizing cells have also been shown in TEM images [52,55]. The lateral force causes the outward buckling of the epidermal sheet on the dorsal forewing. The physical distortions produced by the buckling open mechanosensory cation channels such as PIEZO1 [57] and TRPA1 [58], resulting in calcium ion entry into cells [34,35]. Calcium ions then activate NOX to produce hydrogen peroxide [51]. Hydrogen peroxide then acts on the cuticle to increase or decrease the number of covalent bonds for sclerotization. In addition, hydrogen peroxide may act on TRPA1 to ensure the production of hydrogen peroxide at a proper level. TRPA1 may respond to temperature directly, which may partly explain the temperature-dependent phenotypic plasticity in butterflies.

One unique point in this model of signal transduction is that positional information for color expression is “stored” once in the spatial distribution of cuticle hardness in response to hydrogen peroxide, induced by physical distortions. Furthermore, such physical information (or chemical crosslinking information) is decoded by cellular interactions with the opposing cuticle through cell surface receptors such as integrins [102]. Because the cuticle sheet functions as a storage of positional information in this model, it is also called the cuticle hypothesis [52,55]. Gene expression of molecular morphogens is induced by calcium entry into cells, and the expressed morphogens may contribute to cell–cuticle interactions in this model [99,102].

Is hydrogen peroxide considered a morphogen? It is certainly different from conventional molecular morphogens, which are extracellular proteins that are supposed to form a gradient. On the other hand, functionally, hydrogen peroxide may be more suitable for forming a gradient than the conventional morphogens, as hydrogen peroxide spreads quickly over hundreds of cells within a limited time span. This is because hydrogen peroxide is a small and highly diffusible molecule. Moreover, sinks of hydrogen peroxide may be made realistically by positioning biological scavengers to establish a stable gradient. Having said that, higher concentrations of hydrogen peroxide reduced the eyespot size in the present study (Figure 14), and in the model discussed above, eyespot positional information is provided as a form of extracellular matrix but not by hydrogen peroxide itself. Therefore, it may not be appropriate to call hydrogen peroxide a morphogen, but it is likely a negative regulator of eyespot positional information.

It is worth mentioning that temperature-induced modifications in butterflies have been studied since the nineteenth century by Merrifield, Standfuss, and other early lepidopterists, which inspired Morgan (1907) [103] and Goldschmidt (1938) [104] for the interacting mechanisms between genes and environmental factors. This line of research has been succeeded by Nijhout (1984) [15,105]. The chemical induction of the temperature-induced phenotype has been achieved in the systemic application of tungstate by Otaki (1998) [56,106]. Further modification inducers, sulfated polysaccharides, have been discovered by Serfas and Carroll (2005) [107]. Partly because sulfated polysaccharides are known to interact with Wnt proteins [108,109,110,111], the systemic applications of sulfated polysaccharides have been widely used in various butterflies and moths [112,113,114,115]. At least some sulfated polysaccharides produce TS-type modifications indistinguishable from those induced by FB28 and tungstate [107], which are similar, although not identical, to those induced by DMTU (Figure 5). There is no doubt that Wnt proteins play an important role in color pattern formation in butterflies, likely through an interaction with ECM. In this sense, hydrogen peroxide may provide a proper ECM milieu with Wnt proteins for their function. For example, hydrogen peroxide may chemically modify sulfate group in ECM to which Wnt protein binds.

## 5. Conclusions

This study showed the effects of hydrogen peroxide on wing color pattern development in butterflies. The present results suggest that endogenous hydrogen peroxide functions as a regulatory molecule for color pattern development. A possible mechanism underlying this process is the modulation of cuticle hardness by adjusting the crosslinking of cuticle components, although the underlying structural and biochemical mechanisms remain to be elucidated. In this sense, positional information for prospective eyespots and other color pattern elements is temporarily stored in the cuticle layer as cuticle hardness. Considering that cuticle hardness is dependent on the number of chemical bonds in the cuticle, hydrogen peroxide may enhance covalent bonds at low concentrations and degrade covalent bonds at high concentrations. Other molecular targets of hydrogen peroxide may also be present, and one of them is TRPA1. Overall, the present study suggests that hydrogen peroxide regulates eyespot color patterns in butterfly wings mainly by adjusting covalent bonds in the cuticle. Visualization of hydrogen peroxide in pupal wing tissue in vivo is expected in future studies.

## Data Availability

The original contributions presented in this study are included in the article. Further inquiries can be directed to the corresponding author.

## Supplementary Materials

The following supporting information can be downloaded at https://www.mdpi.com/article/10.3390/insects17101032/s1: Figure S1: All wings of individuals that eclosed successfully (*J. orithya*) (pdf files); Figure S2: All wings of individuals that eclosed successfully (*J. almana*) (pdf files); Table S1: Eclosion rate for the injection method (excel file); Table S2: Eclosion rate for the sandwich method (excel file).

## Abbreviations

The following abbreviations are used in this manuscript:

ROS	Reactive oxygen species
ER	Eclosion rate
*J.*	*Junonia*
PFE	Parafocal element
CAT	Catalase
DMTU	Dimethylthiourea
FB28	Fluorescent brightener 28
NOX	NADPH oxidase
PBS	Phosphate-buffered saline
DMSO	Dimethyl sulfoxide
NT	No treatment
TRPA1	Transient receptor potential ankyrin 1
TS	Temperature shock

## Appendix A

### Appendix A.1. Water and PBS Negative Controls in J. orithya

To exclude the possibility that solvents may influence wing color pattern formation and eyespot size, we conducted systemic applications of deionized water and PBS and examined the eyespot size on the ventral forewing (Figure A1a–d) and on the dorsal hindwing (Figure A1e–g) in both sexes of *J. orithya*. In both water and PBS and in both sexes, we observed no significant differences between the treatment group and the no-treatment group as follows: the female ventral forewing anterior eyespot in water (*p* = 0.61; *d* = 0.19) (Figure A1a), the male ventral forewing anterior eyespot in water (*p* = 0.88; *d* = 0.056) (Figure A1b), the female ventral forewing anterior eyespot in PBS (*p* = 0.55; *d* = 0.24) (Figure A1c), the male ventral forewing anterior eyespot in PBS (*p* = 0.87; *d* = 0.056) (Figure A1d), the female dorsal hindwing anterior eyespot (*p* = 0.58; *d* = 0.21) (Figure A1e), the female dorsal hindwing posterior eyespot (*p* = 0.60; *d* = 0.19) (Figure A1f), and the male dorsal hindwing posterior eyespot (*p* = 0.44; *d* = 0.28) (Figure A1g).

### Appendix A.2. Hydrogen Peroxide and DMTU in J. almana: Quantitative Changes

To examine the effects of hydrogen peroxide not only in *J. orithya* but also in other species, we conducted systemic applications of hydrogen peroxide and DMTU in *J. almana* (Figure A2).

In the dorsal hindwing anterior eyespot, compared with the no-treatment group (*n* = 32), the hydrogen peroxide group (*n* = 22) did not show a significant difference (*p* = 0.71; *d* = 0.11), but the DMTU group (*n* = 25) significantly increased eyespot size (*p* = 0.0002; *d* = 1.2) (Figure A2a). In the dorsal hindwing posterior eyespot, compared with the no-treatment group (*n* = 32), the hydrogen peroxide group (*n* = 22) did not show a significant difference (*p* = 0.65; *d* = 0.13), but the DMTU group (*n* = 25) significantly increased eyespot size (*p* = 0.0023; *d* = 0.87) (Figure A2b). In the dorsal forewing anterior eyespot, compared with the no-treatment group (*n* = 32), the hydrogen peroxide group (*n* = 22) significantly reduced eyespot size (*p* = 0.014; *d* = 0.72), but the DMTU group (*n* = 25) did not show a significant difference (*p* = 0.092; *d* = 0.47) (Figure A2c). In the dorsal forewing posterior eyespot, compared with the no-treatment group (*n* = 32), the hydrogen peroxide group (*n* = 22) did not show a significant difference in eyespot size (*p* = 0.18; *d* = 0.39), but the DMTU group (*n* = 25) showed a significant enlargement of the eyespot (*p* = 1.68 × 10^−6^; *d* = 1.5) (Figure A2d).

In the ventral hindwing anterior eyespot, compared with the no-treatment group (*n* = 32), the hydrogen peroxide group (*n* = 19) significantly reduced eyespot size (*p* = 0.012; *d* = 0.77), and the DMTU group (*n* = 25) significantly increased eyespot size (*p* = 0.00080; *d* = 0.96) (Figure A2e). In the ventral hindwing posterior eyespot, compared with the no-treatment group (*n* = 32), the hydrogen peroxide group (*n* = 20) significantly reduced eyespot size (*p* = 0.00038; *d* = 1.1), and the DMTU group (*n* = 25) significantly increased eyespot size (*p* = 1.5 × 10^−6^; *d* = 1.5) (Figure A2f). In the ventral forewing anterior eyespot, compared with the no-treatment group (*n* = 32), the hydrogen peroxide group (*n* = 22) significantly reduced eyespot size (*p* < 0.0001; *d* = 1.6), but the DMTU group (*n* = 25) did not show a significant difference (*p* = 0.15; *d* = 0.40) (Figure A2g). In the ventral forewing posterior eyespot, compared with the no-treatment group (*n* = 32), the hydrogen peroxide group (*n* = 22) significantly reduced eyespot size (*p* = 0.00089; *d* = 1.0), and the DMTU group (*n* = 25) significantly increased eyespot size (*p* = 2.4 × 10^−6^; *d* = 1.4) (Figure A2h).

### Appendix A.3. Hydrogen Peroxide and DMTU in J. almana: Qualitative Changes

Here, we qualitatively examined the effects of injections of hydrogen peroxide and DMTU on the wing color patterns in *J. almana*. In the no-treatment group (*n* = 32), wing color patterns were invariant (Figure A3a,b). In the hydrogen peroxide (12%) group, 22 individuals eclosed successfully, and only one female individual showed outer ring blurring on the dorsal hindwing (Figure A3c), but three individuals showed the emergence of black scales and disruption of the outer ring on the ventral hindwing (Figure A3d). In the DMTU (432.16 mM) group, 25 individuals eclosed successfully, and there were no noticeable modifications on the dorsal and ventral wings (Figure A3e,f).

### Appendix A.4. Topical Applications of Hydrogen Peroxide (3% and 0.3%) in J. orithya

In addition to treatment with 6% hydrogen peroxide, we performed treatments with 3% and 0.3% hydrogen peroxide. In the treatment with 3% hydrogen peroxide, 18 females eclosed successfully, and among them we obtained individuals with extra orange scales (*n* = 9) (Figure A4a), deformation of eyespot patterns (*n* = 5) (Figure A4b,c), reduction in eyespot size (*n* = 2) (Figure A4c), and expansion of eyespot size (*n* = 1) (Figure A4d).

In 0.3% hydrogen peroxide, 13 individuals eclosed successfully, among which we obtained individuals with deformation of eyespot patterns (*n* = 1) (Figure A4e), reduction in eyespot size (*n* = 2) (Figure A4e), expansion of eyespot size (*n* = 1), extra black scales (Figure A4f) (*n* = 2), and the disappearance of a part of PFE (*n* = 1) (Figure A4g,h).

In males treated with 3% hydrogen peroxide, 36 individuals eclosed successfully, and among them, we obtained individuals with the deformation of the eyespot (*n* = 5) (Figure A5a), the emergence of extra black scales (*n* = 17) (Figure A5b), the expansion of outer black ring (*n* = 3) (Figure A5c), the wing damage with black scales (*n* = 3) (Figure A5d), the reduction in eyespot size (*n* = 1), and the minor inward extension of PFE (*n* = 2) (Figure A5e,f).

In 0.3% hydrogen peroxide, 21 individuals eclosed successfully, and among them, we obtained individuals with a minor inward extension of PFE (*n* = 2) (Figure A5g–j).
