# Supplementary material for: Hydrogen Peroxide Regulates Butterfly Wing Eyespot Color Patterns
Source: Insects. 2026 Oct 8;17(10):1032. doi: 10.3390/insects17101032 (PMC13705226; doi:10.3390/insects17101032)

Catalase 800 ng/ml Female

NO1

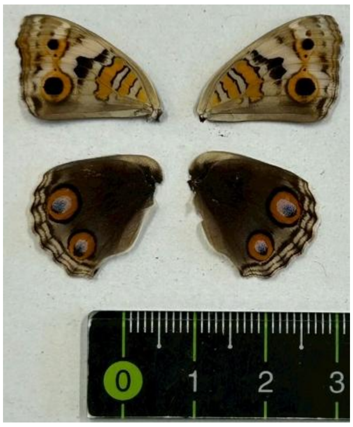


NO2

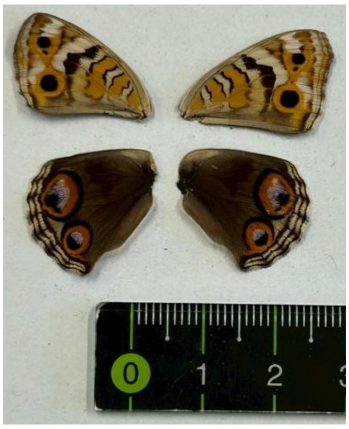


NO3

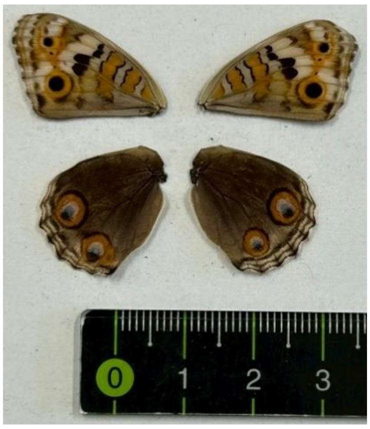


NO4

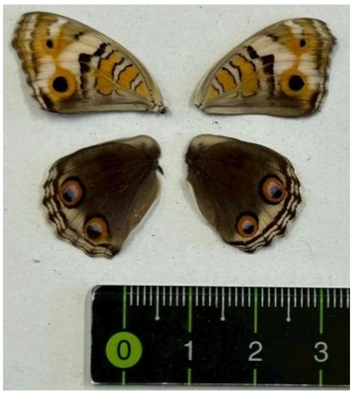


NO5

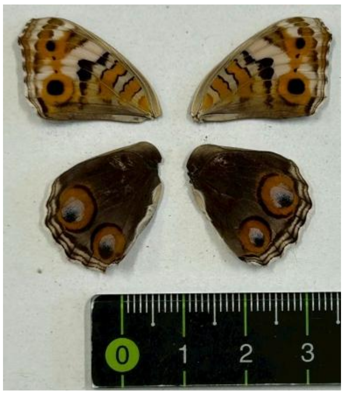


NO6

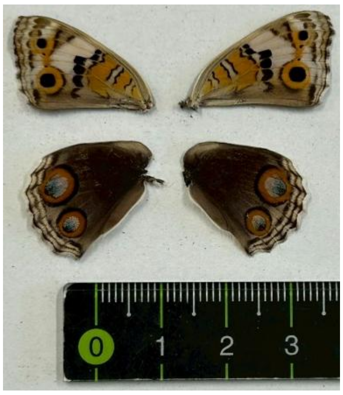


NO7

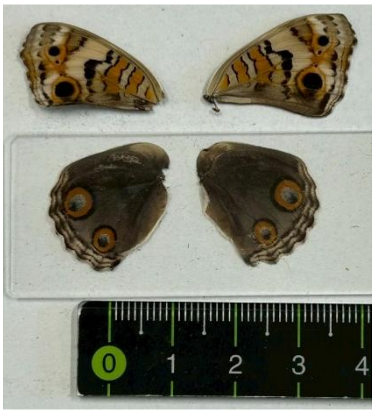


NO8

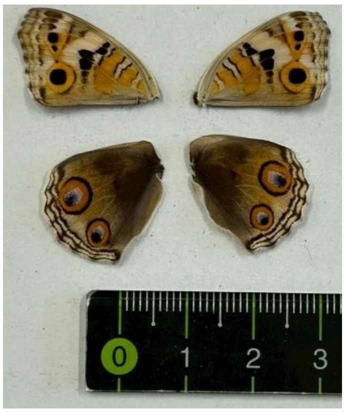


NO9

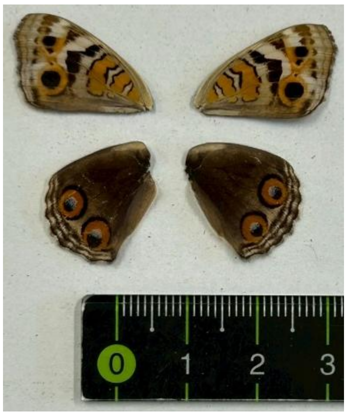

NO10

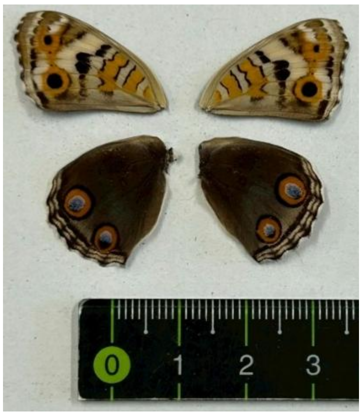


NO11

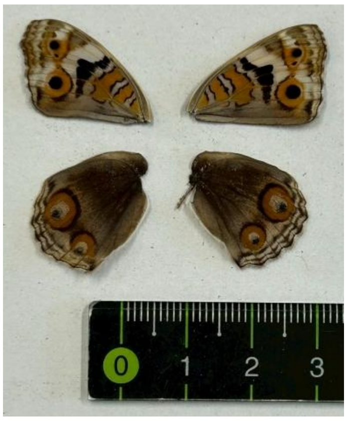


NO12

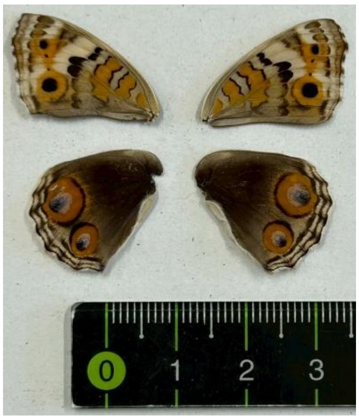


NO13

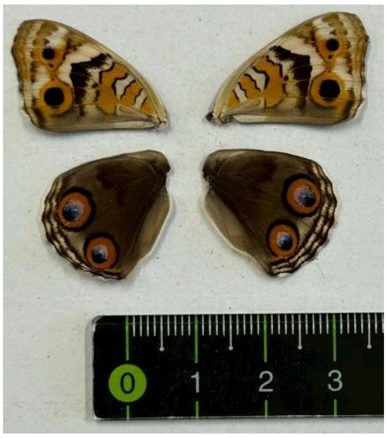


NO14

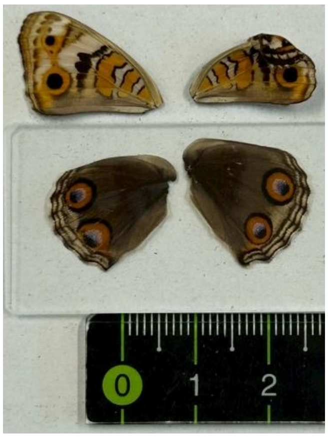


NO15

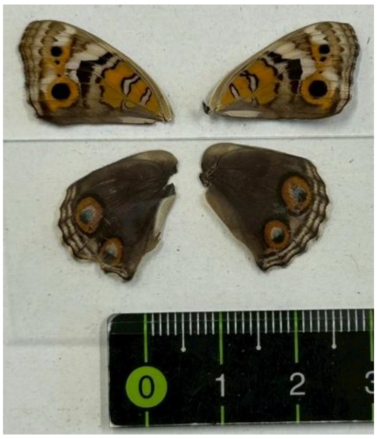


NO16

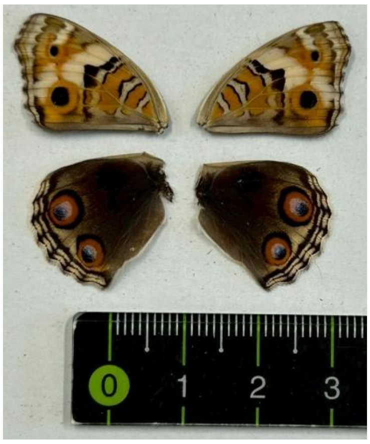


NO17

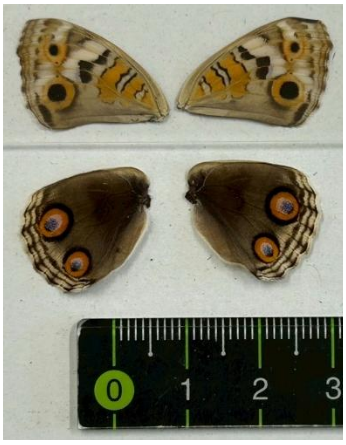


NO18

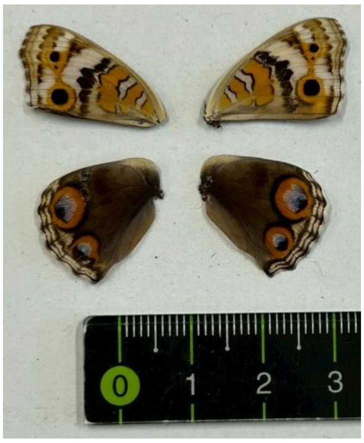

NO19

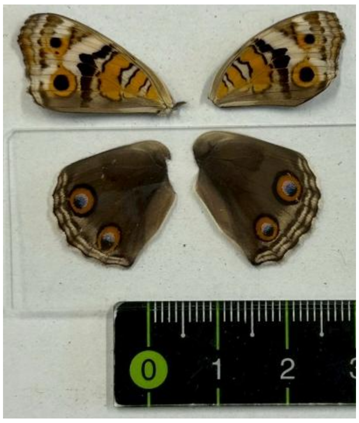

NO20

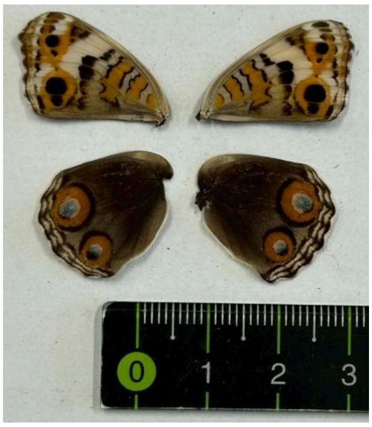

NO21

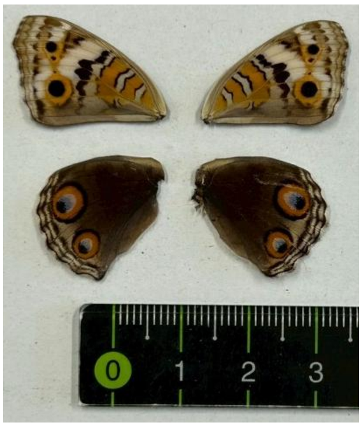

NO22

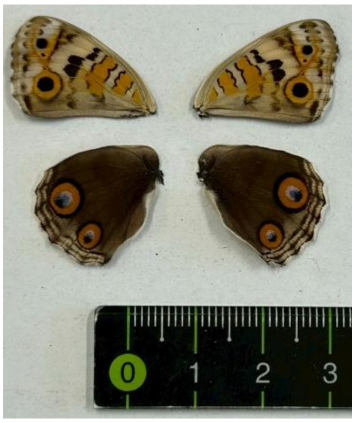

NO23

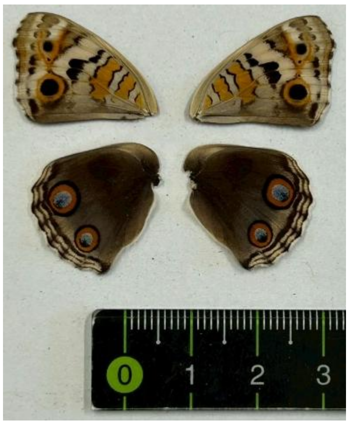

NO24

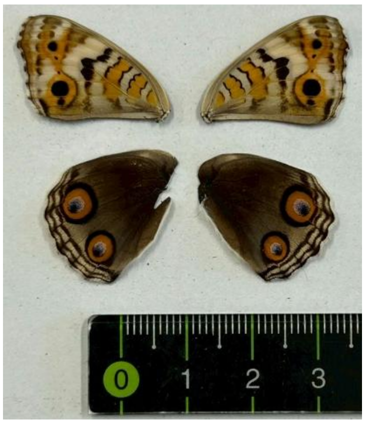

NO25

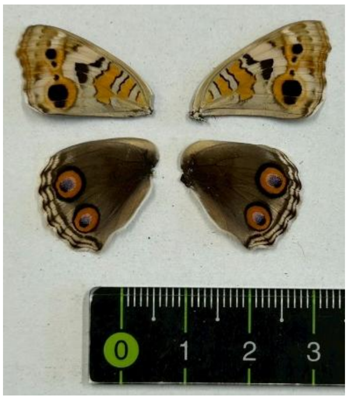

NO26

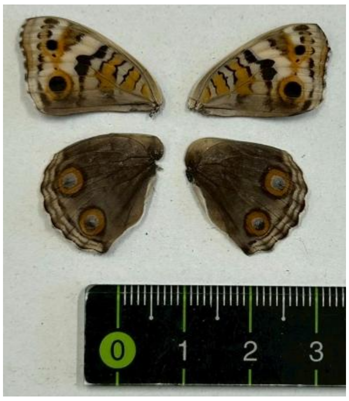

NO27

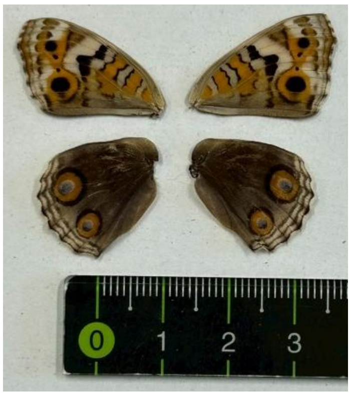

NO28

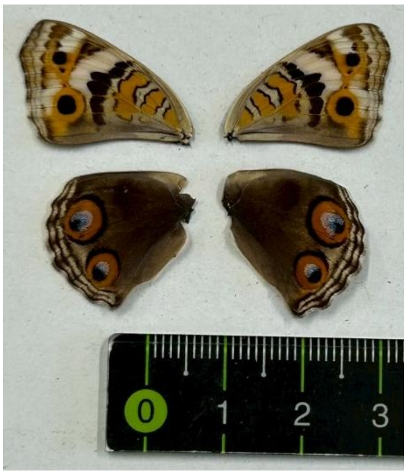

Supplement: Supplementary file 1 [file insects-17-01032-s001.zip › Supplementary Figures S1/Catalase/Sandwich/Catalase 800 ng sand female.pdf]
