# Supplementary material for: Hydrogen Peroxide Regulates Butterfly Wing Eyespot Color Patterns
Source: Insects. 2026 Oct 8;17(10):1032. doi: 10.3390/insects17101032 (PMC13705226; doi:10.3390/insects17101032)

Catalase 800 ng/ml Male

NO1

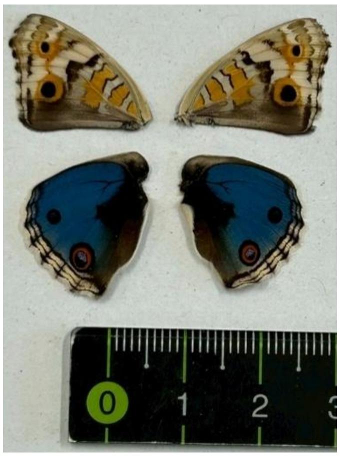

NO2

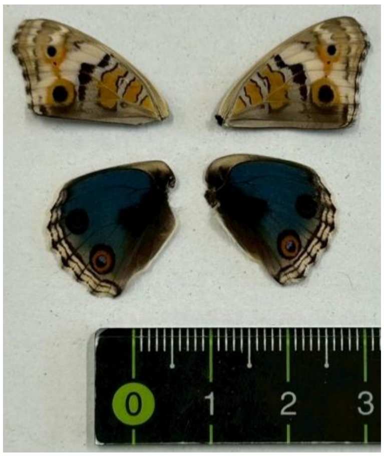

NO3

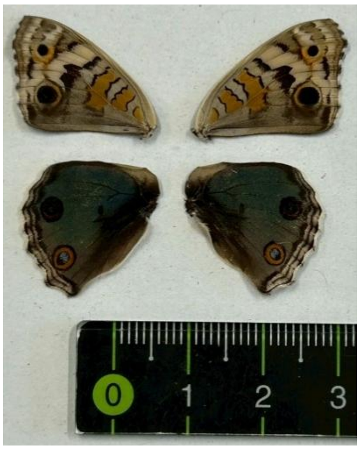

NO4

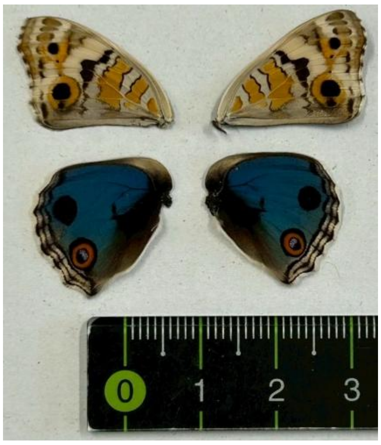

NO5

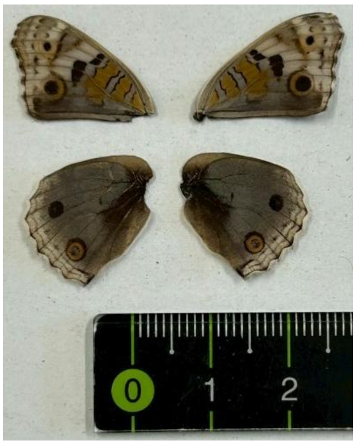

NO6

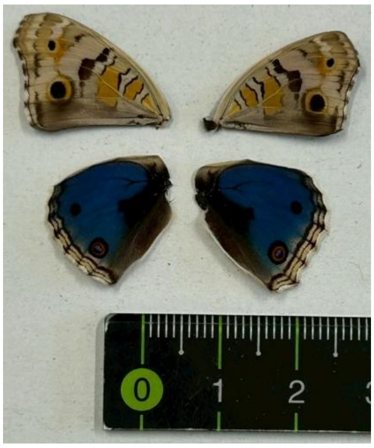

Supplement: Supplementary file 1 [file insects-17-01032-s001.zip › Supplementary Figures S1/Catalase/Sandwich/Catalase 800 ng sand male.pdf]
