# Supplementary material for: Hydrogen Peroxide Regulates Butterfly Wing Eyespot Color Patterns
Source: Insects. 2026 Oct 8;17(10):1032. doi: 10.3390/insects17101032 (PMC13705226; doi:10.3390/insects17101032)

Dimethyl thiourea 108.04 mM *Junonia orithya* Male Ventral

NO1

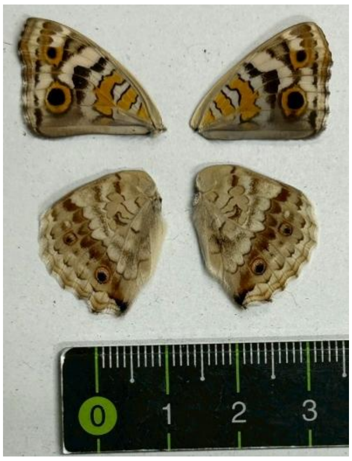

NO2

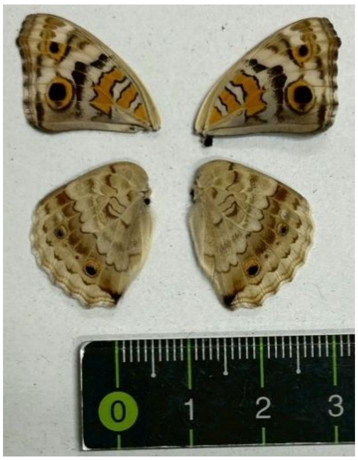

NO3

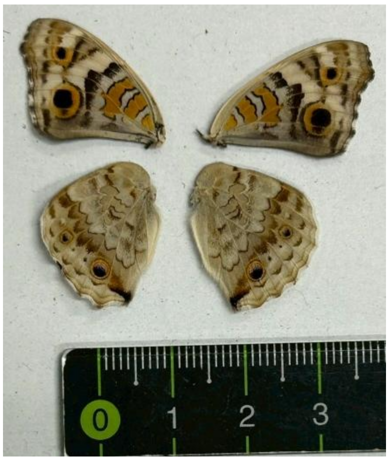

NO4

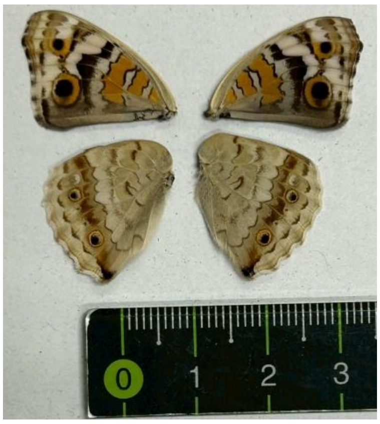

NO5

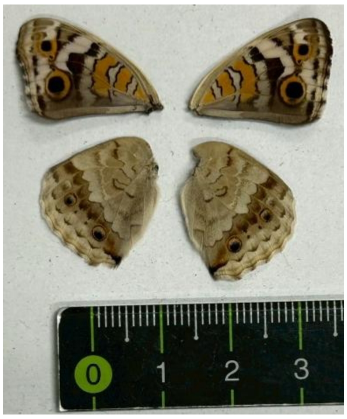

NO6

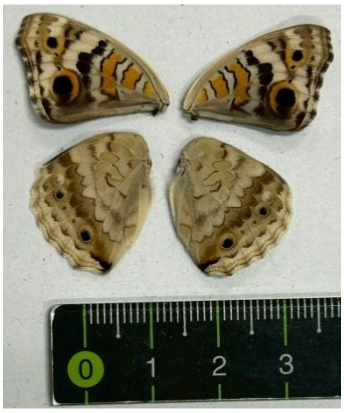

NO7

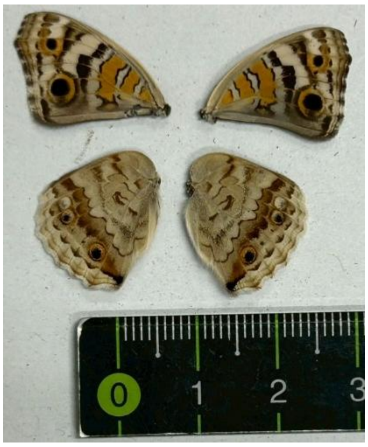

NO8

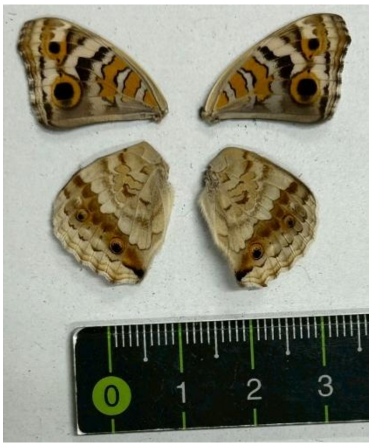

NO9

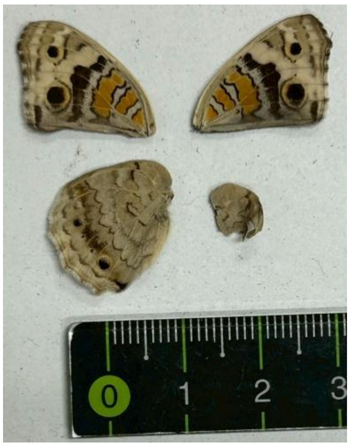

NO10

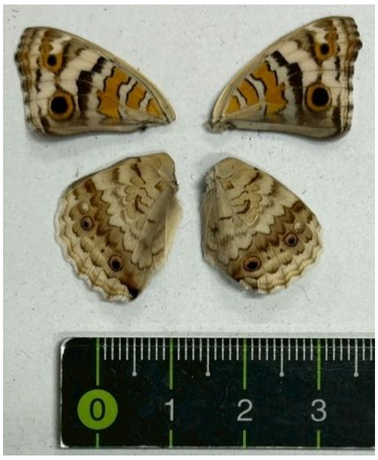

NO11

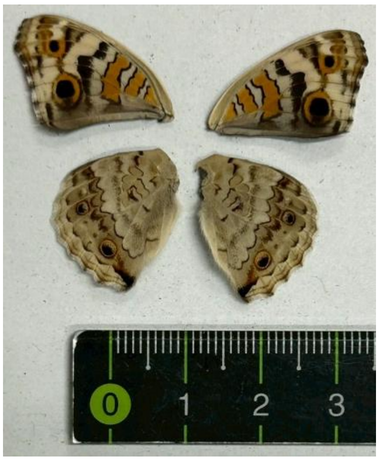

NO12

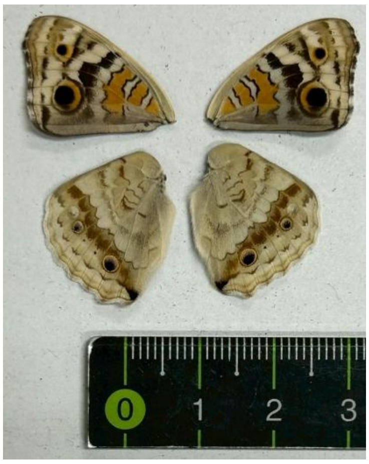

NO13

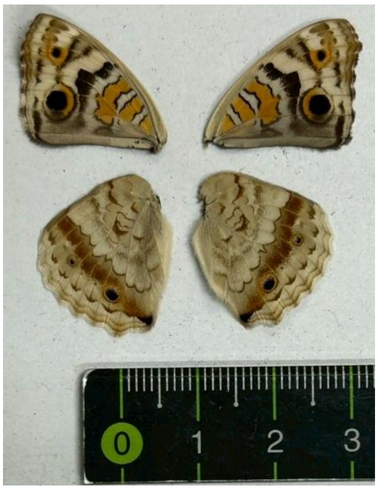

NO14

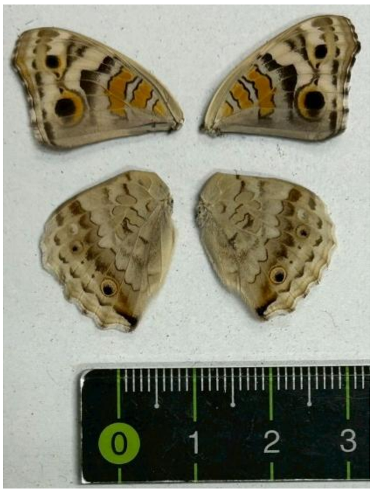

NO15

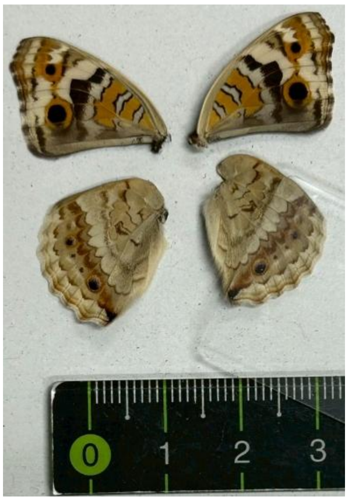

NO16

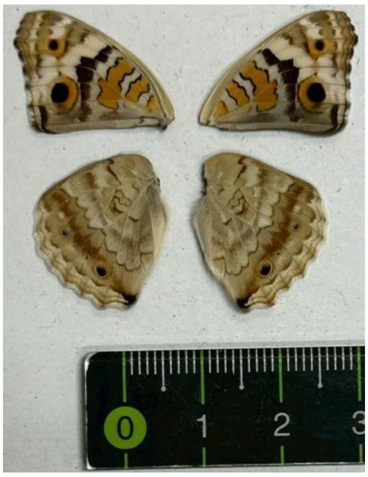

NO17

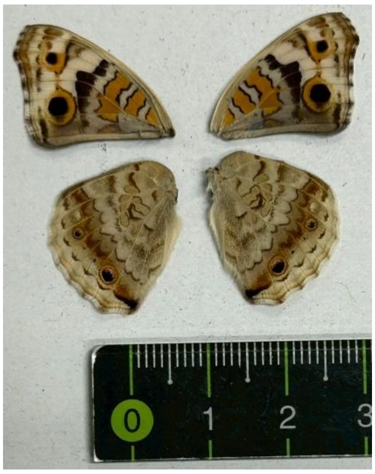

NO18

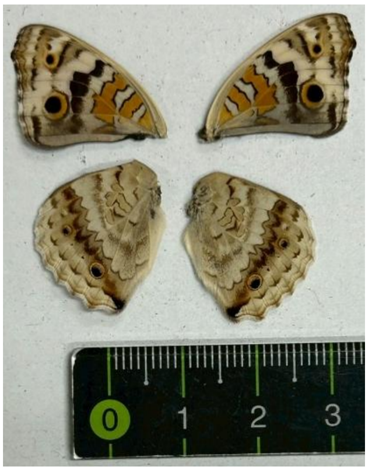

NO19

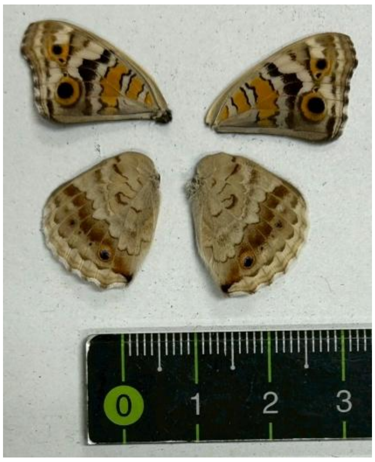

NO20

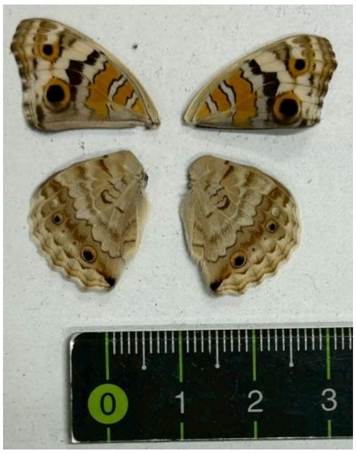

NO21

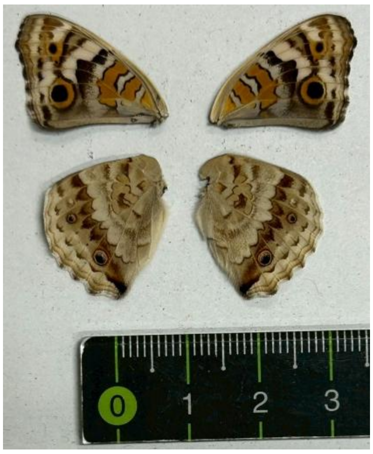

Supplement: Supplementary file 1 [file insects-17-01032-s001.zip › Supplementary Figures S1/DMTU/Injection/DMTU Part1/Dimethyl thiourea 108.04 mM junonia orithya Male Ventral.pdf]
