# Supplementary material for: Hydrogen Peroxide Regulates Butterfly Wing Eyespot Color Patterns
Source: Insects. 2026 Oct 8;17(10):1032. doi: 10.3390/insects17101032 (PMC13705226; doi:10.3390/insects17101032)

Dimethyl thiourea 1080.4 mM, 108.04 mM, 10.804 mM Ctrl *Junonia orithya* Male Ventral

NO1

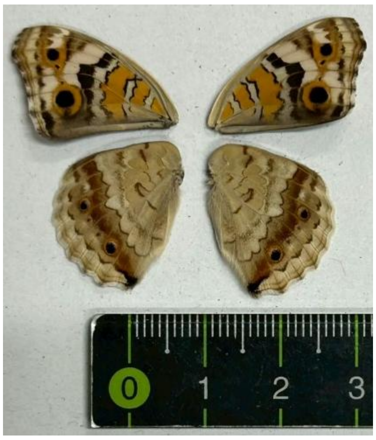

NO2

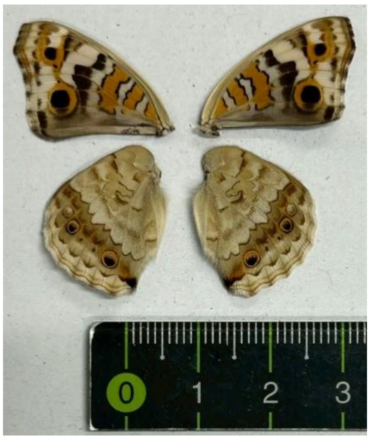

NO3

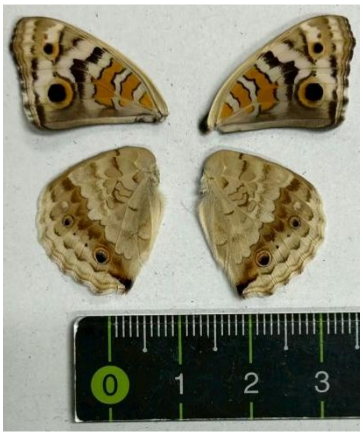

NO4

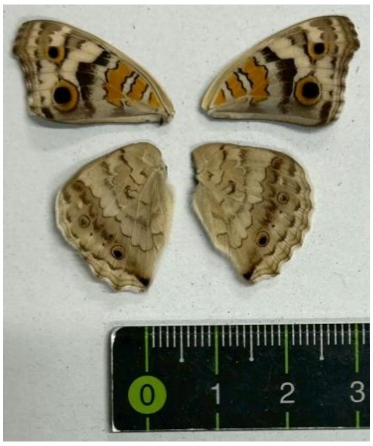

NO5

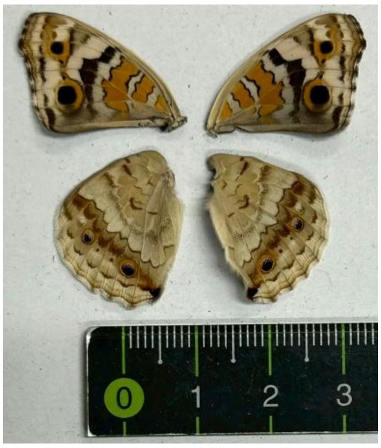

NO6

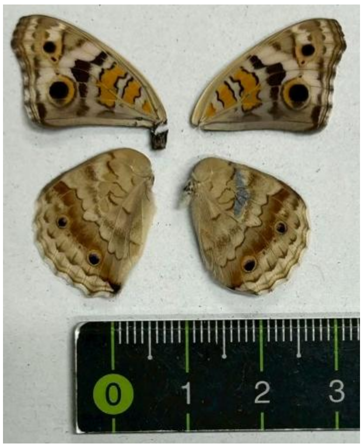

NO7

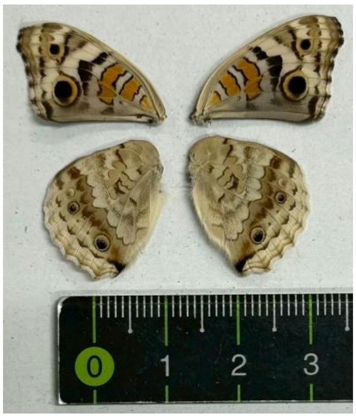

NO8

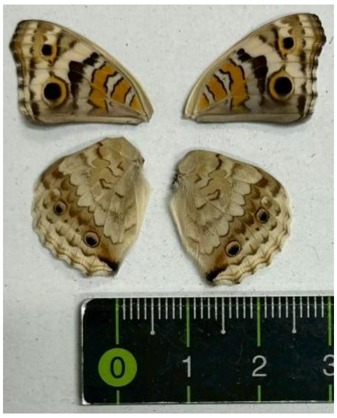

NO9

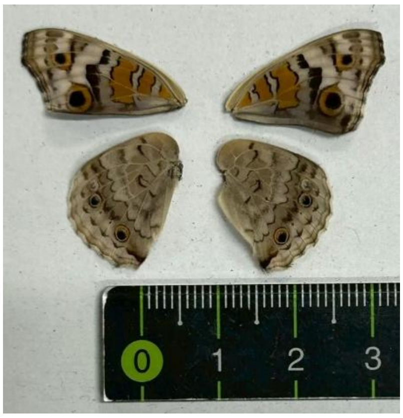

NO10

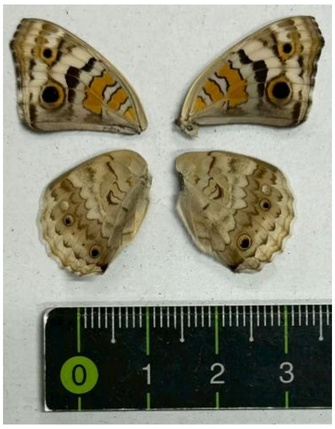

NO11

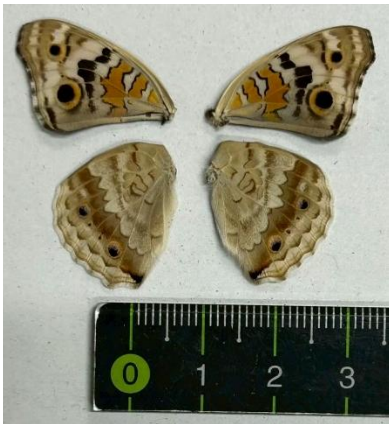

NO12

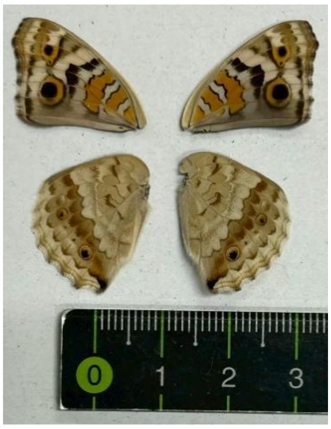

NO13

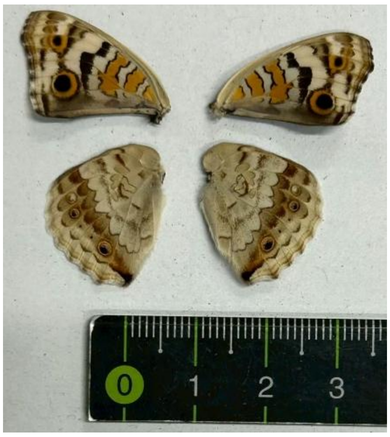

NO14

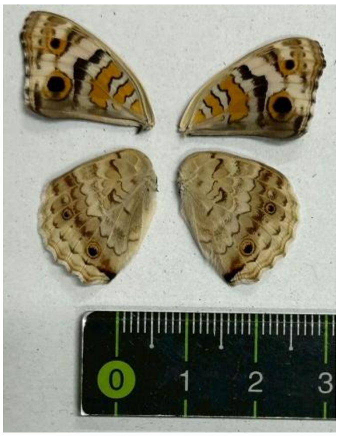

NO15

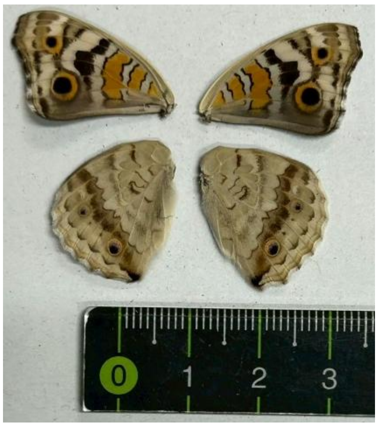

NO16

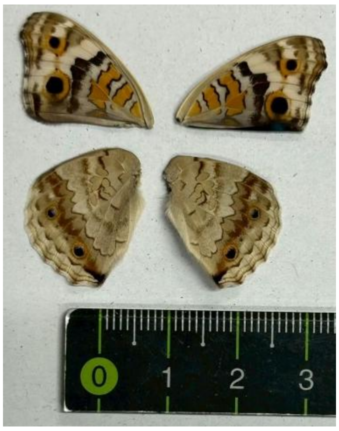

NO17

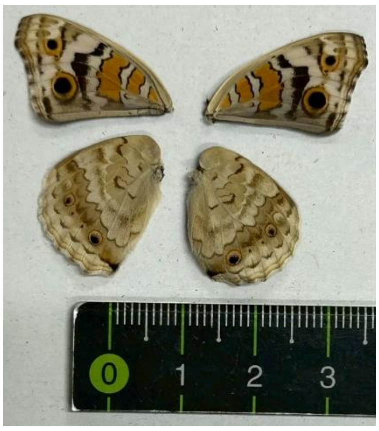

NO18

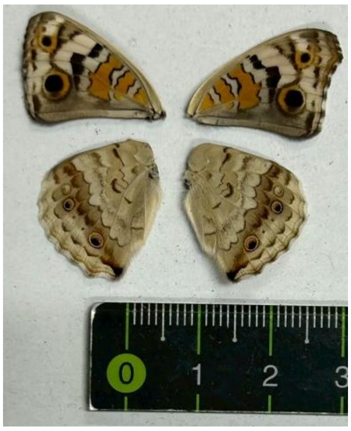

NO19

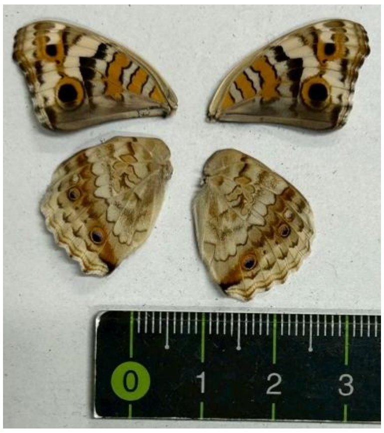

NO20

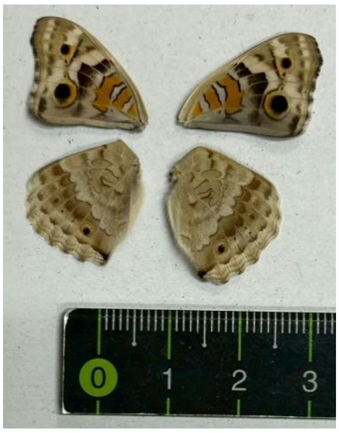

NO21

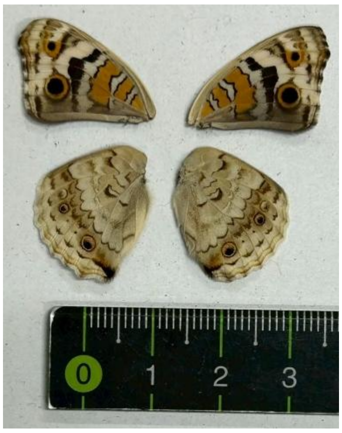

NO22

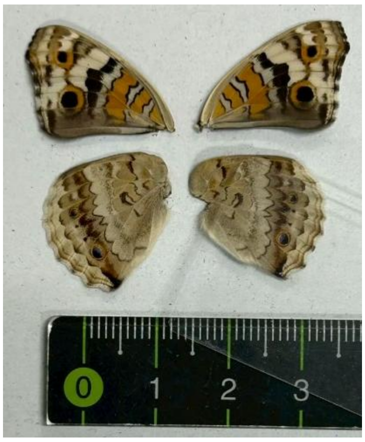

NO23

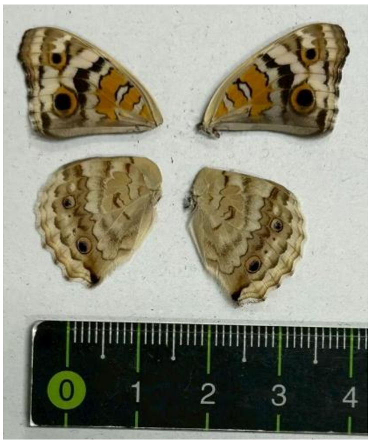

NO24

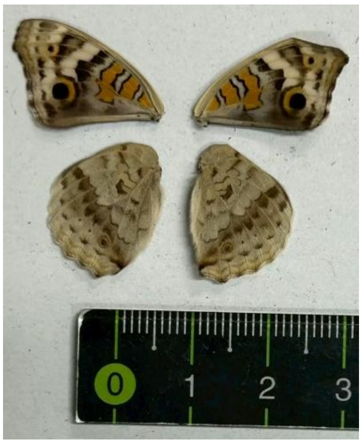

NO25

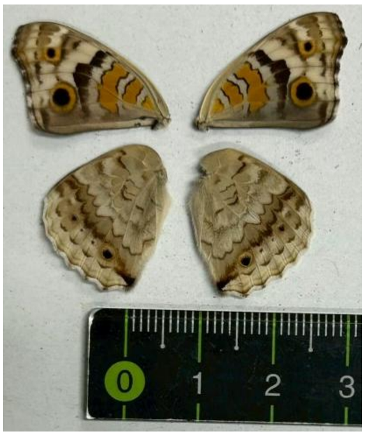

NO26

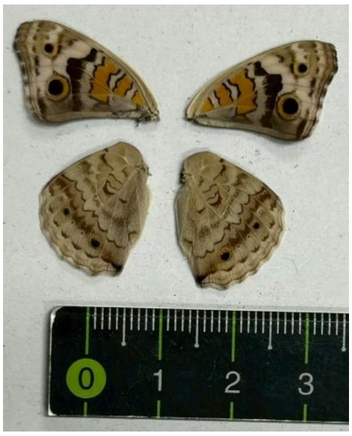

NO27

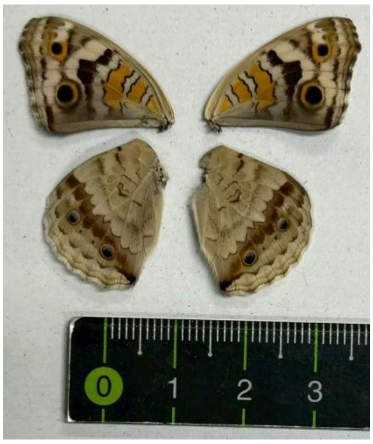

NO28

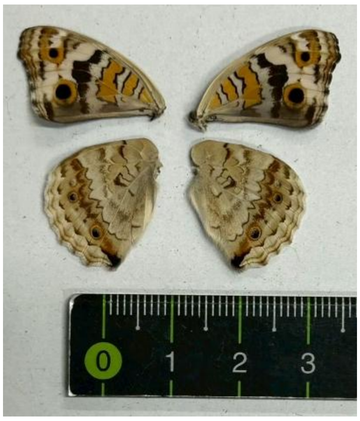

NO29

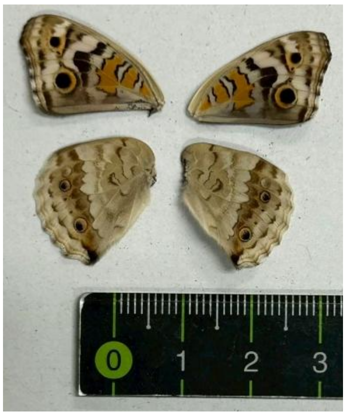

NO30

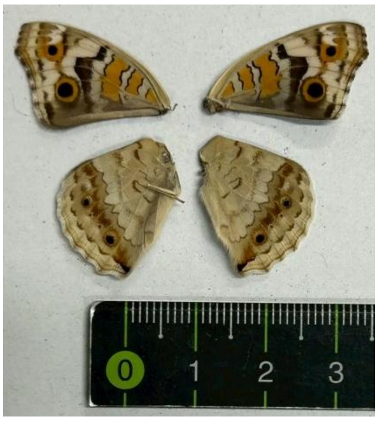

NO31

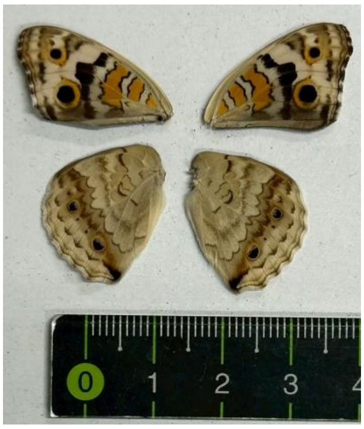

NO32

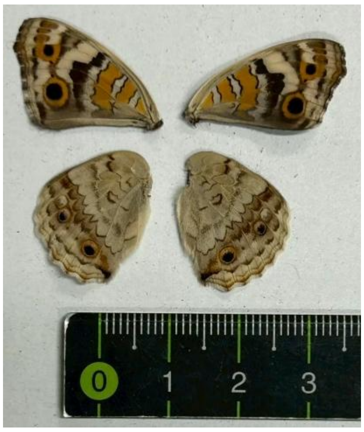

NO33

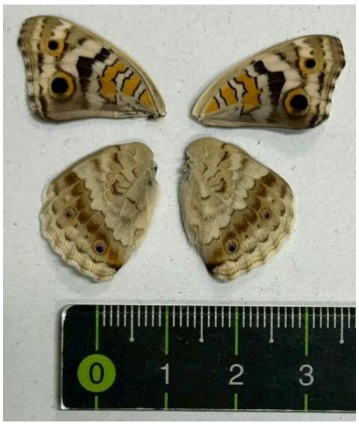

NO34

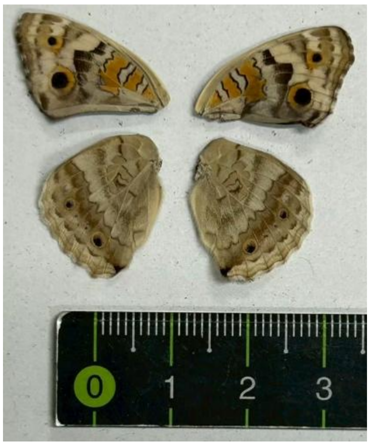

NO35

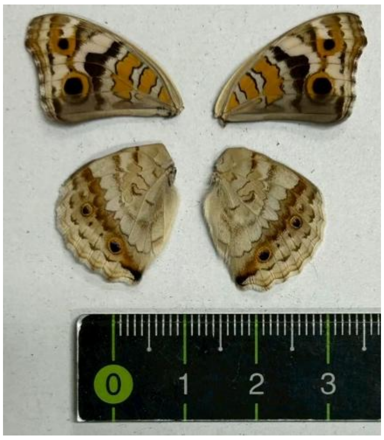

NO36

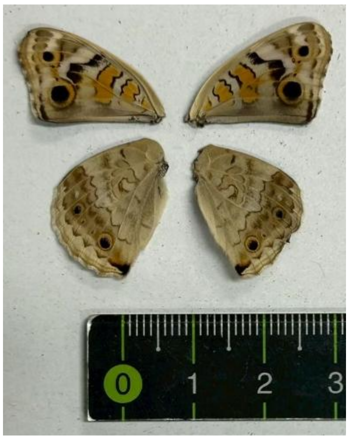

NO37

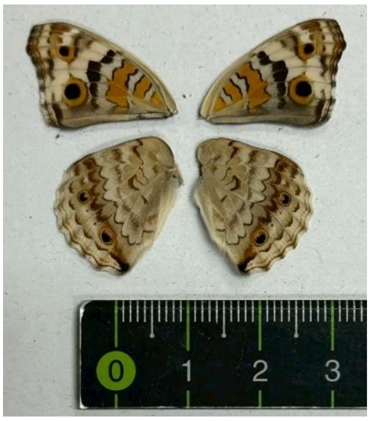

NO38

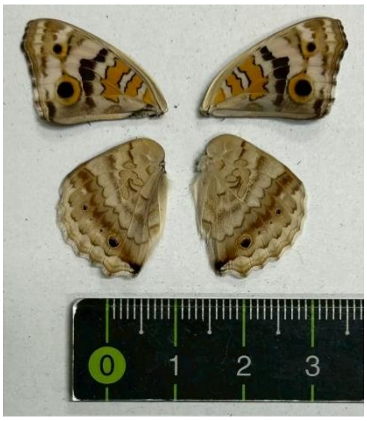

NO39

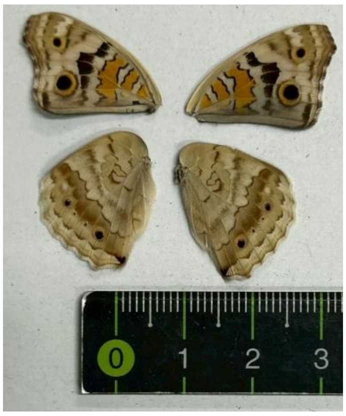

NO40

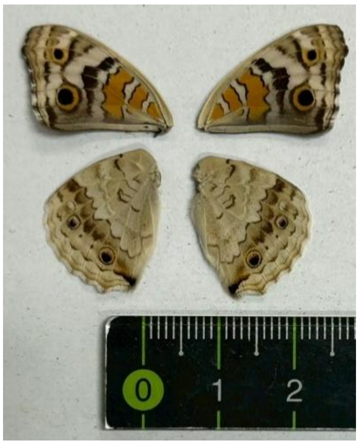

NO41

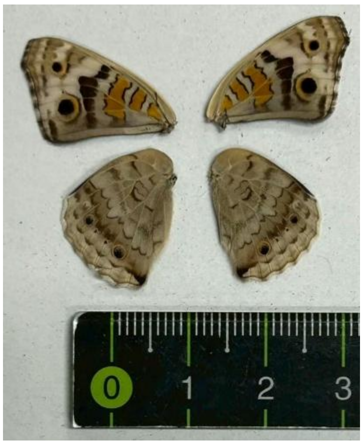

NO42

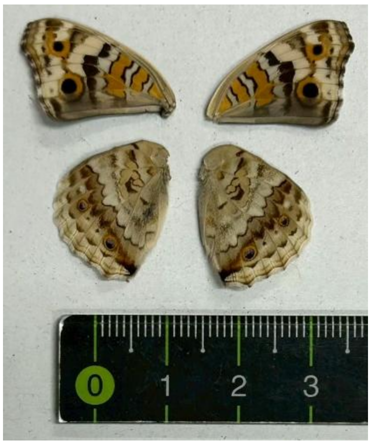

NO43

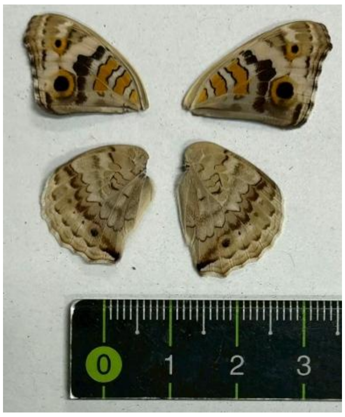

NO44

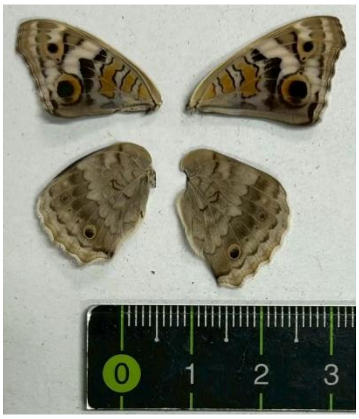

NO45

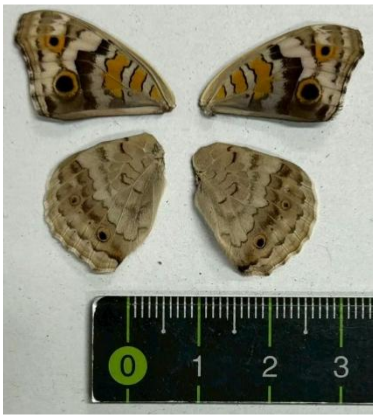

NO46

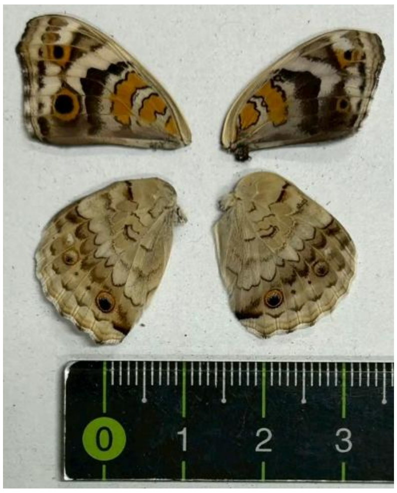

Supplement: Supplementary file 1 [file insects-17-01032-s001.zip › Supplementary Figures S1/DMTU/Injection/DMTU Part1/Dimethyl thiourea 1080.4 mM, 108.04 mM, 10.804 mM Ctrl junonia orithya Male Ventral.pdf]
