# Supplementary material for: Hydrogen Peroxide Regulates Butterfly Wing Eyespot Color Patterns
Source: Insects. 2026 Oct 8;17(10):1032. doi: 10.3390/insects17101032 (PMC13705226; doi:10.3390/insects17101032)

Dimethyl thiourea 216.08 mM, 432.16 mM, Ctrl *Junonia orithya* Female Ventral

NO1

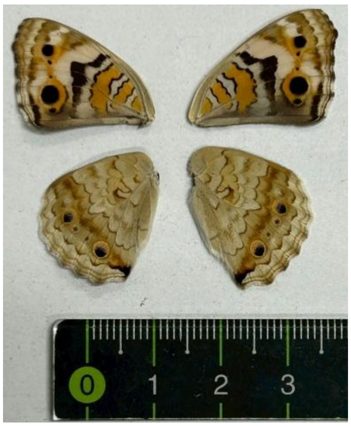


NO2

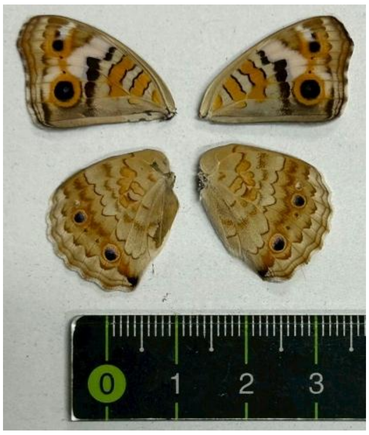


NO3

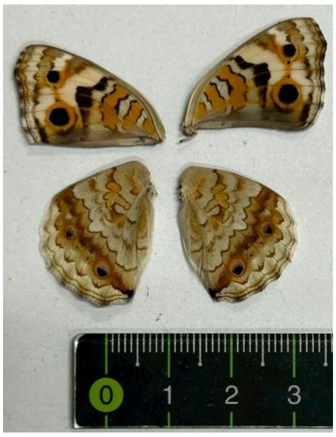


NO4

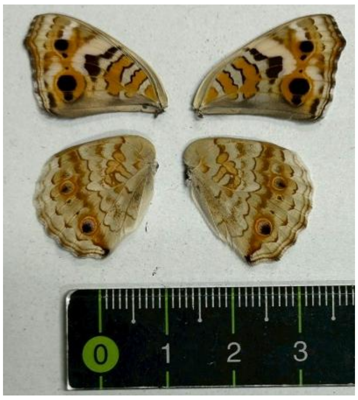


NO5

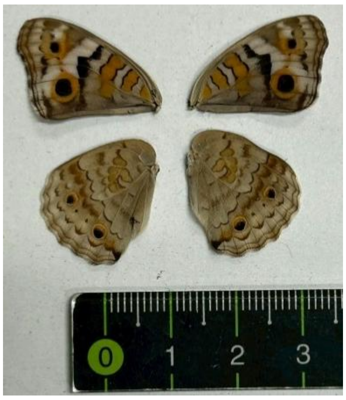


NO6

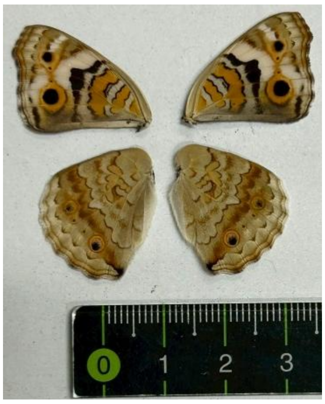


NO7

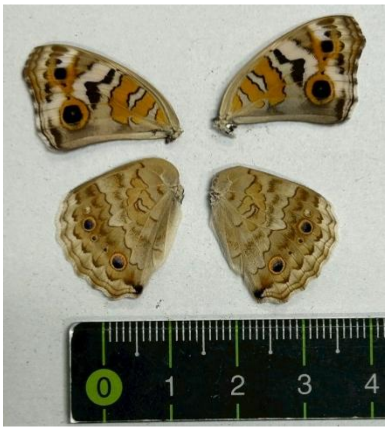


NO8

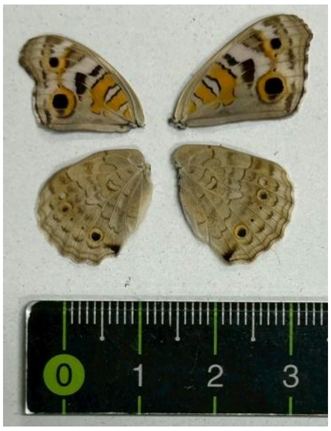


NO9

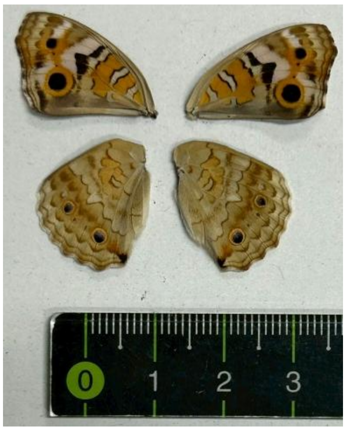

NO10

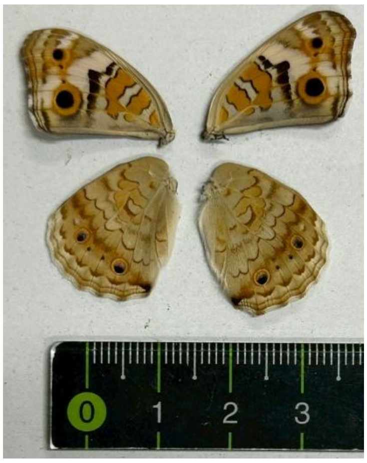

Supplement: Supplementary file 1 [file insects-17-01032-s001.zip › Supplementary Figures S1/DMTU/Injection/DMTU Part2/Dimethyl thiourea 216.08 mM, 432.16 mM, Ctrl junonia orithya Female Ventral.pdf]
