# Supplementary material for: Hydrogen Peroxide Regulates Butterfly Wing Eyespot Color Patterns
Source: Insects. 2026 Oct 8;17(10):1032. doi: 10.3390/insects17101032 (PMC13705226; doi:10.3390/insects17101032)

Hydrogen peroxide 15% *Junonia orithya* Male Dorsal

NO1

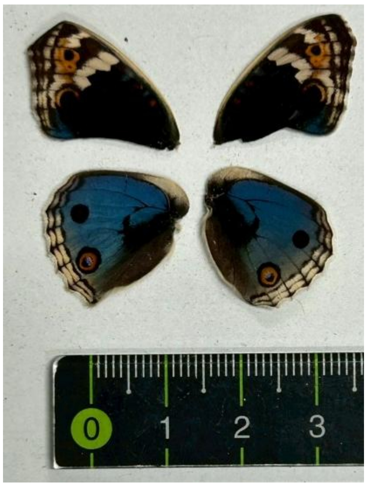

NO2

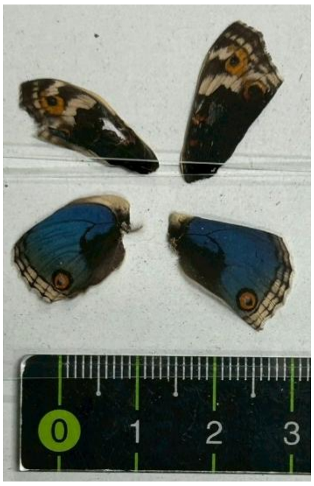

NO3

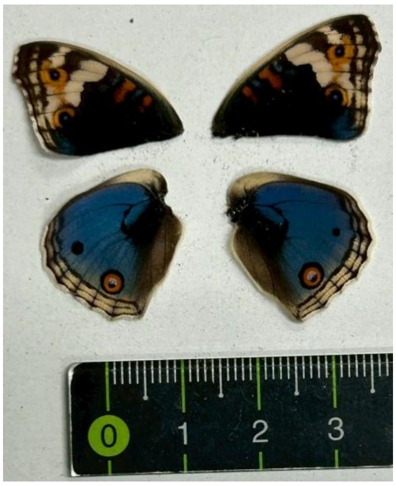

NO4

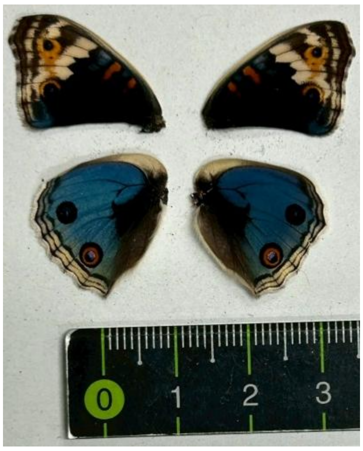

NO5

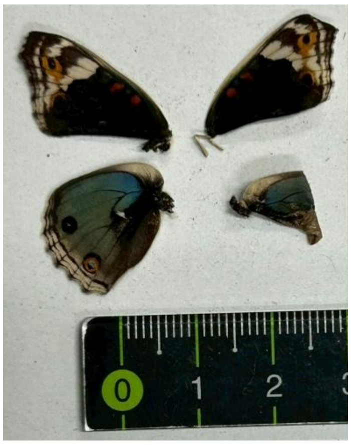

NO6

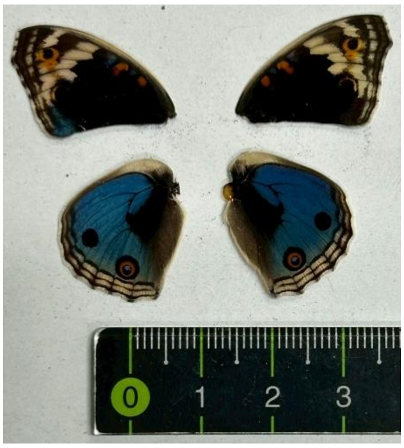

Supplement: Supplementary file 1 [file insects-17-01032-s001.zip › Supplementary Figures S1/Hydrogen peroxide/Injection/H2O2 Part2/Hydrogen peroxide 15% junonia orithya Male Dorsal.pdf]
