# Supplementary material for: Hydrogen Peroxide Regulates Butterfly Wing Eyespot Color Patterns
Source: Insects. 2026 Oct 8;17(10):1032. doi: 10.3390/insects17101032 (PMC13705226; doi:10.3390/insects17101032)

Hydrogen Peroxide 3% Female

NO1

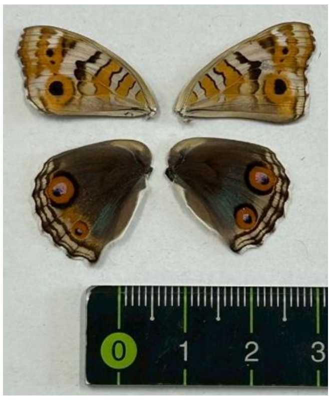

NO2

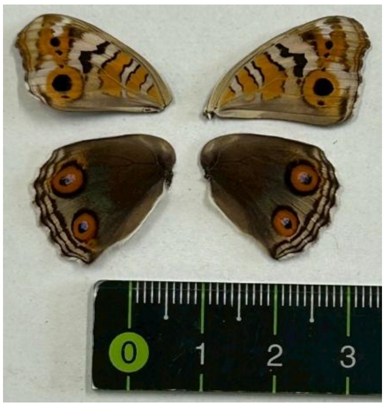

NO3

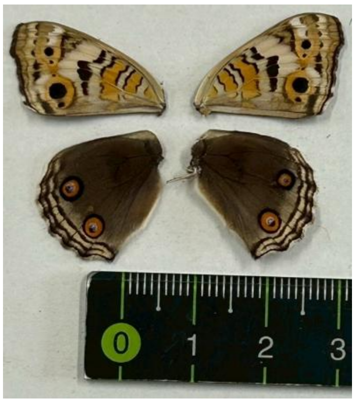

NO4

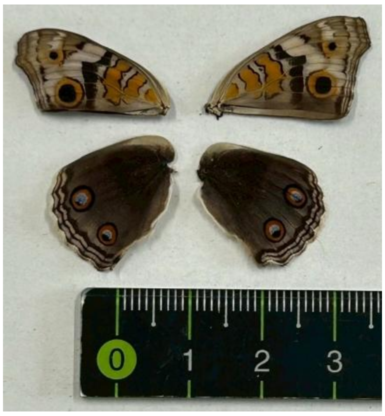

NO5

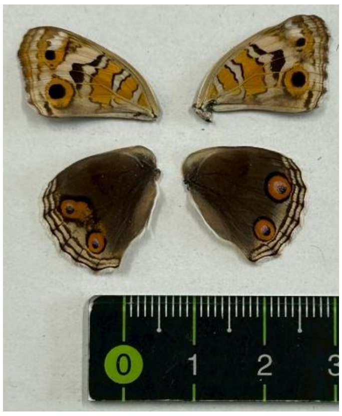

NO6

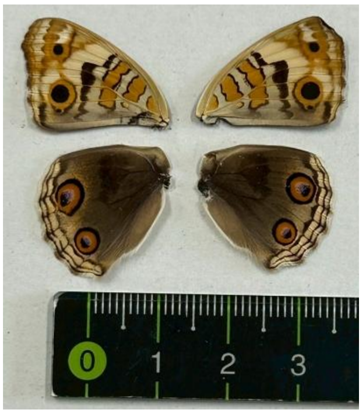

NO7

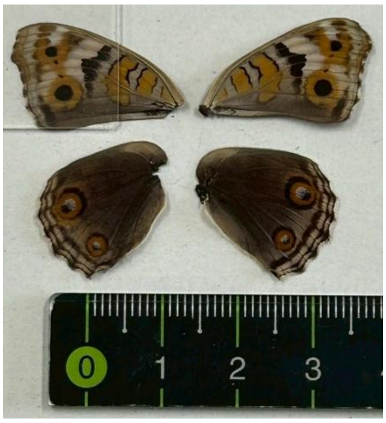

NO8

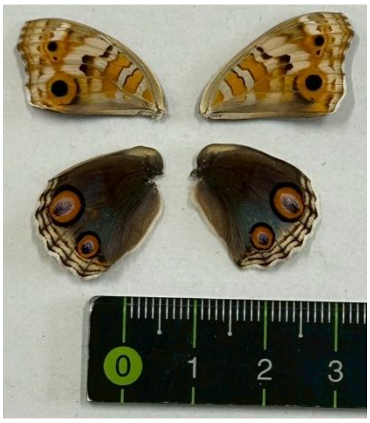

NO9

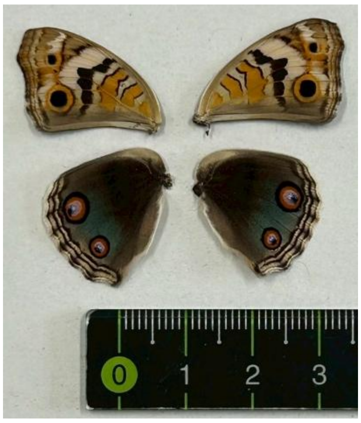

NO10

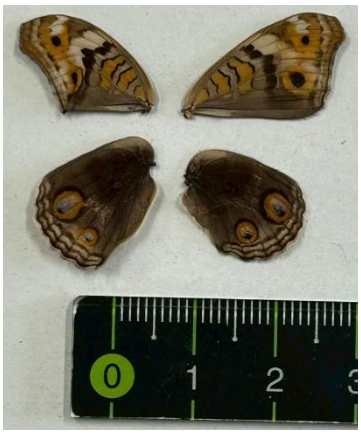

NO11

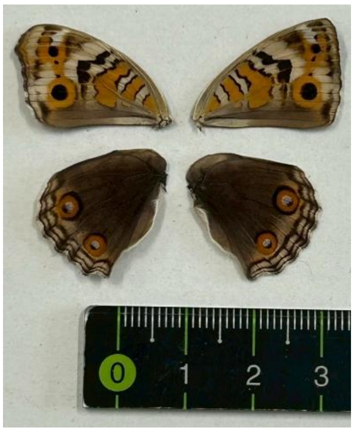

NO12

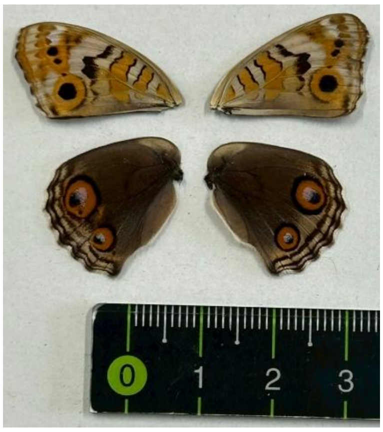

NO13

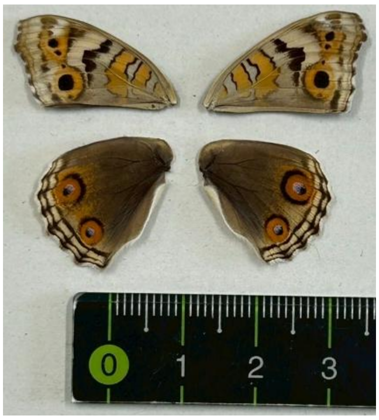

NO14

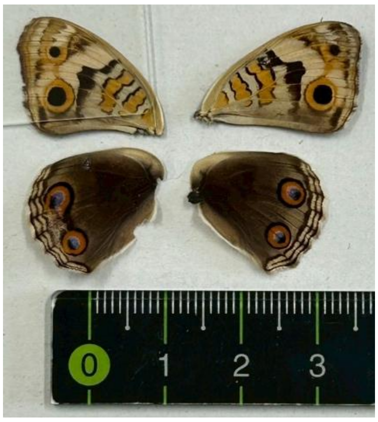

NO15

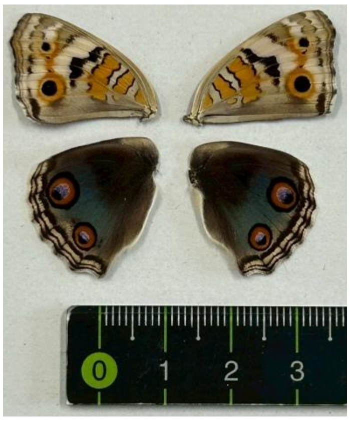

NO16

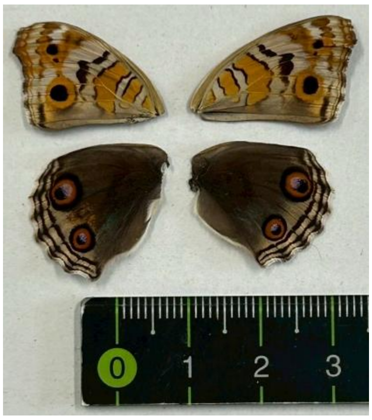

NO17

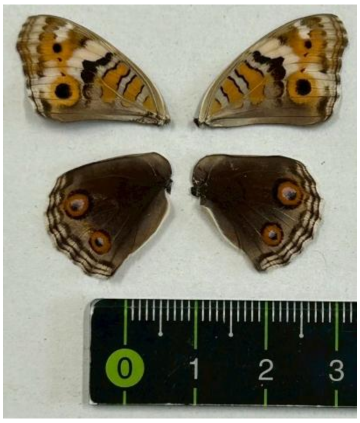

NO18

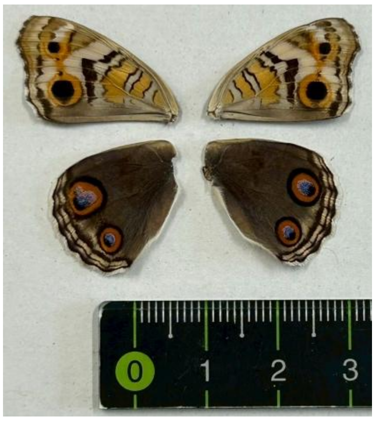

NO19

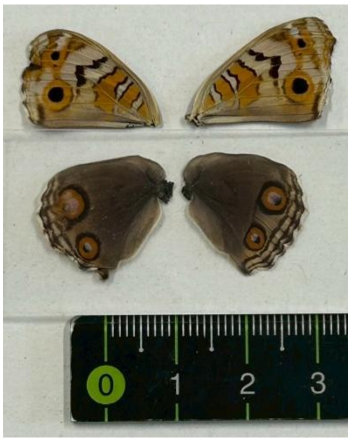

NO20

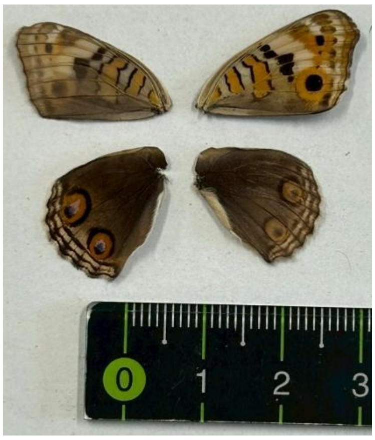

NO21

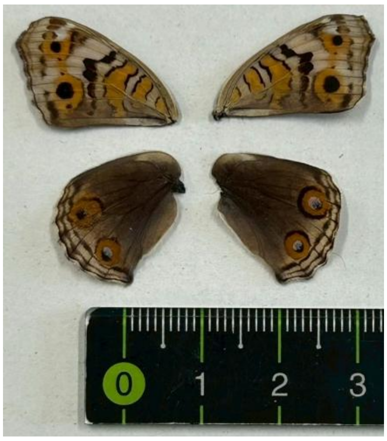

NO22

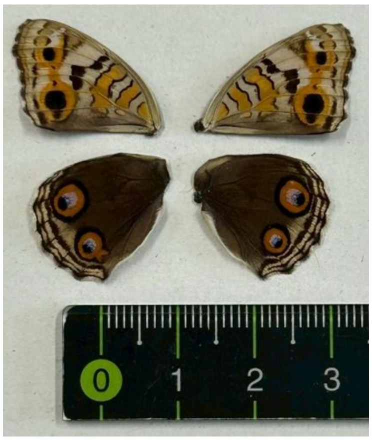

NO23

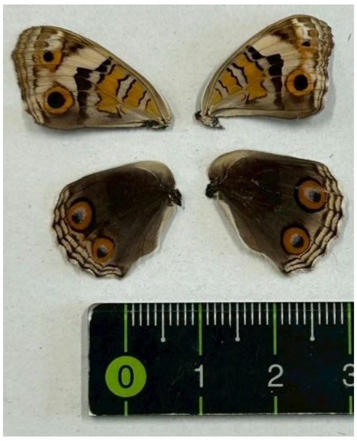

NO24

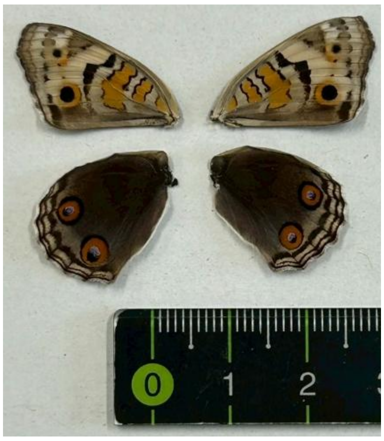

NO25

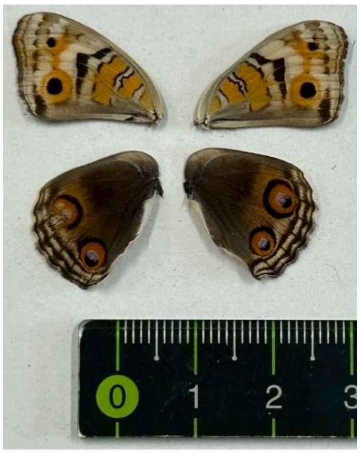

NO26

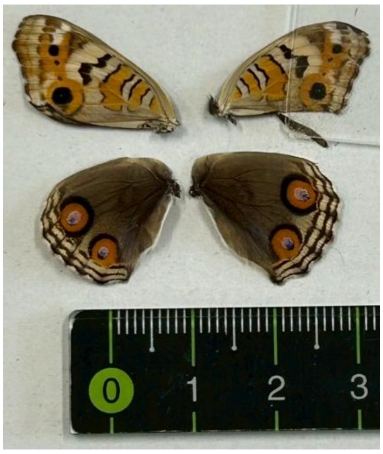

NO27

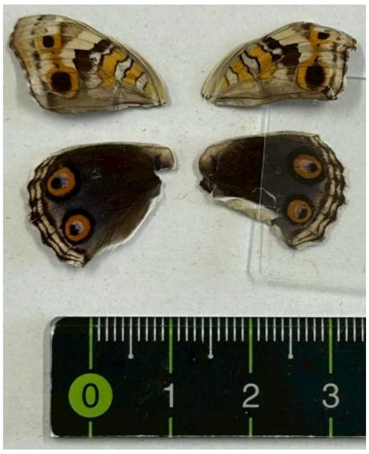

NO28

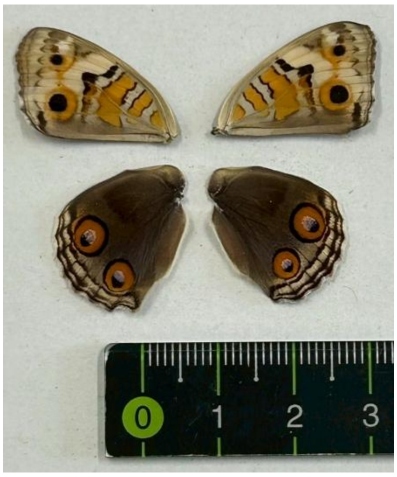

NO29

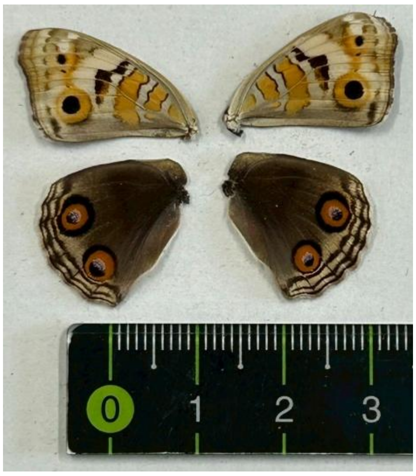

Supplement: Supplementary file 1 [file insects-17-01032-s001.zip › Supplementary Figures S1/Hydrogen peroxide/Sandwich/H2O2 sand 3% female for paper.pdf]
