# Supplementary material for: Hydrogen Peroxide Regulates Butterfly Wing Eyespot Color Patterns
Source: Insects. 2026 Oct 8;17(10):1032. doi: 10.3390/insects17101032 (PMC13705226; doi:10.3390/insects17101032)

PBS *Junonia orithya* Female Ventral

NO1

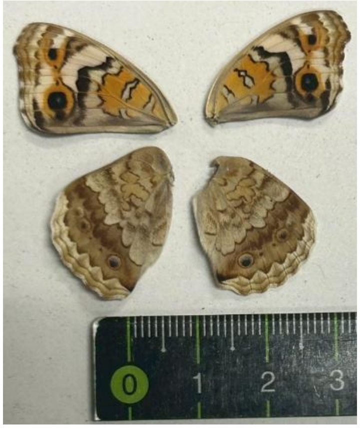

NO2

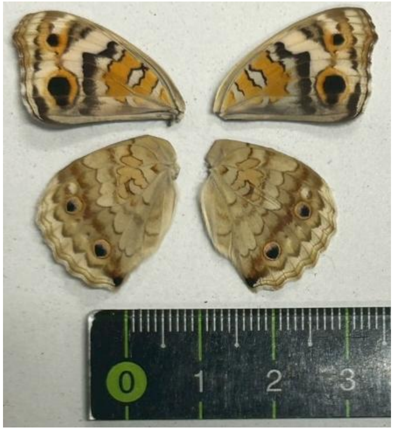

NO3

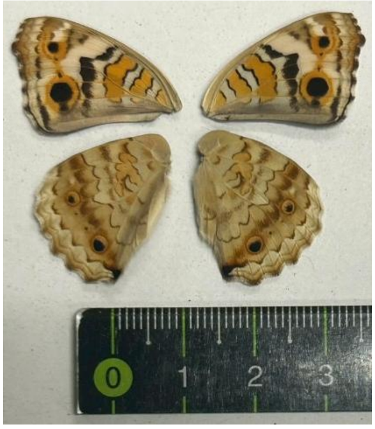

NO4

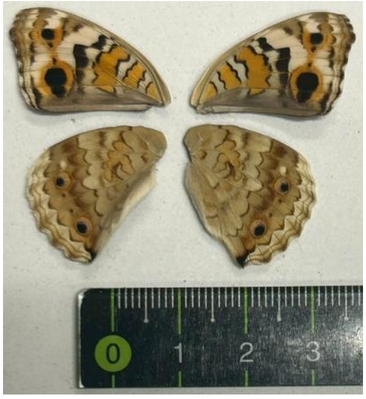

NO5

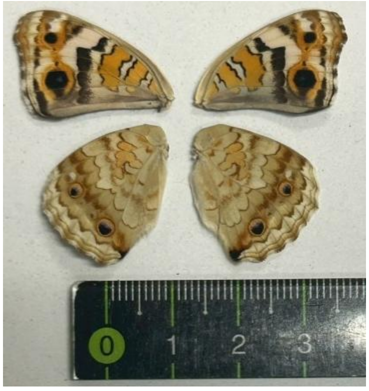

NO6

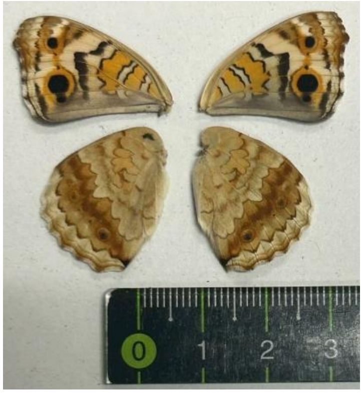

NO7

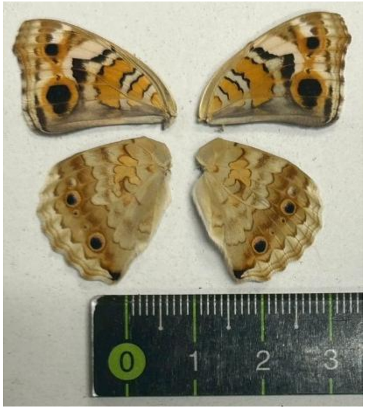

NO8

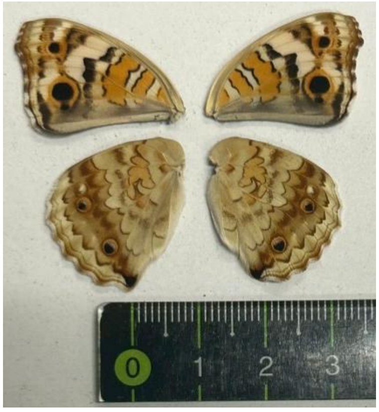

NO9

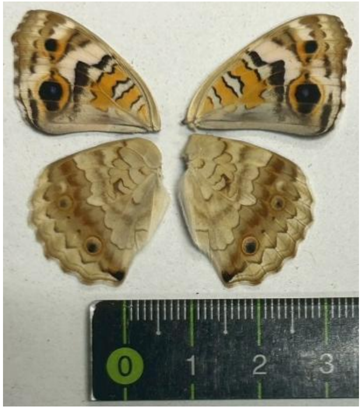

Supplement: Supplementary file 1 [file insects-17-01032-s001.zip › Supplementary Figures S1/Water and PBS/PBS junonia orithya Female Ventral.pdf]
