# Supplementary material for: Hydrogen Peroxide Regulates Butterfly Wing Eyespot Color Patterns
Source: Insects. 2026 Oct 8;17(10):1032. doi: 10.3390/insects17101032 (PMC13705226; doi:10.3390/insects17101032)

Ctrl *Junonia almana* Dorsal

NO1

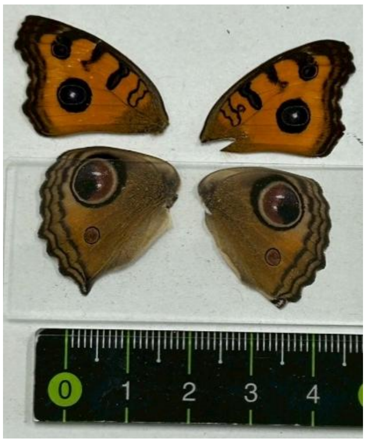

NO2

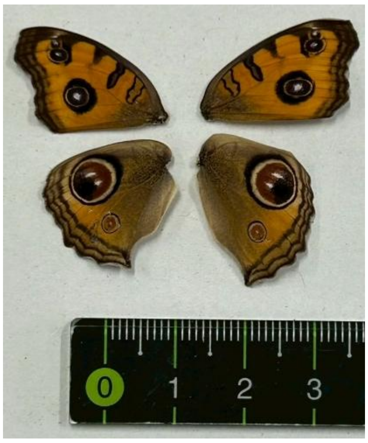

NO3

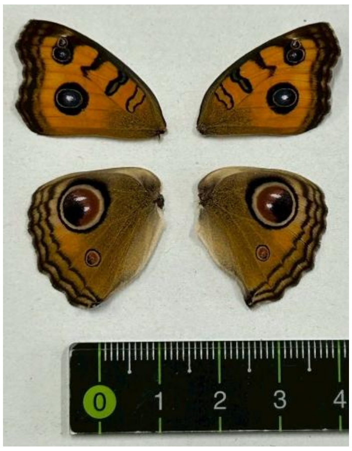

NO4

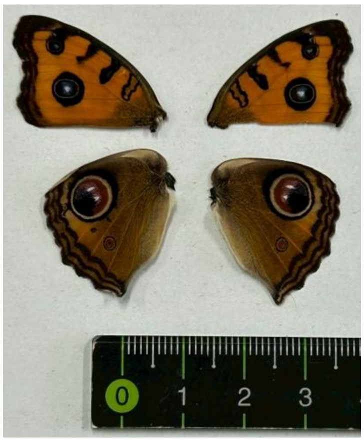

NO5

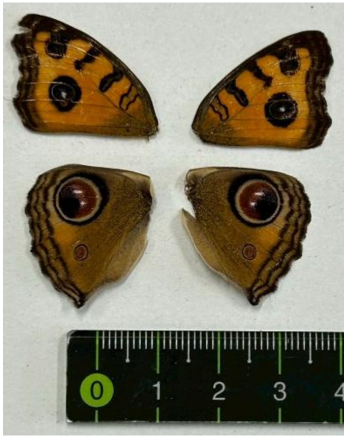

NO6

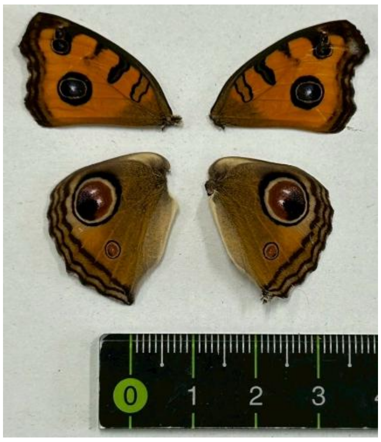

NO7

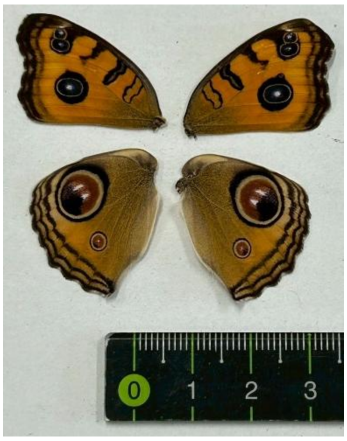

NO8

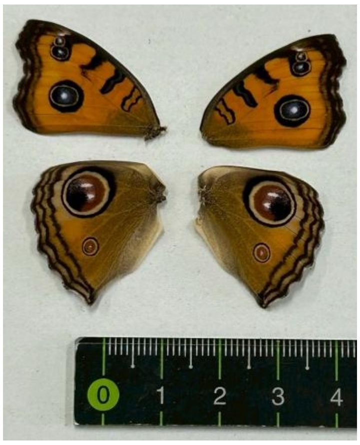

NO9

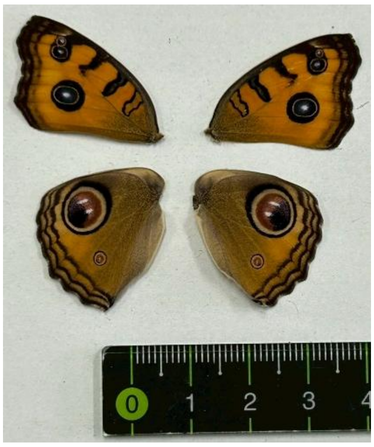

NO10 NO11 NO12

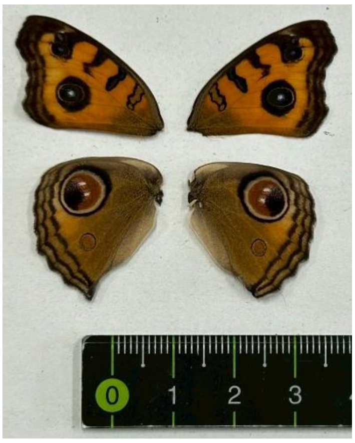

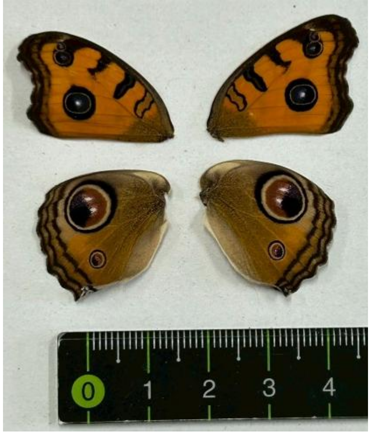

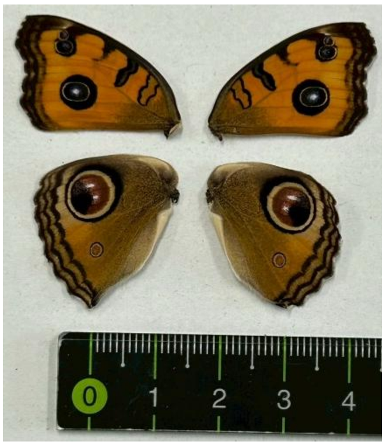

NO13 NO14 NO15

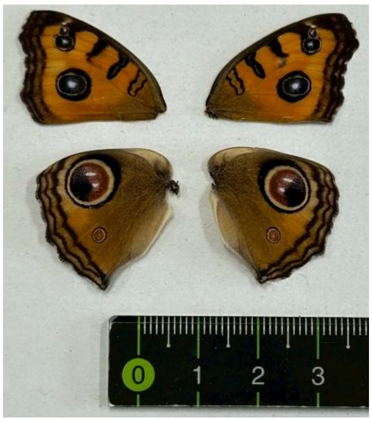

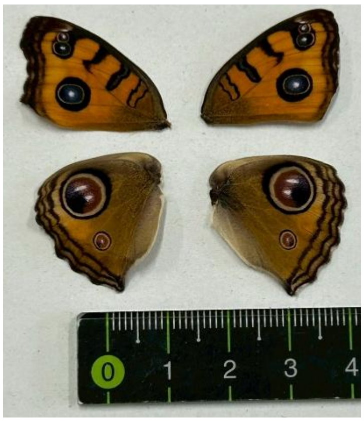

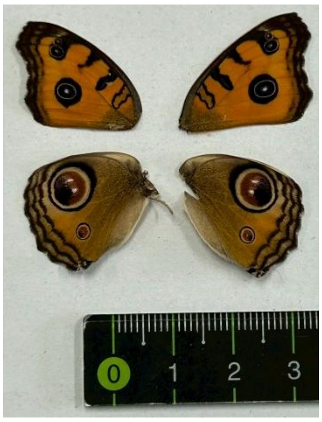

NO16 NO17 NO18

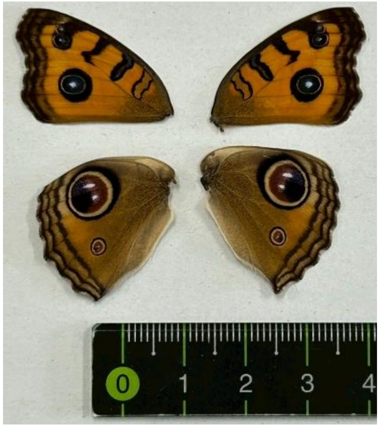

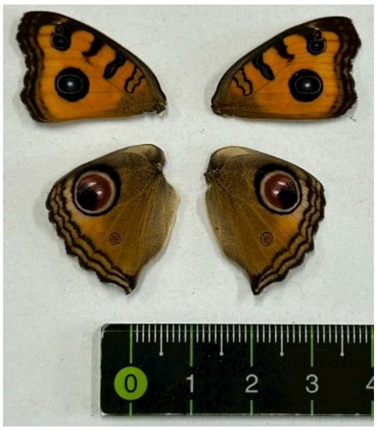

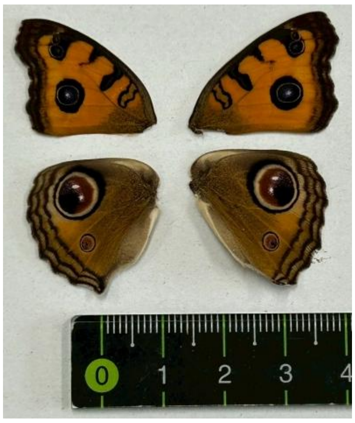

NO19

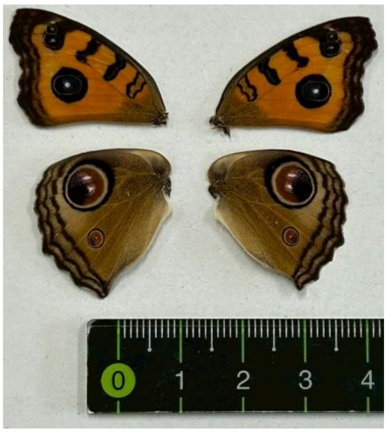

NO20

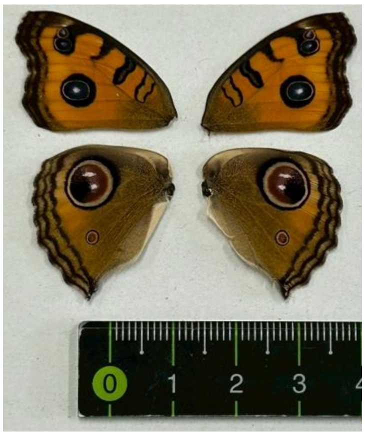

NO21

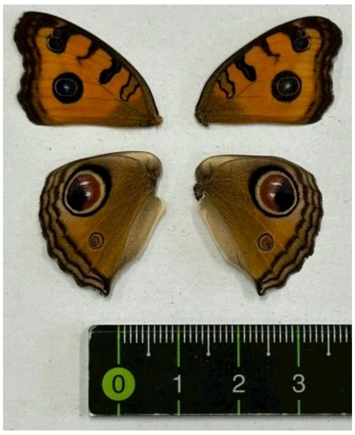

NO22

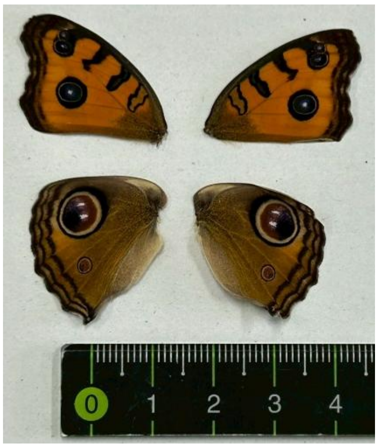

NO23

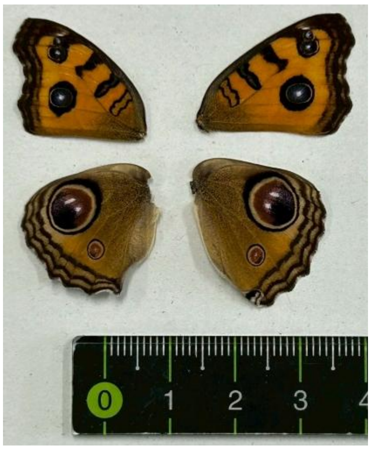

NO24

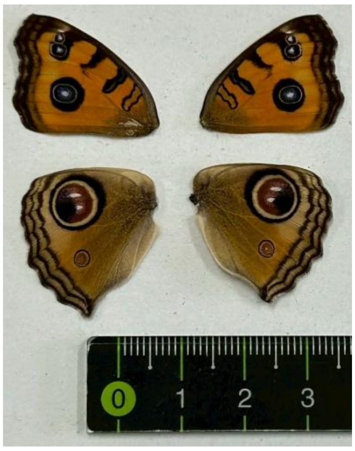

NO25

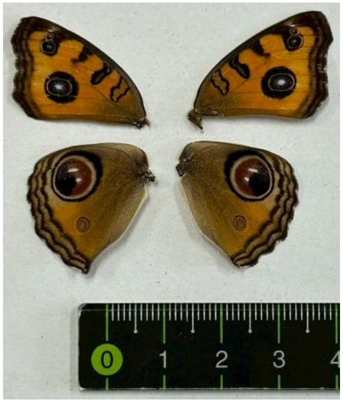

NO26

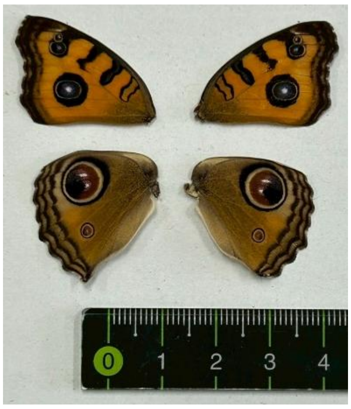

NO27

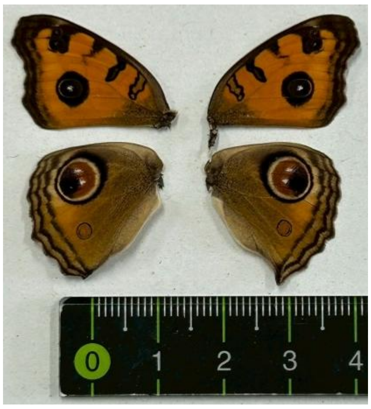

NO28

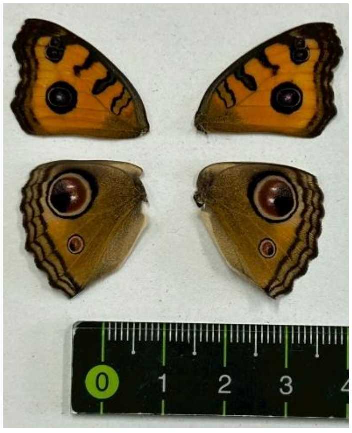

NO29

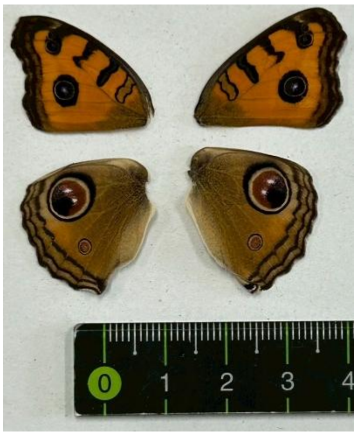

NO30

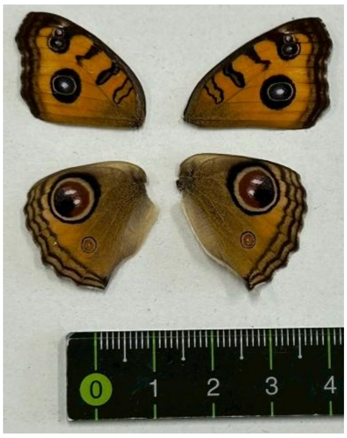

NO31

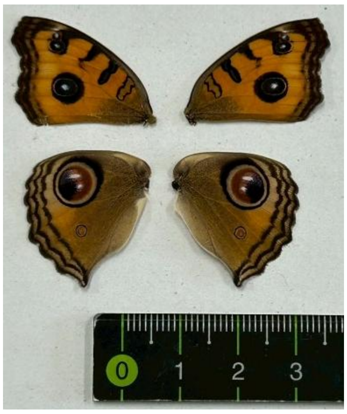

NO32

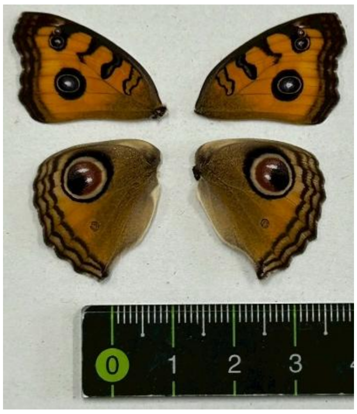

Supplement: Supplementary file 1 [file insects-17-01032-s001.zip › Supplementary Figures S2/Ctrl junonia almana Dorsal.pdf]
